# Spectroscopic and Theoretical Analysis of *N*‑Ethylphenothiazine Derivatives: Alkyl Chain Length Effects on Solvatochromism, Fluorescence Efficiency, and Excited-State Polarity

**DOI:** 10.1021/acs.jpcb.5c08011

**Published:** 2026-02-25

**Authors:** Aneta Slodek, Dawid Zych, Sylwia Zimosz, Grażyna Szafraniec-Gorol, Sonia Kotowicz, Martyna Kubis, Katarzyna Kowalska-Szojda, Katarzyna Malarz, Robert Musioł

**Affiliations:** † 431562 Institute of Chemistry, University of Silesia, Szkolna 9, Katowice 40-006, Poland; ‡ Institute of Chemistry, Faculty of Chemistry and Pharmacy, 427072 University of Opole, Oleska 48, Opole 45-052, Poland; § Institute of Physics, Faculty of Science and Technology, 49568 University of Silesia, 75 Pułku Piechoty 1A, Chorzów 41-500, Poland

## Abstract

The newly symmetrically substituted by carbazole units *N*-ethylphenothiazine derivatives **4a**–**4g**, which vary in the length of the alkyl chain at the carbazole substituents, were designed and synthesized. By methodically investigating the impact of the alkyl chain at the carbazole unit on the photophysical properties of these novel compounds, with support from TD/DFT calculations, their photophysical characteristics were analyzed across a range of solvents with varying polarity. In hexane through to acetonitrile, the fluorescent properties of compounds **4a**–**4g** result in light emission from blue to pale green. The moderate charge-transfer in compounds is indicated by the positive solvatochromism observed in their emission spectra. The derivatives with medium-length alkyl chains (C4–C6) demonstrated optimal photophysical performance, including high quantum yields (up to 81%), long excited-state lifetimes, and strong absorption coefficients. Low-temperature measurements confirmed the persistence of fluorescence and the emergence of phosphorescence bands. Dipole moment analysis using Lippert–Mataga plots revealed a gradual increase in excited-state polarity with increasing alkyl chain length. Cytotoxicity tests confirmed that all derivatives were nontoxic up to 25 μM across various cancer and normal cell lines. Despite limited passive membrane permeability, fluorescence microscopy under mild permeabilizing conditions (Tween 20) revealed that derivatives **4b** and **4c** could serve as selective fluorescent probes for membrane integrity. The results underscore the importance of alkyl chain engineering in tailoring molecular properties, making these compounds promising candidates for optoelectronic and photonic applications. They hold promise for bioimaging applications, particularly in membrane-targeted diagnostics or responsive drug-delivery systems.

## Graphical abstract

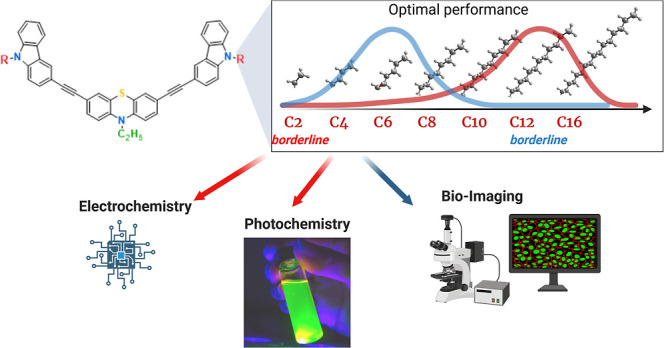

## 1 Introduction

Donor–acceptor (D–A) and more extensive systems have become increasingly desirable in recent years.^1,2^ Due to their properties, they are utilized in numerous scientific disciplines.^3–5^ What makes them so exceptional? Compared to metal-containing dyes, these metal-free organic dyes offer several advantages, including high efficiency, high thermal stability, lower cost, less negative environmental impact, a higher molar extinction coefficient, and a wide range of molecular structures.^6–9^ There is an intermolecular charge transfer (ICT) in D–A systems.^10^ It provides the energy differential between the highest occupied molecular orbital (HOMO) and the lowest unoccupied molecular orbital (LUMO) in a molecule, which is intrinsic to the fluorescence phenomenon.^11^ It has been demonstrated that a heterocyclic ring increases the efficacy of such systems.^12,13^

The heterocyclic compound phenothiazine is known as a potent electron-donating group. In addition to the nitrogen atom, it also contains an electron-rich sulfur atom. As emission layers in organic light-emitting diodes (OLEDs) and active layers in dye-sensitized solar cells,^14–18^ phenothiazine derivatives acetylene-bridged with electron acceptor/donor molecules are widely used. Additionally, they are widely used in medicine and pharmacology.^19–21^ In addition, research indicates that phenothiazine derivatives frequently exhibit solvatochromic behavior.^22–25^ The recent synthesis and characterization of a series of phenothiazines combined with arylate substitutes of varying electronic properties. The compounds’ solvatochromism was evaluated using solvents spanning the entire polarity range, from hexane to DMSO. Each compound in the series exhibited positive solvatochromism. The exception was methanol, in which specific solvent–solute interactions led to fluorescence extinction and distinct solvatochromic behaviors in protic and aprotic solvents.^26^ Additionally, novel phenothiazine chalcones exhibit a positive solvatochromism in the presence of solvents with varying polarities. In this series of compounds, electron-donor phenothiazine was bridged with substitutes of varying electron character. The derivatives exhibited a red shift, but the intramolecular charge transfer ability varied. The chalcones with an electron-donating 4-methoxyphenyl ring and a massive 1-naphthyl ring exhibited the most incredible contrast. The first positively affected the phenomenon, while the second harmed intramolecular charge transfer. Despite their advantageous properties, they are promising for bioimaging and optoelectronic applications.^27^ An A-D-A phenothiazine derivative (PTZ-BEI) that exhibited blue-shifted emission spectra as the polarity of the solvent increased was recently synthesized. PTZ-BEI exhibits significant Stokes’ shifts that are solvent-dependent and grow as the polarity of the solvent increases, in addition to the negative solvatochromism. The relative quantum yield demonstrates a similar dependence.^28^ By varying the length of the alkyl chain attached to the phenothiazine ring or its substituents, previous research has shown various functionalization possibilities for phenothiazine. However, there remains much to learn about this topic.^29,30^ It is well established that the length of the alkyl chain influences the physicochemical and photophysical properties of compounds. For example, the alkyl chain length influences solubility, reduces intramolecular aggregation, and inhibits charge recombination.^31–33^ Our previous research demonstrated that the length of the alkyl chain on the carbazole unit affects the photophysical behavior of 2,4-dicarbazolyl-substituted quinolines D–A systems synthesized by the Suzuki–Miyaura cross-coupling reaction, as does the location of the carbazole molecule’s connection to the quinoline core. Observations were made regarding the optical and electrochemical properties of the compounds. Intriguingly, it was discovered that extending the chain to octyl increased the fluorescence capacity of the studied systems, whereas the decyl chain significantly decreased it. However, the newly synthesized and characterized compounds appear to have potential optoelectronic applications.^34^ In addition, research on a series of *N*-alkylated phenothiazine derivatives with a formyl group as a functional group has disclosed significant changes in the properties of tested compounds with only superficial modifications. Changes in chain length affected the solid-state fluorescence performance of the described compounds. The longer alkyl chain produced more remarkable fluorescent contrasts than shorter alkyl chains.^35^

This paper presents a new series of Sonogashira-coupled symmetrical *N*-ethyl-phenothiazine homologues with carbazole groups at the 3 and 7 positions and alkyl chain lengths ranging from C2 to C16 (Figure 1). We have investigated the effect of chain elongation at carbazole substituents on the optical properties of novel phenothiazine derivatives. Their photophysical properties were investigated using experimental techniques and DFT calculations to elucidate structure-property relationships and charge-transfer behavior. Furthermore, we examined their electrochemical profiles and evaluated their potential for use in fluorescence-based bioimaging. Particular attention was given to their behavior in biological environments and their capacity to act as responsive fluorescent probes under conditions that permeabilize membranes.

**Figure 1:**
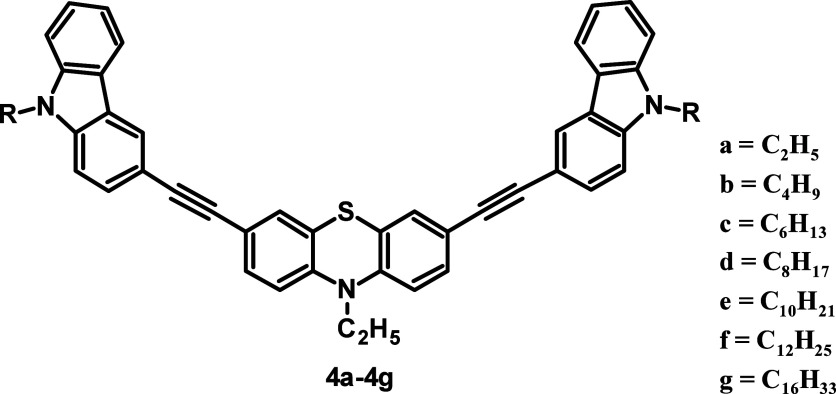
Structure of target compounds **4a**–**4g**.

## 2 Materials and Methods

### 2.1 3,7-Bis­((*N*-alkylcarbaz-3-yl)­ethynyl)-*N*-Ethylphenothiazine Derivatives (**4a–4g**). General Procedure

In 30 mL of tetrahydrofuran, 3,7-bis­(trimethylsilylethynyl)-*N*-ethylphenothiazine (0.5 g, 1.19 mmol) and 3-iodo-*N*-alkylocarbazole (2.8 mmol) were dissolved. The mixture was vigorously stirred under argon within 30 min. Subsequently, a catalytic system of [Pd­(PPh_3_)_4_] (0.15 mmol) and CuI (0.15 mmol) was added, followed by the addition of tetrabutylammonium fluoride (TBAF) (3.57 mmol) via syringe. The resulting suspension was stirred under argon for 24 h at 65 °C. The reaction mixture was cooled to room temperature, concentrated under reduced pressure, and the crude product was purified by column chromatography on silica gel using a mixture of solvents (hexane/DCM).

#### 2.1.1 3,6-Bis­(*N*-ethylcarbaz-3-ylethynyl)-*N*-ethylphenothiazine (**4a**)

Compound **4a** was purified by column chromatography ((hexane/DCM 10:1, 7:1, 3:1 *v*/*v*)) afforded an orange-yellow solid of **4a**. Yield: 36%. ^1^H NMR (400 MHz, CDCl_3_, δ) 8.31 (s, 2H), 8.12 (d, *J* = 7.6 Hz, 2H), 7.64 (d, *J* = 8.4 Hz, 2H), 7.52 (d, *J* = 8.0 Hz, 2H), 7.46 (d, *J* = 8.0 Hz, 2H), 7.39 (m, 4H), 7.31 (m, 2H), 7.13 (dd, *J* = 7.6, 2.4 Hz, 2H), 6.85 (d, *J* = 7.2 Hz, 2H), 4.41 (m, 4H), 3.99 (m, 2H), 0.92 (m, 9H).^13^C NMR (101 MHz, CDCl_3_, δ) 140.35, 139.54, 130.67, 129.98, 129.23, 126.11, 124.40, 123.96, 123.50, 123.00, 122.62, 120.62, 119.36, 118.06, 114.76, 113.40, 108.69, 108.49, 90.84, 86.81, 37.69, 31.53, 30.18, 29.73. Elem. Anal. (%) Calcd for C_46_H_35_N_3_S: C, 83.48; H, 5.33; N, 6.35, found: C, 83.39; H, 5.78; N, 6.20.

#### 2.1.2 3,6-Bis­(*N*-butylcarbaz-3-ylethynyl)-*N*-ethylphenothiazine (**4b**)

Compound **4b** was purified by column chromatography (hexane/DCM 3:1, 2:1, 1:1 *v*/*v*), affording an orange-yellow solid of **4b**. Yield: 39%. ^1^H NMR (400 MHz, CDCl_3_, δ) 8.30 (d, *J* = 1.2 Hz, 2H), 8.12 (d, *J* = 7.6 Hz, 2H), 7.63 (dd, *J* = 8.4, 1.2 Hz, 2H), 7.55–7.46 (m, 2H), 7.44 (d, *J* = 8.4 Hz, 2H), 7.35 (m, 8H), 6.85 (d, *J* = 8.4 Hz, 2H), 4.33 (t, *J* = 7.2 Hz, 4H), 3.97 (q, *J* = 6.8 Hz, 2H), 1.89 (m, 4H), 1.50–1.43 (m, 7H), 1.01–0.95 (m, 6H). ^13^C NMR (101 MHz, CDCl_3_, δ) 140.84, 140.05, 134.26, 130.66, 129.98, 129.17, 126.05, 123.88, 123.62, 122.88, 122.50, 120.53, 119.30, 114.75, 113.34, 110.96, 108.92, 108.73, 90.83, 86.81, 42.98, 42.15, 31.01, 29.68, 20.55, 13.87. Elem. Anal. (%) Calcd for C_50_H_43_N_3_S: C, 83.64; H, 6.04; N, 5.85, found: C, 83.26; H, 6.00; N, 5.41.

#### 2.1.3 3,6-Bis­(*N*-hexylcarbaz-3-ylethynyl)-*N*-ethylphenothiazine (**4c**)

Compound **4c** was purified by column chromatography (hexane/DCM, 10:1, 5:1, 3:1, 1:1, 1:2 *v*/*v*) afforded a yellow oil of **4c**. Yield: 33%. ^1^H NMR (400 MHz, CDCl_3_, δ) 8.30 (s, 2H), 8.12 (d, *J* = 7.6 Hz, 2H), 7.64 (dd, *J* = 8.4, 1.2 Hz, 2H), 7.51 (m, 2H), 7.43 (d, *J* = 8.0 Hz, 2H), 7.40–7.30 (m, 8H), 6.85 (d, *J* = 8.4 Hz, 2H), 4.36–4.27 (t, *J* = 7.2 Hz, 4H), 3.97 (m, 2H), 1.94–1.84 (m, 4H), 1.33 (m, 15H), 0.91 (q, *J* = 6.7 Hz, 6H).^13^C NMR (101 MHz, CDCl_3_, δ) 143.87, 140.85, 140.06, 130.68, 129.98, 129.20, 126.07, 123.89, 123.64, 122.90, 122.53, 120.54, 119.32, 118.10, 114.76, 113.37, 108.93, 108.74, 90.89, 86.85, 43.23, 31.58, 30.21, 29.74, 28.95, 26.97, 22.56, 14.01. Elem. Anal. (%) Calcd for C_54_H_51_N_3_S: C, 83.79; H, 6.64; N, 5.43. Found: C, 83.22; H, 6.42; N, 5.04.

#### 2.1.4 3,6-Bis­(*N*-octylcarbaz-3-ylethynyl)-*N*-ethylphenothiazine (**4d**)

Compound **4d** was purified by column chromatography (hexane/DCM, 2:1, 1:1 *v*/*v*) afforded an orange oil of **4d**. Yield: 34%. ^1^H NMR (400 MHz, CDCl_3_, δ) 8.30 (s, 2H), 8.13 (d, *J* = 7.6 Hz, 2H), 7.64 (dd, *J* = 8.4, 1.2 Hz, 2H), 7.52 (m, 2H), 7.43 (d, *J* = 8.0 Hz, 2H), 7.41–7.29 (m, 8H), 6.85 (d, *J* = 8.4 Hz, 2H), 4.31 (t, *J* = 7.2 Hz, 4H), 3.97 (m, 2H), 1.93–1.85 (m, 4H), 1.51–1.44 (m, 4H), 1.43–1.24 (m, 15H), 0.91 (m, 10H). ^13^C NMR (101 MHz, CDCl_3_, δ) 140.84, 140.07, 130.65, 129.95, 129.86, 129.80, 129.22, 126.06, 124.00, 123.91, 122.89, 122.53, 120.54, 119.31, 114.80, 113.33, 108.93, 108.73, 90.94, 86.64, 43.23, 31.80, 30.20, 29.73, 29.37, 29.18, 28.98, 27.31, 22.62, 14.08. Elem. Anal. (%) Calcd for C_58_H_59_N_3_S: C, 83.91; H, 7.16; N, 5.06. Found: C, 83.26; H, 6.81; N, 4.67.

#### 2.1.5 3,6-Bis­(*N*-decylcarbaz-3-ylethynyl)-*N*-ethylphenothiazine (**4e**)

Compound **4e** was purified by column chromatography (hexane/DCM, 5:1, 3:1, 1:1 *v*/*v*) afforded an orange oil of **4e**. Yield: 37%. ^1^H NMR (400 MHz, CDCl_3_, δ) 8.30 (s, 2H), 8.12 (d, *J* = 7.6 Hz, 2H), 7.64 (dd, *J* = 8.4, 1.2 Hz, 2H), 7.51 (m, 2H), 7.43 (d, *J* = 8.0 Hz, 2H), 7.37 (m, 8H), 6.85 (d, *J* = 8.4 Hz, 2H), 4.31 (t, *J* = 7.2 Hz, 4H), 3.97 (q, *J* = 6.7 Hz, 2H), 1.89 (m, 4H), 1.51–1.44 (m, 4H), 1.31 (m, 23H), 0.94–0.86 (m, 10H).^13^C NMR (101 MHz, CDCl_3_, δ) 143.86, 140.83, 140.05, 130.68, 129.98, 129.20, 126.08, 123.89, 123.60, 122.89, 122.52, 120.55, 119.32, 118.08, 114.76, 113.35, 108.95, 108.76, 90.90, 86.86, 43.22, 42.13, 31.89, 29.56, 29.54, 29.42, 29.30, 28.99, 27.32, 22.71, 14.16, 12.88. Elem. Anal. (%) Calcd for C_63_H_71_N_3_S: C, 84.02; H, 7.62; N, 4.74. Found: C, 84.21; H, 7.57; N, 4.43.

#### 2.1.6 3,6-Bis­(*N*-dodecylcarbaz-3-ylethynyl)-*N*-ethylphenothiazine (**4f**)

Compound **4f** was purified by column chromatography (hexane/DCM, 10:1, 8:1, 5:1, 2:1, 1:1 *v*/*v*) afforded a dark orange oil of **4f**. Yield: 31%. ^1^H NMR (400 MHz, CDCl_3_, δ) 8.30 (s, 2H), 8.12 (d, *J* = 7.6 Hz, 2H), 7.63 (d, *J* = 8.4 Hz, 2H), 7.51 (m, 2H), 7.43 (d, *J* = 8.0 Hz, 2H), 7.42–7.29 (m, 8H), 6.85 (d, *J* = 8.4 Hz, 2H), 4.32 (t, *J* = 7.2 Hz, 4H), 3.98 (m, 2H), 1.95–1.80 (m, 4H), 1.48 (t, *J* = 6.9 Hz, 4H), 1.35–1.22 (m, 31H), 0.90 (m, 10H). ^13^C NMR (101 MHz, CDCl_3_, δ) 143.87, 140.86, 140.07, 130.69, 129.99, 129.23, 126.08, 124.43, 123.90, 123.53, 122.93, 122.56, 120.55, 119.34, 114.77, 113.42, 108.95, 108.75, 90.94, 86.88, 43.23, 35.04, 34.49, 31.97, 31.57, 30.23, 29.77, 29.66, 29.62, 29.55, 29.43, 29.39, 29.00, 27.33, 22.75, 14.17. Elem. Anal. (%) Calcd for C_66_H_75_N_3_S: C, 84.12; H, 8.02; N, 4.46. Found: C, 83.99; H, 8.45; N, 4.86.

#### 2.1.7 3,6-Bis­(*N*-hexadecylcarbaz-3-ylethynyl)-*N*-ethylphenothiazine (**4g**)

Compound **4g** was purified by column chromatography (hexane/DCM, 8:1, 5:1, 2:1, 1:1 *v*/*v*), affording a yellow solid of **4g**. Yield: 39%. ^1^H NMR (500 MHz, CDCl_3_, δ) 8.32 (s, 2H), 8.14 (d, *J* = 7.6 Hz, 2H), 7.65 (dd, *J* = 8.4, 1.2 Hz, 2H), 7.55–7.49 (m, 2H), 7.44–7.34 (m, 8H), 6.84 (m, 2H), 4.22–4.15 (m, 4H), 4.02–3.94 (m, 2H), 1.39–1.30 (m, 45H), 1.00–0.88 (m, 20H).^13^C NMR (101 MHz, CDCl_3_, δ) 143.88, 141.32, 140.56, 130.69, 129.99, 129.18, 126.05, 124.42, 123.84, 123.53, 122.89, 122.52, 120.49, 119.33, 114.77, 113.39, 109.24, 109.04, 90.90, 86.90, 47.56, 39.42, 35.04, 34.49, 31.99, 31.57, 31.07, 30.23, 29.76, 29.72, 29.42, 28.83, 24.46, 23.07, 22.75, 14.17, 14.04, 10.94. Elem. Anal. (%) Calcd for C_74_H_91_N_3_S: C, 84.28; H, 8.70; N, 3.98, found: C, 84.48; H, 8.42; N, 3.91.

## 3 Results and Discussion

### 3.1 Synthesis and Characterization

Scheme 1 outlines the synthetic route of novel symmetrically substituted phenothiazine derivatives **4a**–**4g**.

**Scheme 1:**
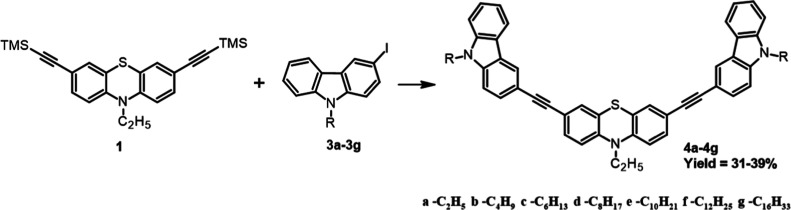
Synthetic Route for the Preparation of **4a**–**4g**. Reagents and conditions: TBAF, THF, 2.4 eq of **3a**–**3g**, [Pd­(PPh_3_)_4_]/CuI, 65 °C, Ar, 24 h

The key precursor, 3,7-bis (trimethylsilylethynyl)-*N*-ethylphenothiazine, was synthesized according to well-established procedures.^36–38^ The 3-iodo-*N*-alkylcarbazole intermediates (**3a**–**3g**) were obtained via a two-step synthesis starting from commercially available carbazole, affording the products in 35–85% yields.^39^ The final cross-coupling reaction involved in situ desilylation of 3,7-bis­(trimethylsilylethynyl)-*N*-ethylphenothiazine (**1**) with tetrabutylammonium fluoride (TBAF), followed by Sonogashira coupling reaction with the appropriate 3-iodo-*N*-alkylcarbazole derivatives **3a**–**3g** in the presence of a palladium/copper catalytic system (Scheme 1). A moderate yield of 31–39% was obtained for seven new symmetrically substituted *N*-ethyl phenothiazine derivatives (**4a**–**4g**). ^1^H and ^13^C NMR, as well as elemental analysis, were used to characterize the novel phenothiazine derivatives (Supporting Information, Figures S16–S29).

### 3.2 DFT Calculations

To understand the nature of the new symmetrical phenothiazine-based system **4a**–**4g**, quantum chemical calculations were performed using DFT method, as implemented in the Gaussian 16 package.^40^ All calculations were performed in the polarizable continuum model (PCM).^41^ All orbitals were computed at an isovalue of 0.03 e/bohr.^3^ The optimized geometric parameters at the B3LYP/6–311 + G­(d,p) level, along with the frontier molecular electron density contours for **4a**–**4g**, are displayed in Table 1. A clear evolution of orbital contributions can be observed across the series **4a**-**4g**, for HOMO–1, the carbazole unit consistently provides the dominant contribution, however, its participation decreases from 58% in **4a** to 43–49% in **4b**–**4g**, accompanied by a slight increase in the phenothiazine contribution from 32% to 35–38%. Simultaneously, the acetylene linker contribution is the highest for **4b** (10%) and lower and nearly constant for the remaining derivatives (7%), indicating a subtle redistribution of orbital density induced by substitution pattern. In the case of the HOMO, phenothiazine is the principal contributor for all compounds, yet a gradual decrease is observed from 67% in **4a** to 59–63% in **4b**–**4g**, while the carbazole contribution shows a complementary increase from 27% to 28–33%. The acetylene linker contribution remains low but exhibits a small maximum for **4a**–**4c** (6%), decreasing to 5% for some of the later derivatives. For the LUMO, phenothiazine dominance is most pronounced and relatively invariant across the series (80–84%), whereas carbazole contributions remain minor (11–16%) and show no systematic trend. The acetylene linker contribution is minimal and uniform (3–4%), confirming the strong localization of the lowest unoccupied orbital on the phenothiazine core regardless of substitution. More pronounced variations are observed for LUMO + 1, where phenothiazine contributions decrease from 57% in **4a** to 43–45% in **4b**–**4d**, followed by stabilization at 43–44% in **4e**–**4g**. This reduction is compensated by an increased carbazole contribution, reaching 38% in **4a** and stabilizing around 29% for **4c**–**4g**. Notably, compound **4b** exhibits a markedly higher contribution from the alkyl substituents (27%) compared to the remaining derivatives (24–25%), indicating enhanced participation of peripheral groups in higher unoccupied orbitals. The acetylene linker contribution to LUMO + 1 shows a gradual decrease along the series, from 5% in **4a** to 2–3% in **4c**–**4g**. Different alkyl substituents do not affect the HOMO–LUMO energy gap, which remains constant at 3.58 eV for all compounds **4a**–**4g** (see Table 2).

**Table 1:**
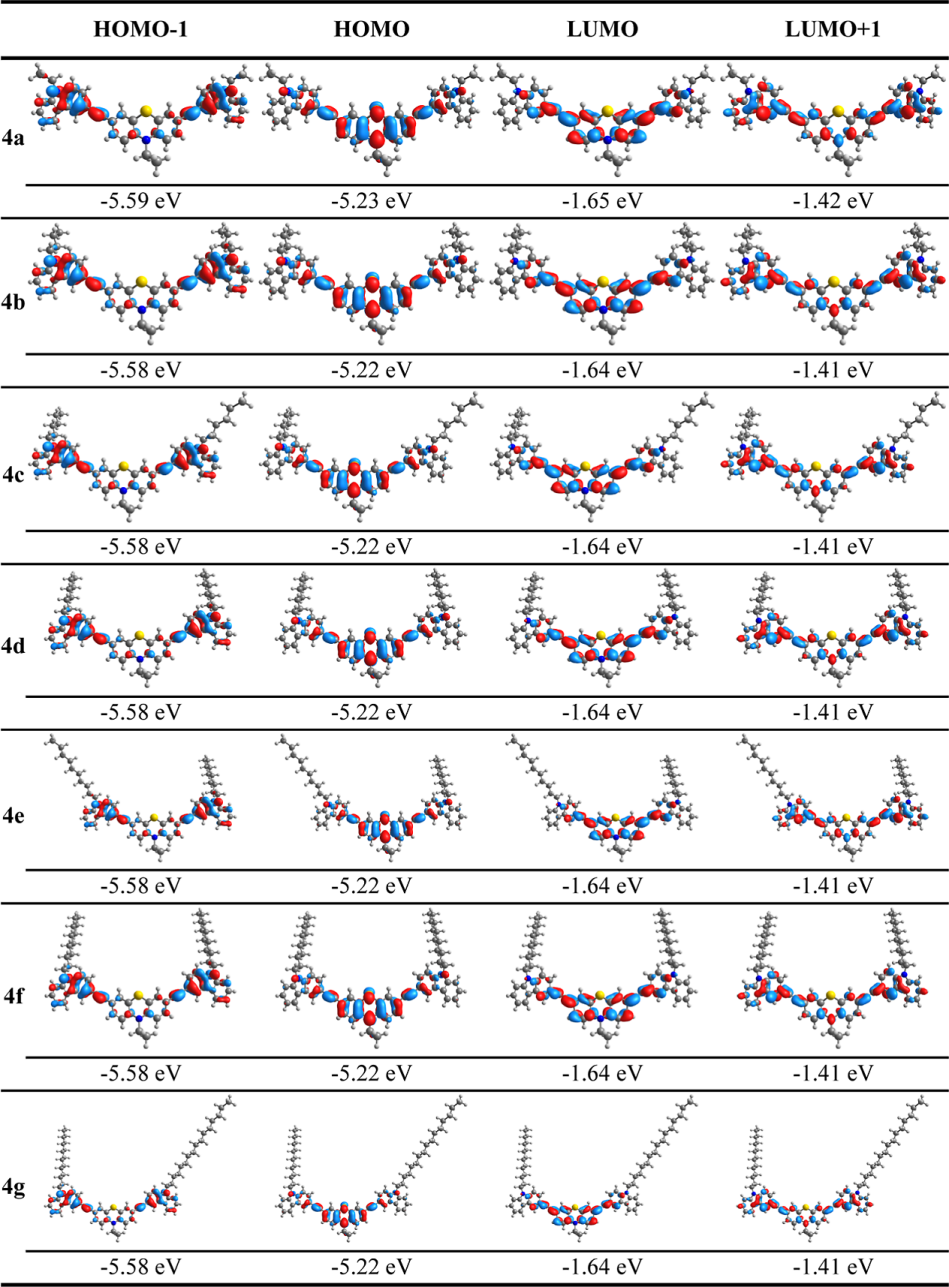
Optimized Structures B3LYP/6-311+G­(d,p) in Dichloromethane with Contours of Selected Orbitals (HOMO–1, HOMO, LUMO, LUMO + 1) and Their Energies for **4a**–**4g**

**Table 2:**
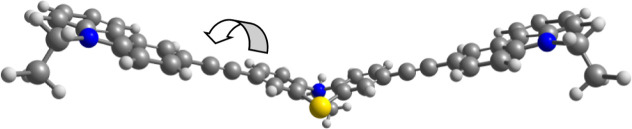
Calculated (B3LYP/6-311+G­(d,p), DCM) Angles between Carbazole and Phenothiazine

	ground state *S*_0_ [°]	Δ [°]	average [°]	excited state *T*_1_ [°]	Δ [°]	average [°]	excited state *S*_1_ [°]	Δ [°]	average [°]	Δ (*T*_1_–*S*_0_) [°]	Δ (*S*_1_– *S*_0_) [°]	Δ (*T*_1_–*S*_0_) av [°]	Δ (*S*_1_–*S*_0_) av [°]
**4a**	1.39/1.19	0.20	1.29	1.05/0.92	0.13	0.99	0.91/0.91	0.00	0.91	–0.07	–0.20	–0.30	–0.38
**4b**	1.38/1.33	0.05	1.36	0.83/0.72	0.11	0.78	0.54/0.54	0.00	0.54	0.06	–0.05	–0.58	–0.82
**4c**	3.72/0.54	3.18	2.13	0.65/0.23	0.42	0.44	0.57/0.52	0.05	0.55	–2.76	–3.13	–1.69	–1.58
**4d**	1.85/1.68	0.17	1.77	1.13/0.85	0.28	0.99	0.99/0.99	0.00	0.99	0.11	–0.17	–0.78	–0.78
**4e**	2.81/0.53	2.28	1.67	1.15/0.63	0.52	0.89	1.66/1.12	0.54	1.39	–1.76	–1.74	–0.78	–0.28
**4f**	2.46/2.38	0.08	2.42	0.77/0.50	0.27	0.61	0.61/0.61	0.00	0.61	0.19	–0.08	–1.81	–1.81
**4g**	2.27/1.13	1.14	1.70	0.90/0.64	0.26	0.77	1.01/0.31	0.70	0.66	–0.88	–0.44	–0.93	–1.04

To further elucidate the structure-property relationships in compounds **4a**–**4g**, geometry optimizations of the lowest singlet (*S*_1_) and triplet (*T*_1_) excited states were performed. The analysis focused on two key geometric descriptors: the butterfly angle of the phenothiazine core and the dihedral angles between phenothiazine and the terminal carbazole units (Tables S1, S2, and 2). In the ground state (*S*_0_), the phenothiazine butterfly angle remains essentially invariant across the entire series (138.44–138.74°), demonstrating that variation of the alkyl substituents does not influence the folded geometry of the core. Upon excitation, all compounds undergo pronounced planarization, with butterfly angles increasing to 167.65–168.32° in *T*_1_ and 166.33–166.92° in *S*_1_. The magnitude of this structural reorganization is comparable for all derivatives, with Δ­(*T*_1_–*S*_0_) values of approximately 29–30° and Δ­(*S*_1_–*S*_0_) values of 27–28°, indicating that excitation-induced flattening of phenothiazine is a general feature of the series and largely independent of substitution pattern. More distinct trends are observed for the dihedral angles between phenothiazine and the carbazole units. In *S*_0_, all compounds display small torsion angles, but their average values increase from 1.29° for **4a** to 2.42° for **4f**, revealing a gradual rise in ground-state conformational flexibility with increasing alkyl chain length. In the ground state (*S*_0_), several derivatives, most notably **4c** and **4e**, exhibit pronounced intramolecular asymmetry, with differences between the two carbazole–phenothiazine dihedral angles exceeding 2°, despite chemical symmetry. Upon excitation to the *T*_1_ state, a general decrease in the average dihedral angles is observed for all compounds, indicating increased coplanarity of the D−π–PTZ−π–D framework. The extent of this planarization varies across the series and is most pronounced for **4f** and **4c**, which display the largest average dihedral-angle reductions (Δ­(*T*_1_–*S*_0_) = −1.81° and −1.69°, respectively). Compound **4b** shows a moderate decrease in the average dihedral angle (−0.58°), whereas **4a** and **4d** exhibit comparatively smaller changes. Notably, excitation often enhances arm-to-arm asymmetry, indicating nonequivalent geometric relaxation of the two carbazole units in the triplet state.

A consistent increase in asymmetry between the two donor arms is observed upon excitation, particularly in the *T*_1_ state. For example, in **4g**, one carbazole unit becomes nearly coplanar with the phenothiazine core, while the second remains more twisted, highlighting nonequivalent electronic relaxation pathways within a single molecule. Similar, though less pronounced, effects are evident for **4c** and **4e**, where excitation reduces but does not eliminate the initial asymmetry. The optimized *S*_1_ geometries exhibit considerably smaller deviations from *S*_0_ than those observed for *T*_1_, with average dihedral changes typically below 1°. This indicates that the singlet excited state largely preserves the ground-state geometry, whereas the triplet state undergoes substantially deeper conformational relaxation. Analysis of the frontier molecular orbitals shows that the *S*_0_ and *S*_1_ states are electronically similar, whereas the *T*_1_ state exhibits noticeable redistribution of orbital overlap (Table S2). Despite these geometric and electronic differences, the HOMO and LUMO energies of *S*_0_, *S*_1_, and *T*_1_ remain nearly identical for all compounds (Table S3), and the calculated Δ*E*(*T*_1_–*S*_1_) values are narrowly distributed (2653–2655 cm^–1^).

### 3.3 Photophysical Properties

UV–vis absorption and fluorescence (PL) spectra of the novel compounds were obtained to investigate, in depth, the effect of alkyl chain length on the optical behavior of the novel symmetrical phenothiazine derivatives **4a**–**4g**. Intentionally symmetrically substituted phenothiazine derivatives were designed to avoid the influence of the additional substituents and symmetry disturbance on the photophysical properties. The absorption and PL studies were conducted in various solvents of different polarities (hexane, toluene, dichloromethane, methanol, and acetonitrile) and at low temperatures in a methanol/ethanol (4:1) solution at 77 K. The absorption spectra of exemplary compounds **4a** and **4e** are illustrated in Figure 2, and the other in Supporting Information (Figure S2), and the optical characteristics of all compounds are summarized in Table 3. The absorption range and peak positions are approximately the same for all novel compounds **4a**–**4g** (Figure 2) in the respective solvents. Compounds **4a**–**4g** display two or three distinct absorption bands in the 250–450 nm range in all examined solvents. The absorption bands at 250–340 nm result from the delocalization of electrons in carbazole and phenothiazine over the molecules and are attributed to π–π* electronic transitions. The broad absorption band between 340 and 450 nm can be attributed to intramolecular charge transfer (ICT) from the electron-rich phenothiazine unit to the electron-less donating acetylene linker and carbazole moieties (Table 1). These indicate that the variable length of the alkyl chain at carbazole units does not affect the position of maxima absorption of **4a**–**4g** molecules, and their ground state is relatively nonpolar and devoid of substantial solvent and substituent effects.

**Figure 2:**
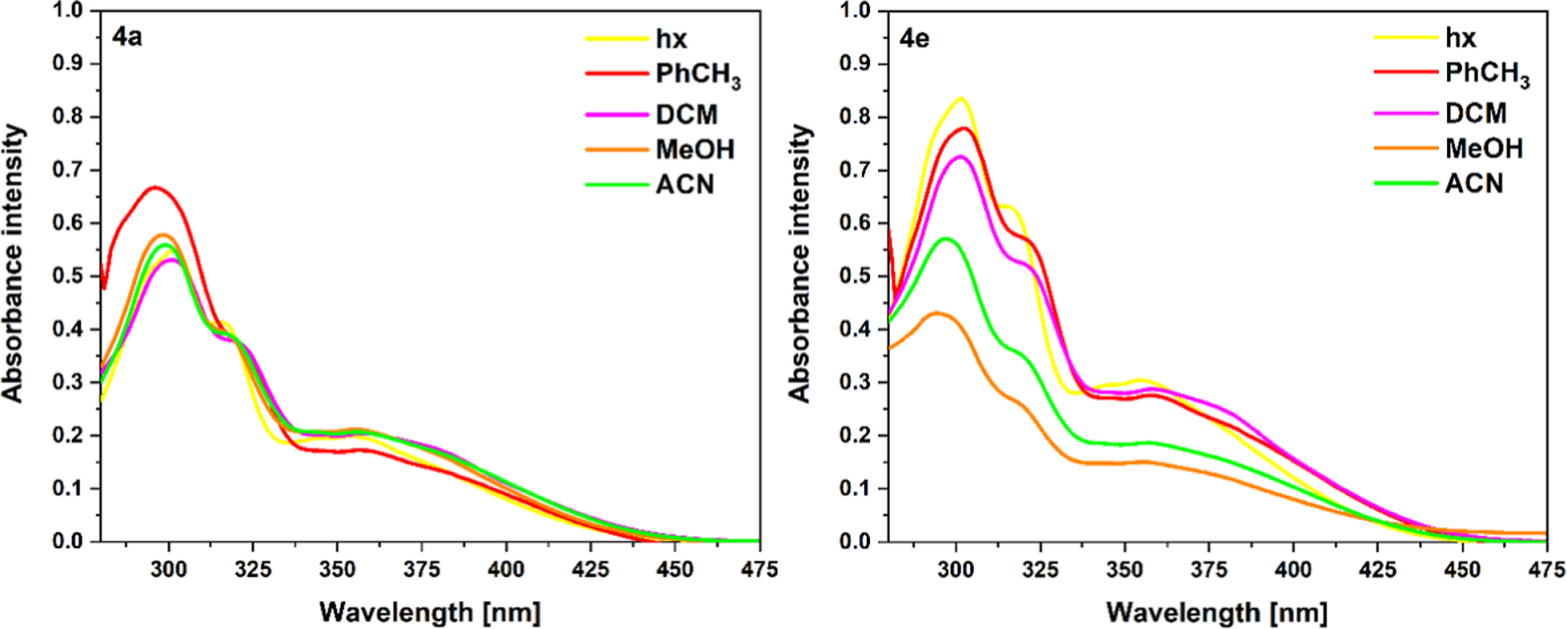
UV–vis absorption spectra of **4a** and **4e** in different solvents (*c* = 1.0 × 10^–5^ M).

**Table 3:** Photophysical Data for **4a**–**4g** Recorded in Different Solvents

solvent	λ_abs_ [nm]						
	4a	4b	4c	4d	4e	4f	4g
**hx**	301 (0.55), 315 (0.41), 356 (0.21)	302 (0.90), 316 (0.72), 355 (0.39)	293 (1.36), 317 (0.70), 357 (0.29)	301 (0.95), 316 (0.70), 354 (0.35)	302 (0.84), 317 (0.63), 355 (0.31)	302 (0.69), 316 (0.52), 354 (0.25)	301 (0.69), 318 (0.52), 354 (0.25)
**PhCH**_3_	296 (0.67), 322 (0.37), 357 (0.17)	294 (1.01), 322 (0.55), 358 (0.26)	302 (0.73), 322 (0.53), 358 (0.25)	301 (0.86), 321 (0.63), 356 (0.30)	302 (0.78), 322 (0.56), 358 (0.28)	300 (0.56), 321 (0.46), 358 (0.22)	300 (0.68), 321 (0.46), 359 (0.22)
**DCM**	301 (0.53), 320 (0.38), 357 (0.21)	302 (0.80), 322 (0.57), 358 (0.32)	299 (0.83), 321 (0.54), 359 (0.29)	301 (0.93), 320 (0.67), 358 (0.36)	302 (0.73), 321 (0.52), 358 (0.29)	300 (0.59), 322 (0.39). 362 (0.23)	299 (0.72), 320 (0.47), 359 (0.25)
**MeOH**	298 (0.58), 316 (0.40), 355 (0.21)	294 (0.98), 317 (0.57), 356 (0.31)	297 (0.64), 317 (0.41), 357 (0.22)	297 (0.64), 318 (0.43), 355 (0.25)	294 (0.43), 318 (0.26), 355 (0.16)	294 (0.33), 319 (0.20), 356 (0.12)	295 (0.76), 319 (0.46), 355 (0.26)
**ACN**	299 (0.56), 318 (0.39), 359 (0.21)	299 (0.84), 318 (0.58), 358 (0.31)	298 (0.83), 318 (0.56), 358 (0.29)	299 (0.63), 320 (0.36), 357 (0.20)	297 (0.67), 319 (0.35), 358 (0.19)	293 (0.85), 320 (0.41), 357 (0.22)	297 (0.73), 319 (0.47), 356 (0.25)

In contrast, although the peaks and positions of the absorption curves for **4a**–**4g** are essentially identical, the absorption coefficient within each solvent varies significantly (Table 3). In each solvent, the absorption coefficient at concentration 1.0 × 10^–5^ M of the lowest energy band is the smallest for compounds **4a** and **4f** and the highest for compounds **4b** and **4c**. A similar trend is observed for the absorption coefficients of the most energetic transitions. Still, it is more pronounced for compound **4a** with the shortest alkyl chain at the carbazole units, where its absorption coefficient is almost twice as small as the others. This phenomenon indicates that the longer alkyl chains at carbazole units, although they do not modify the energy of particular orbitals, can slightly increase conjugation through steric arrangement and enhanced coplanarity, increasing in absorption intensity. Moreover, the participation of the alkyl chain in the creation of HOMO orbitals for compounds **4b**–**4g** is discernible in opposition to the small methyl units of compound **4a** (Figure S1). On the other hand, a pretty high absorption coefficient of compounds indicates the differences in charge distributions due to stronger intermolecular interactions in polar and nonpolar solvents in the excited state of the compounds.^42^

The examined emission spectra of compounds **4a**–**4g** in solvents with diverse polarities exhibited moderate solvent-dependent wavelength shifts and strong intensity characteristics (Figures 3 and S3). While the absorption spectra of all compounds remain largely unaffected by solvent polarity, showing only minor variations in band maxima, emission spectra are more sensitive and reveal solvent-dependent relaxation and reorganization effects in the excited state. The spectra displayed a single peak with a shoulder in all solvents, exhibiting a distinct bathochromic shift with increasing solvent polarity, indicative of positive solvatochromism. Emission maxima range from ∼462 nm in hexane to ∼480 nm in acetonitrile, with substantial Stokes shifts (e.g., 6600–8400 cm^–1^) (Table 4).

**Figure 3:**
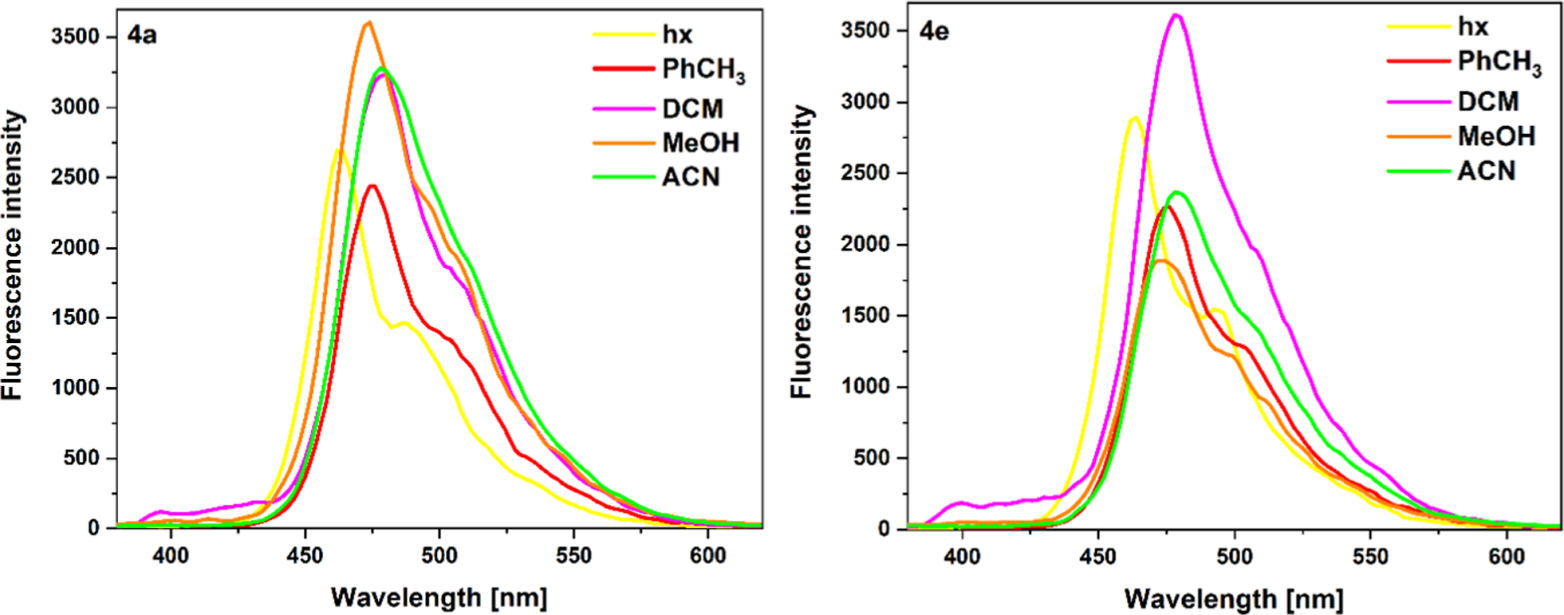
Fluorescence spectra of **4a** and **4e** in different solvents (*c* = 1.0 × 10^–5^ M).

**Table 4:** Photophysical Data for **4a**–**4g** Recorded in Different Solvents

solvent	PL λ_em_ [nm]/stokes shift[cm^–1^]						
	4a	4b	4c	4d	4e	4f	4g
**hx**	462/6445, 486/7515	464/6617, 494/7926	462/6366, 486/7435	466/6789, 490/7840	462/6524, 494/7926	464/6697, 488/7757	466/6789, 494/8006
**PhCH**_3_	476/7003, 502^sh^/8091	476/6925, 502^sh^/8013	476/6925, 506^sh^/8170	476/7081, 504^sh^/8249	476/6925, 502^sh^/8013	476/6925, 504^sh^/8092	476/6847, 508^sh^/8170
**DCM**	480/7178	478/7012, 504^sh^/8092	478/6935, 508^sh^/8170	478/7012, 506^sh^/8170	478/7012, 506^sh^/8170	480/6791, 500^sh^/7624	478/6935, 508^sh^/8170
**MeOH**	474/7072, 498^sh^/8089	474/6993, 502^sh^/8170	474/6914, 500^sh^/8111	472/6983, 504^sh^/8328	474/7072, 499^sh^/8129	472/6903, 502^sh^/8170	474/7072, 502^sh^/8249
**ACN**	478/6935	480/7100	478/7012, 506^sh^/8170	480/7178, 510^sh^/8403	480/7100, 508^sh^/8248	480/7178, 506^sh^/8248	480/7257

Among the studied compounds, derivatives with medium-length alkyl chains, **4b** (butyl) and **4c** (hexyl), and the longest alkyl chain, **4g** (hexadecyl), exhibit the most favorable photophysical properties. The compounds achieve fluorescence quantum yields of 81% (**4b**), 76% (**4c**), and 70% (**4g**) in dichloromethane and maintain consistently high efficiencies across all tested solvents (Table 5). These features are attributed to enhanced conjugation and reduced aggregation, driven by optimal steric stabilization, which promotes a more planar molecular geometry favorable to ICT (Tables S1 and S2). In contrast, compound **4a** (with the shortest methyl chains) exhibits lower quantum yield values (48–65%) across the tested solvents, likely due to increased intermolecular interactions and conformational flexibility, which facilitate nonradiative decay pathways. Conversely, compounds **4e** and **4f** exhibited slightly reduced efficiencies and absorption coefficients in most solvents, potentially due to aggregation effects or dynamic disorder at the excited state. The observed chain-length-dependent trend indicates that moderate rigidity in fluorophores suppresses nonradiative processes and enhances the quantum yield. Additionally, a triple bond in the linker unit is preferred, as it provides a coplanar structure and increased stability.^43,44^

**Table 5:** Photophysical Data for **4a**–**4g** Recorded in Different Solvents

solvent	Φ [%]						
	**4a**	**4b**	**4c**	**4d**	**4e**	**4f**	**4g**
**hx**	48	51	51	45	51	51	50
**PhCH**_3_	65	71	71	69	64	65	77
**DCM**	61	81	76	67	68	71	70
**MeOH**	64	78	72	72	63	60	65
**ACN**	60	69	69	69	60	70	68

The fluorescence lifetimes of **4a**–**4g** further confirm the impact of alkyl chain length and solvent polarity (Table S4). All compounds show mono- and biexponential decay curves, with a pronounced contribution of long-lived components in polar solvents (Figures S4–S10). The effective lifetimes ranged from 2.7 to 3.4 ns, with the longest values recorded for **4b** and **4c**. The decay curve of **4a**–**4g** is monoexponentially fitted in hexane and ACN and considerably longer in polar (ACN, τ_eff_ ≈3.3 ns) than in nonpolar solvent (hexane, τ_eff_ ≈2.2 ns), emphasizing the role of solvent polarity in stabilizing the excited state. In contrast, the decay curves of **4a**–**4g** in DCM and MeOH become biexponentially fitted, with the second decay component affecting the efficient lifetime value by approximately 20%, indicating that more than one emissive relaxation pathway is operative under these conditions. This behavior may arise from solvent reorganization processes and conformational subpopulations in polar environments. The biexponential behavior is most likely attributed to solvent-dependent excited-state heterogeneity. In particular, polar and hydrogen-bonding solvents, such as DCM and MeOH, can promote slow solvent reorganization around the excited molecule, leading to the coexistence of initially unrelaxed and solvent-stabilized excited states with different decay rates. Furthermore, the presence of conformational subpopulations, such as different solvation shells or locally stabilized conformers, can lead to the formation of parallel emissive species that decay independently. This highlights the increased complexity of excited-state dynamics in polar media and highlights the sensitivity of compounds **4a**–**4g** to solvent–solute interactions. These lifetime values of **4a**–**4g** are consistent with those reported for symmetrical naphthalimide phenothiazine-based systems, where the compound with a longer alkyl chain at the naphthalimide unit exhibited a lower lifetime of 1.24 ns. In comparison, a structurally similar compound with a shorter (by two carbons) chain displayed a significantly extended lifetime of 3.50 ns.^45^ This trend highlights the importance of symmetric substitution and the enhanced stabilization of the ICT state in modulating excited-state deactivation. The observed fluorescence lifetimes across diverse solvents support radiative decay as the principal deactivation pathway, while maintaining strong emission in all derivatives. Among the investigated compounds, **4b** appears to be the most interesting, exhibiting photophysical properties that clearly distinguish it from the remaining derivatives.

The high values of the Stokes shift (6600–8400 cm^–1^) and bathochromic shift in the range of solvents indicate an ICT process, which should be confirmed by the calculated dipole moment difference between the ground and excited states and Lippert-Mataga plots. Clear trends emerge from the analysis of the ground- and excited-state dipole moments of compounds **4a**–**4g** (Table S5). For all compounds and solvents, the excited-state dipole moments (μ_e_) are consistently lower than the corresponding ground-state values (μ_g_), resulting in negative Δμ values throughout the series. The magnitude of this decrease varies among the derivatives, with the smallest changes observed for **4a** and more pronounced reductions found for **4b**–**4d** and **4f**, the latter showing the most significant decrease in μ_e_ relative to μ_g_. Compounds **4e** and **4g** display intermediate behavior, indicating that the extent of dipole-moment reduction does not follow a strictly monotonic trend with substitution. A clear solvent-dependent trend is observed for all compounds, with both μ_g_ and μ_e_ increasing moderately with solvent polarity in the order hexane < toluene < dichloromethane < methanol ≈ acetonitrile. Importantly, for each compound, the relative difference between μg and μe remains similar across solvents, demonstrating that solvent polarity stabilizes the ground and excited states to a comparable extent. Overall, these trends show that excitation of compounds **4a**–**4g** does not lead to an increase in dipole magnitude, as would be expected for classical intramolecular charge-transfer systems. Instead, the excited-state behavior is characterized by a moderate reduction in dipole moment combined with substantial reorientation of the dipole vector (Table 6), consistent with internal charge redistribution rather than the formation of a strongly polarized excited state.

**Table 6:**
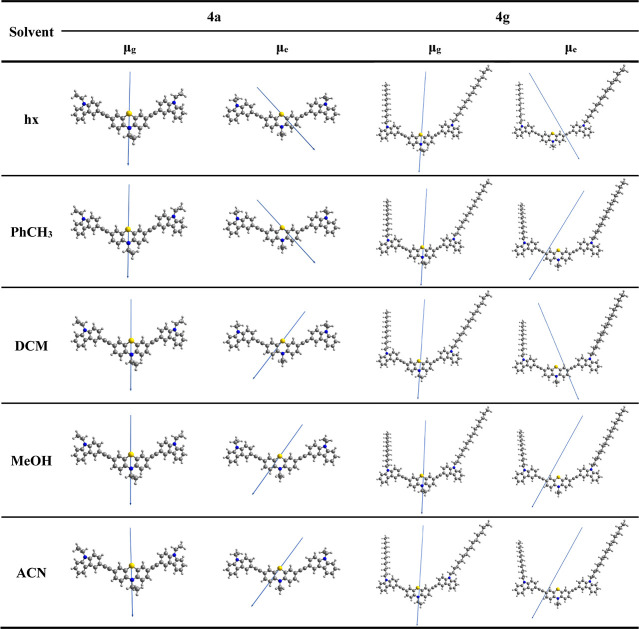
Dipole Moments of μ_g_ and the μ_e_ of **4a** and **4g** in Various Solvents, B3LYP/6-311+G­(d,p)

The physical origin of the dipole-moment direction change was analyzed using electron density difference (Δρ) distributions obtained from grid-based charge-transfer analysis calculated in DCM at the B3LYP/6–311 + G­(d,p) level by using the Multiwfn 3.8 software^46^ as presented in Tables 7 and S6. Green and blue areas indicate regions of electron density gain and loss, respectively, with excitation leading to electron accumulation in the green regions and depletion in the blue ones. For compounds **4a**–**4f**, the separation between the barycenters of positive and negative Δρ regions is slight (*D* = 0.061–0.264 Å), while both regions are spatially extended, as reflected by large Root Mean Square Deviation (RMSD) values (≈9–13 Å) and high overlap integrals (S ± = 0.90–0.99). This indicates that the dominant contribution to the dipole moment originates from anisotropic redistribution of electron density rather than from long-range charge separation. The Cartesian components of the dipole moment variation correlate with the orientation of the Δρ distribution: systems with comparable CT distances but differently oriented Δρ lobes exhibit markedly different dipole directions. Electron density difference maps directly visualize this effect, showing rotation of positive and negative density regions within the molecular framework rather than increased centroid separation. Consequently, the dipole response is governed primarily by the direction of charge redistribution, not by its magnitude. Molecular symmetry further constrains dipole formation by restricting the allowed dipole components, while substituent-induced symmetry lowering relaxes these constraints and enables dipole reorientation without a substantial increase in CT distance. Compound **4g** represents a distinct case, in which a larger CT distance (*D* = 0.664 Å) contributes alongside anisotropic redistribution, resulting in a substantially enhanced dipole magnitude.

**Table 7:**
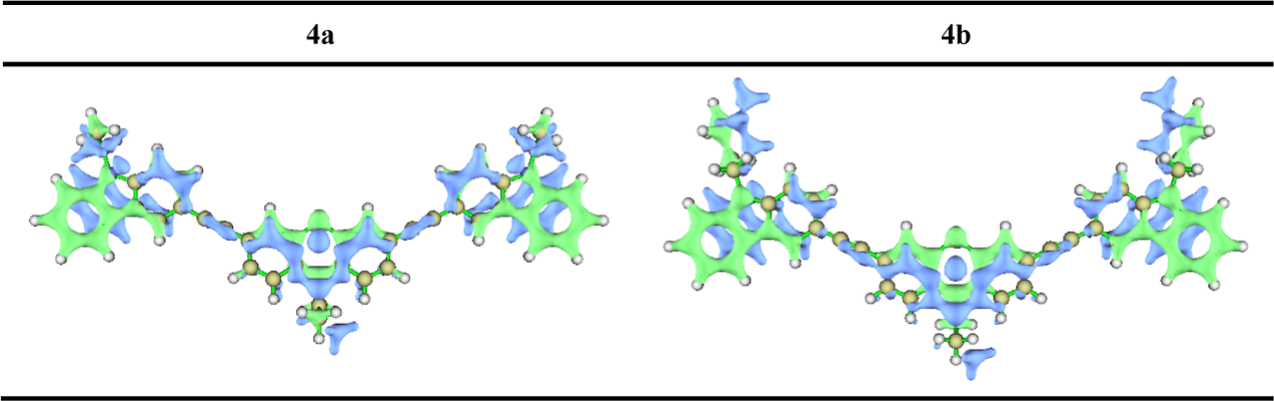
Electron Density Difference (Δρ) Maps for Compounds **4a** and **4b**

The impact of low temperature (77 K) on the optical characteristics was also explored. The summarized data are presented in Table 8, and Figure 4 displays the excitation and emission spectra. The spectra of **4a**–**4g** in the MeOH/EtOH (4:1) mixture at low temperature exhibit two emission maxima at 460 and 490 nm, similar to those measured at room temperature. A broad band at about 550 nm was observed, indicating phosphorescence with a very low band gap of approximately 2.34 eV. This suggests the presence of both fluorescence and possibly phosphorescence or thermally activated delayed fluorescence (TADF), enabled by restricted molecular motion at low temperatures.

**Table 8:** Photophysical Data for **4a**–**4g** Recorded in MeOH/EtOH (4:1, *v*/*v*) at 77 K^a^

	λ_exc_ [nm]	λ_em_ [nm]/stokes shift [cm^–1^]	τ_eff_ [ns]	χ^2^	*E*_g opt_ [eV]
**4a**	304, 324	458/9030, 538/12277	2.23 [0.67 (22.58%), 2.68 (77.42%)]	1.180	2.71/2.31
**4b**	305, 328	460/8749, 488/9996, 531/11655	2.45 [1.06 (24.50%), 2.90 (75.50%)]	1.172	2.70/2.34
**4c**	305, 328	462/8843, 496/10327, 538/11900	2.30 [0.73 (24.37%), 2.81 (75.63%)]	1.078	2.69/2.31
**4d**	307, 329	460/8656, 490/9987, 542/11945	2.07 [0.24 (17.55%), 2.46 (82.45%)]	1.092	2.70/2.29
**4e**	306, 327	462/8936, 492/10256, 550/12399	2.41 [0.68 (16.68%), 2.76 (83.32%)]	1.151	2.69/2.26
**4f**	305, 326	462/9030, 492/10350	2.50 [0.72 (19.64%), 2.93 (80.36%)]	1.049	2.69/2.52
**4g**	305, 331	462/8566, 486/9635, 530/11344	2.48 [0.95 (26.11%), 3.02 (73.89%)]	1.101	2.69/2.34

a *E*_g opt_ = 1241/λ_em_.

**Figure 4:**
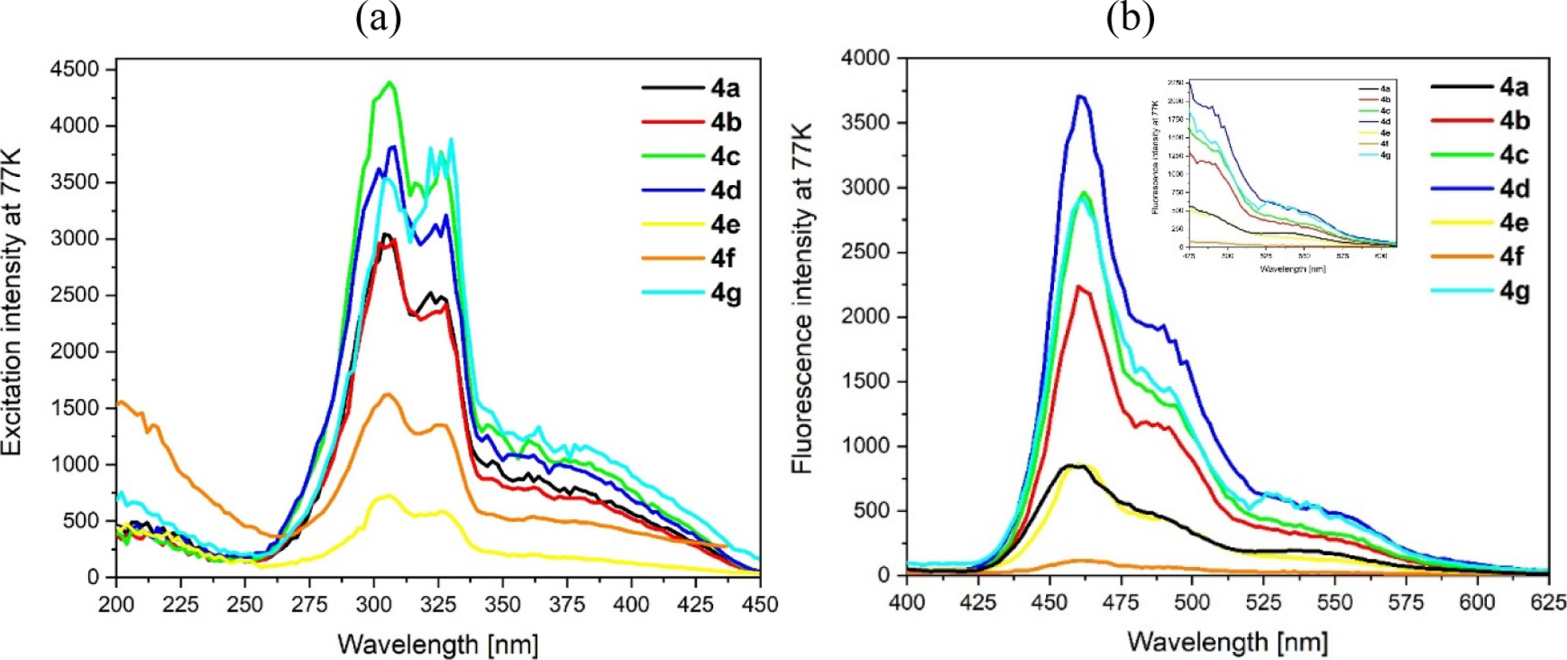
Excitation (a) and fluorescence (b) spectra of **4a**–**4g** recorded in MeOH/EtOH (4:1, *v*/*v*) at 77 K (*c* = 1.0 × 10^–5^ M).

The excitation spectra resemble the absorption spectra at 278 K, even though they are more structured, and the S2 transition is of the same intensity as the S3 band. Analysis at low temperature unveiled a correlation between the alkyl chain length at the carbazole moiety and the optical properties of compounds **4a**–**4g**. A lengthier alkyl chain corresponds to a more significant excitation and emission maxima shift toward longer wavelengths. Additionally, all compounds exhibit strong emissivity at 77 K, with emission intensity dependent on alkyl chain length. The observed Stokes shifts are large (>8000 cm^–1^), suggesting substantial geometric reorganization between ground and excited states, which is consistent with the “butterfly flattening” of the phenothiazine core (Tables S1 and S2).

The emission intensity gradually increases from compound **4a** to **4d**, decreases slightly for **4e**, and then increases again for **4f**, comparable to **4c**. Furthermore, compound **4f** displays an emission band with significantly reduced intensity compared to other compounds at the same concentration, likely due to the influence of the alkyl chain. Substantial differences are also observed in fluorescence decay times. The decay curves of **4a**–**4g** (Table 8 and Figure S11) exhibit two components, with a lower percentage share corresponding to a shorter lifetime, reflecting restricted molecular relaxation at 77 K and possible rigidified emissive conformers.

Furthermore, the maxima of absorption (υ_abs_) and fluorescence emission (υ_em_) wavelengths, presented in Table 9, were plotted as a function of the solvent polarity parameter *E*_T_ (30) for **4e**, as shown in Figure 5. The resulting Stokes shifts ranged from 6524 to 7100 cm^–1^ and correlated well with the solvent polarity parameters (*E*_T_(30) and Δ*f*).

**Table 9:** UV–Vis Absorption and Fluorescence Characteristics of **4e** in Different Solvents

solvent	Δ*f*	*E*_T_ (30) [kcal mol^–1^]	λ_abs_ [nm]	λ_em_ [nm]	υ_abs_ [cm^–1^]	υ_em_ [cm^–1^]	υabs-υem [cm^–1^]
**hx**	0.0012	30.9	355	462	28169	21645	6524
**PhCH**_3_	0.0131	33.9	358	476	27933	21008	6925
**DCM**	0.2171	40.7	358	478	27933	20921	7012
**MeOH**	0.3093	55.4	355	474	28169	21097	7072
**ACN**	0.3054	45.6	358	480	27933	20833	7100

**Figure 5:**
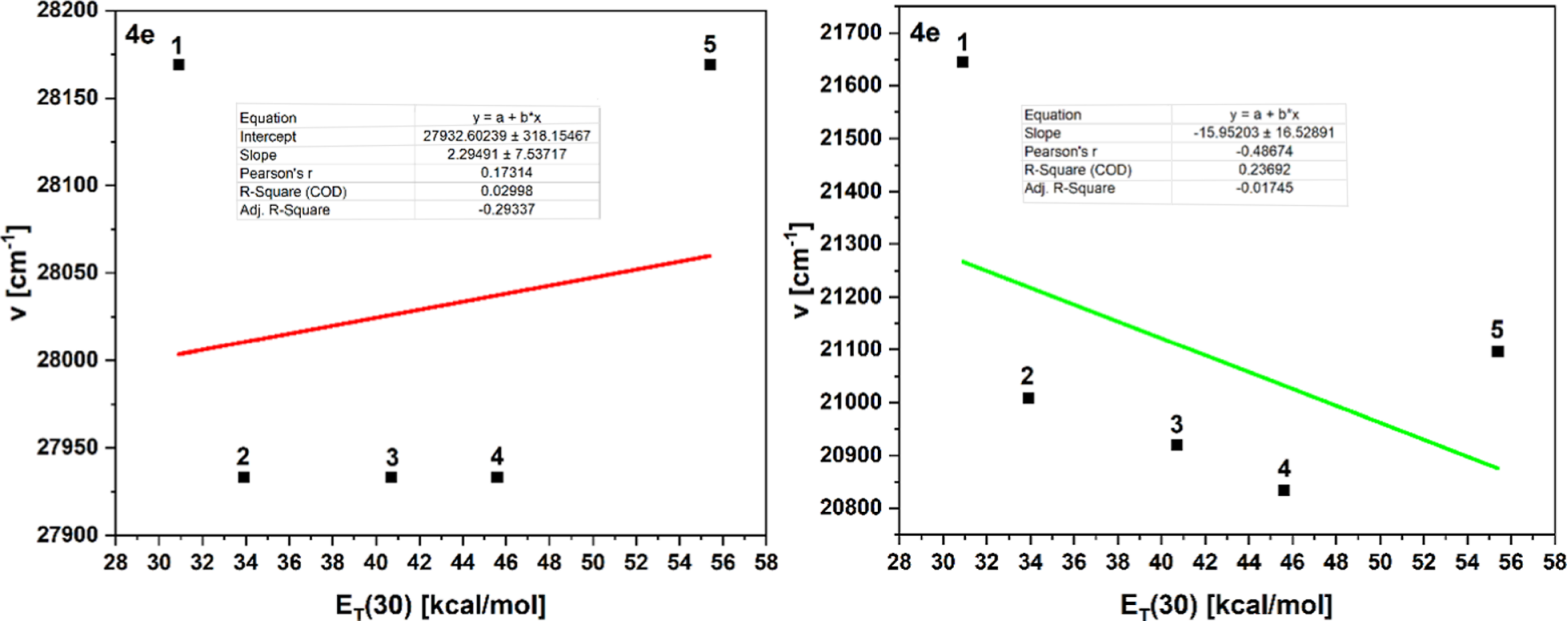
Dependence of υ_em_ (green line) and υ_abs_ (red line) of **4e** on solvent polarity ET (30) value. solvents: (1) hexane, (2) toluene, (3) dichloromethane, (4) methanol, (5) acetonitrile.

Emission changes (green line) showed a much better linear fit than absorption (red line). This may be because the solvatochromic effect is more visible in the ICT-excited state of the molecule than in the ground state. The same tendency was observed for the remaining compounds presented in the Supporting Information (Figures S12 and S13).

The results confirm that emission maxima exhibit a more pronounced dependence on solvent polarity than absorption maxima, consistent with the typical behavior of compounds exhibiting ICT. The Stokes shifts increased from 6524 cm^–1^ in hexane to 7100 cm^–1^ in acetonitrile, reflecting the stabilization of the polarized excited state in more polar environments.

The difference between the excited- and ground-state dipole moments can be estimated from a Lippert-Mataga plot, therefore, a representative Lippert-Mataga plot for molecule **4e** is presented in Figure 6. The determined R-squared value indicated the environmental sensitivity of the studied molecule.

**Figure 6:**
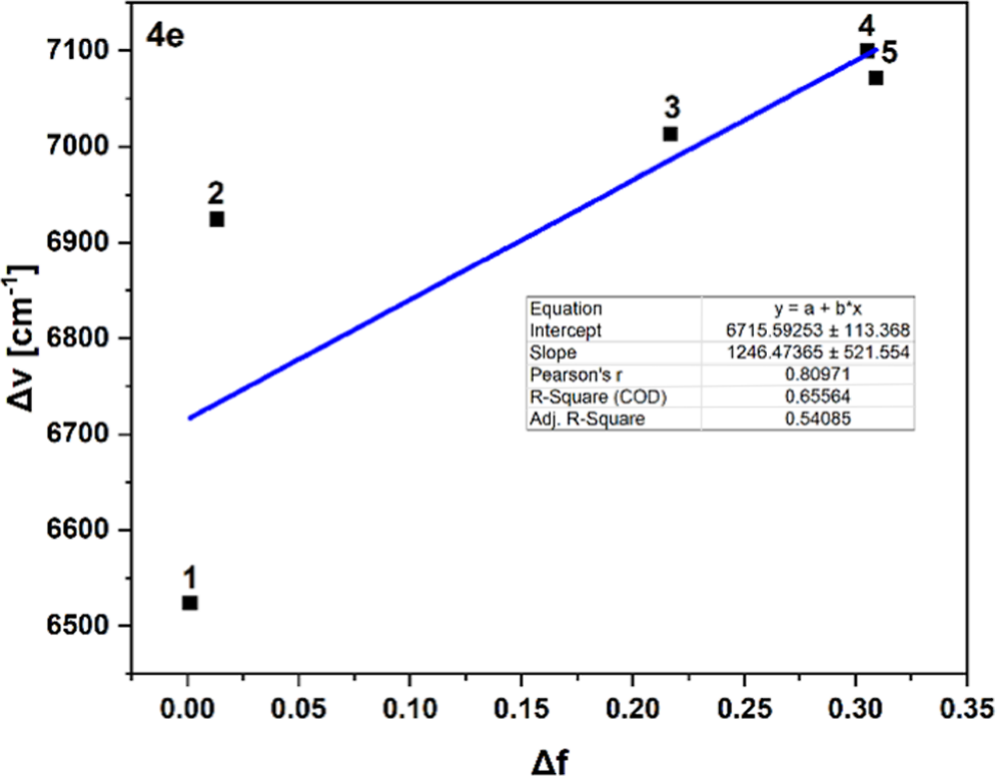
Stokes shift as a function of the solvent orientation polarizability (Δ*f*) for **4e**. Solvents: (1) hexane, (2) toluene, (3) dichloromethane, (4) methanol, (5) acetonitrile.

Based on the equation $\mu_{e}-\mu_{g}=\frac{\sqrt{Slope^{*}\kappa^{*}\alpha^{3}}}{3.33^{*}10^{-30}}$ , where μ_e_ and μ_g_ are the excited and the ground state dipole moment, respectively; α is the radius of the Onsager cavity taken from DFT calculations, assumed to be a sphere, and κ is a universal constant equal to 1.10511 × 10^–35^ C^2^. The calculated values of dipole moment difference between the ground and excited states (Δμ) for molecules **4a**–**4g** are presented in Table 10.

**Table 10:** Onsager Radius, Slope, and Change of the Dipole Moments of **4a**–**4g**

	onsager radius [Å]	slope [cm^–1^]	Δμ [Debye]
**4a**	6.03	1101.77	7.63
**4b**	6.25	958.93	8.06
**4c**	6.45	1126.27	8.45
**4d**	6.64	484.06	8.83
**4e**	6.82	1246.47	9.19
**4f**	6.99	681.26	9.54
**4g**	7.31	1087.49	10.21

The values of Δμ increase systematically with alkyl chain length from 7.63 D for **4a** to 10.21 D for **4g**, indicating a growing change in dipole moment upon excitation as the length of the alkyl chain at the carbazole units increases. This suggests that longer alkyl chains influence molecular geometry and aggregation behavior, thereby enhancing charge separation in the excited state. The calculated Onsager radii also increase from 6.03 Å (**4a**) to 7.31 Å (**4g**), consistent with the increasing molecular volume introduced by longer chains. This growth further supports the observed changes in dipole moment, indicating that the molecule’s spatial extension contributes to its solvatochromic sensitivity. These results confirm that structural modification via alkyl chain synthesis significantly affects the molecules’ excited-state polarity and solvatochromic response.

### 3.4 Electrochemical Properties

Cyclic voltammetry (CV) and differential pulse voltammetry (DPV) measurements were performed in DCM solution (concentration: 10^–3^ mol/dm^3^) with 1 mol/dm^3^ Bu_4_NPF_6_ electrolyte using a platinum electrode as the working electrode. Potentials concerning the ferrocene couple (Fc/Fc+) were referenced and used as the internal standard. The ionization potential (IP, marked as *E*_HOMO_) of Fc/Fc^+^ was calculated to be equal to −5.1 eV, as shown in.^47^ Under an oxidation process, the three peaks were well-defined in the differential pulse voltammetry method, and the voltammograms of the first oxidation process are presented in Figure 7. The data from the electrochemical investigation are collected in Table 11, and the DPV voltammograms are shown in Figure S14. The first oxidation peaks were registered in the range of 0.16–0.26 V vs Fc/Fc^+^ as quasi-reversible processes (Δ*E* < 150 mV) and can be assigned to the phenothiazine (PH) core oxidation and radical cation formation (PH•+), as reported.^48–51^ The **4a**–**4g** derivatives possessed low oxidation potentials compared to the similar compounds.^48^ The phenothiazine and carbazole motifs are electron-rich, which means the oxidation process is more straightforward to take place in contrast to other compounds (for example, phenothiazine with 2,2′-bithienyl, 9,9′-dibutylfluorenyl motifs or acceptor units).^49^ The other (*E*_ox_^2^ and *E*_ox_^3^) irreversible oxidation processes can be assigned to the subsequent oxidation of phenothiazine (diradical dication formation-PH^2+^),^52^ (about 0.8 V vs Fc/Fc^+^, Table 11) and/or oxidation of carbazole units.^52^

**Table 11:** Oxidation Potentials (vs Fc/Fc^+^) with *E*_LUMO,_ *E*_HOMO_, and the Optical Energy Band gap (*E*_g_^opt^) of Symmetrically Substituted Phenothiazine Derivatives **4a**–**4g** ^a^ ^c^

	method	*E*_ox_^1^	*E*_ox_^(onset)1^	*E*_ox_^2^	*E*_ox_^3^	*E*_LUMO_	*E*_HOMO_	*E*_g_^opt^
		[V]	[V]	[V]	[V]	[eV]	[eV]	[eV]
**4a**	DPV	0.22	0.12	0.78	1.23	–2.64	–5.22	2.58
	CV	0.24^b^	0.15	0.88^c^	1.43^c^	–2.67	–5.25	
**4b**	DPV	0.23	0.08	0.79	1.24	–2.59	–5.18	2.59
	CV	0.25^b^	0.14	0.72^c^	0.92^c^	–2.65	–5.24	
**4c**	DPV	0.17	0.01	0.61	1.09	–2.52	–5.11	2.59
	CV	0.25^b^	0.11	0.87^c^	1.38^c^	–2.62	–5.21	
**4d**	DPV	0.21	0.07	0.66	1.18	–2.58	–5.17	2.59
	CV	0.25^b^	0.12	0.87^c^	1.50^c^	–2.63	–5.22	
**4e**	DPV	0.21	0.08	0.65	1.15	–2.59	–5.18	2.59
	CV	0.23^b^	0.12	0.89^c^	1.47^c^	–2.63	–5.22	
**4f**	DPV	0.16	0.04	0.60	1.14	–2.56	–5.14	2.58
	CV	0.24^b^	0.08	0.82^b^	1.42^c^	–2.60	–5.18	
**4g**	DPV	0.26	0.10	0.73	1.23	–2.61	–5.20	2.59
	CV	0.24^b^	0.12	0.82^c^	1.42^c^	–2.63	–5.22	

a *Quasi*-reversible process.

b Irreversible process. *v* = 0.1 V/s for CV and *v* = 0.01 V/s for DPV measurements. *E*_ox_^(onset)1^ −the onset potential of the first reduction process. *E*_ox_ ^1–3^ −the first, the second and the third oxidation process.

c *E*_HOMO_ = −5,1-*E*_ox(onset)_^1^·|e|, *E*_LUMO_ = -*E*_g_^opt^ + IP, *E*_g_^opt^ calculated based on DCM solution. Solution: DCM, c: 10^–3^ mol/dm,^3^ electrolyte: 0.1 mol/dm^3^ Bu_4_NPF_6_. Pt as the working electrode.

**Figure 7:**
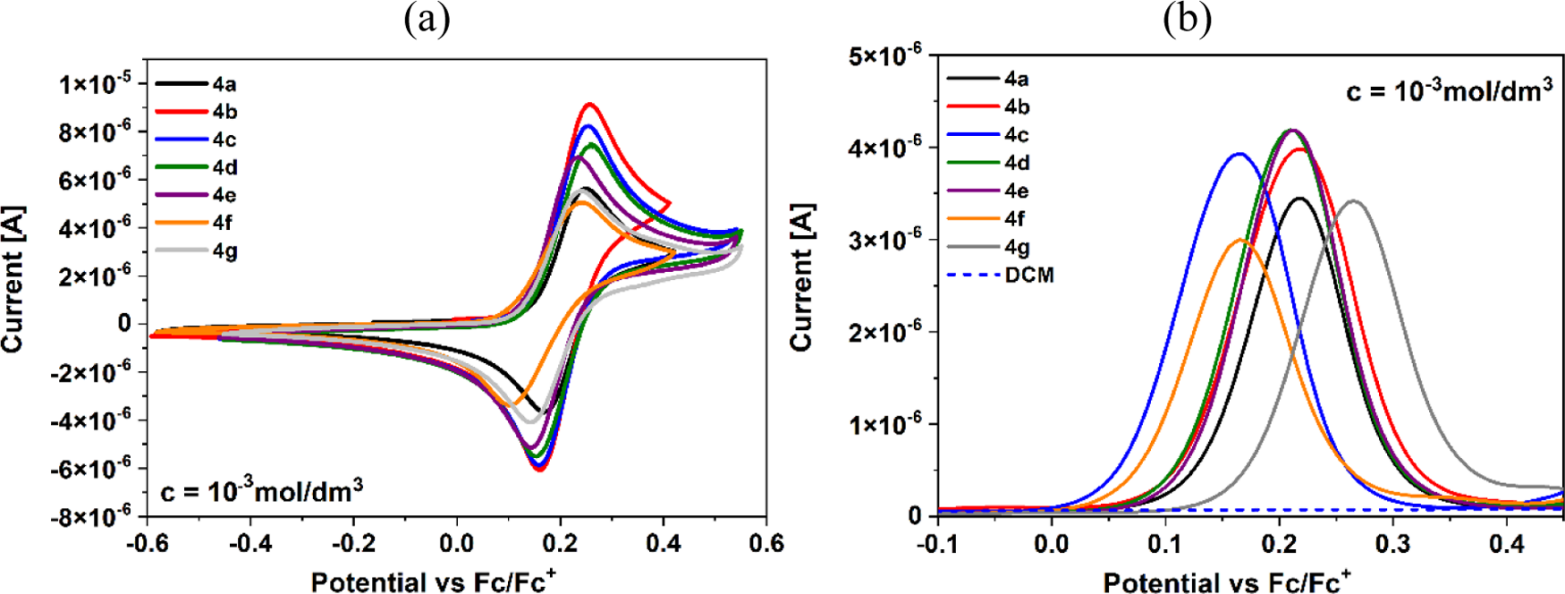
Voltammograms of the first oxidation process of symmetrically substituted phenothiazine derivatives for (a) CV (*v* = 0.01 V/s) and (b) DPV (*v* = 0.01 V/s) methods (Pt, 0.1 mol/dm^3^ Bu_4_NPF_6_ in DCM).

In further investigations, electropolymerization attempts were performed (Figure 8). The electropolymerization process at 1 V shows a shift in the first oxidation peak with increasing cycles (Figure 8a). Finally, the peaks were merged at about 0.8 V vs Fc/Fc^+^ into one broad peak. This may be related to the oxidation of phenothiazine to an unstable dicationic form, resulting in no cathodic response in subsequent scans.^52^ Moreover, the cathodic response at about −0.4 V and the anodic response at about −0.2 V were visible. The registration of these peaks may indicate a process leading to the formation of a 3,3′-bicarbazyl structure (2CR) between the molecule’s carbazole (CR) fragments. The proposed mechanism of the electropolymerization process (Figure S15) includes the formation of the PH dication diradical. It is worth noting that the observed process occurs at lower potentials than those reported in the literature for carbazole derivatives.^52,53^ The 3,3′-bicarbazyl structure is more readily oxidized than carbazole itself, and the presence of phenothiazine could facilitate the oxidation process at lower potentials. An increase in the oxidation and reduction currents is observed in subsequent scans at about 0.8 V, but this phenomenon is absent at lower potentials. This phenomenon may suggest carbazole oxidation, forming a 3,3′-carbaryl structure without polymerization or the formation of possible oligomers that do not polymerize due to steric hindrance.

**Figure 8:**
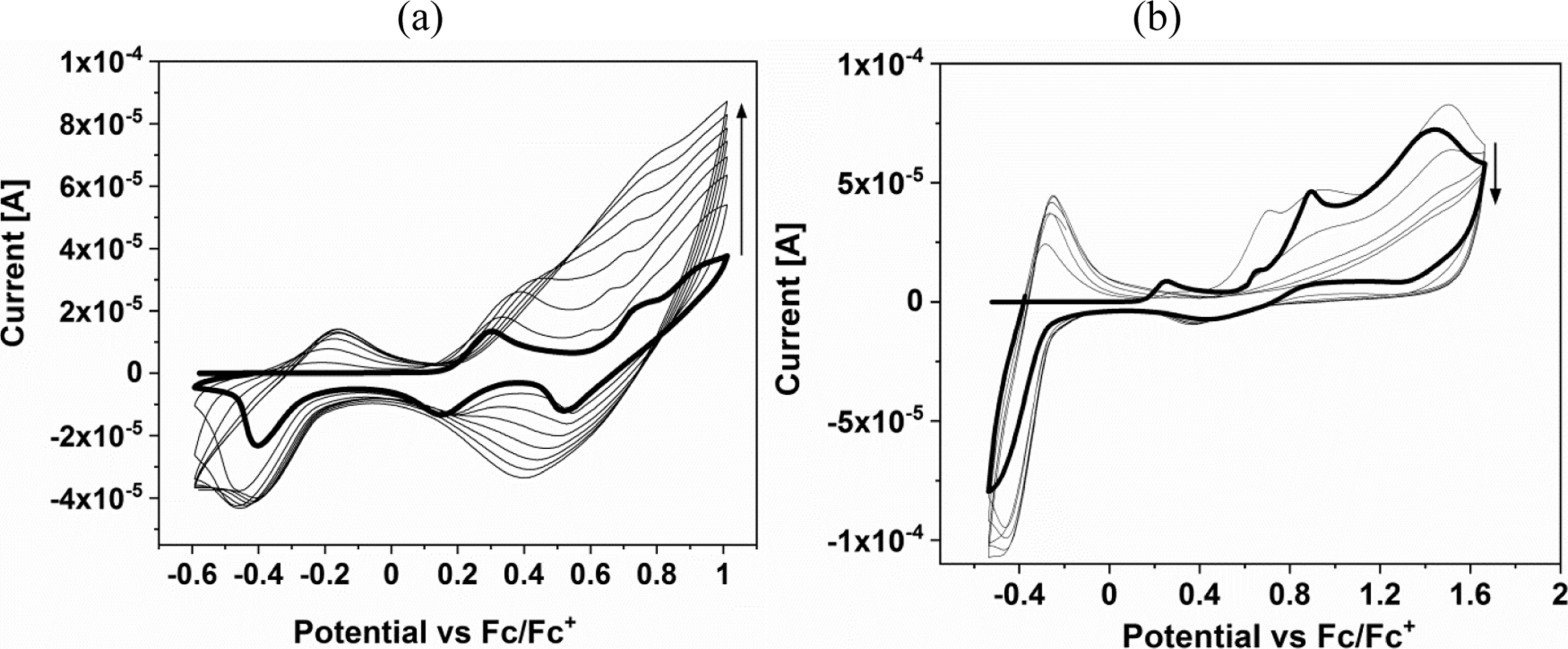
Voltammograms of the electropolymerization attempts to (a) 1 V and (b) 1.6 V of (a) **4b** and (b) **4a** (black solid line represents the first cycle; the gray solid lines represent subsequent oxidation scans, Pt, *v* = 0.1 V/s, 0.1 mol/dm^3^ Bu_4_NPF_6_ in DCM).

Furthermore, the different behaviors were recorded by increasing the cycles to 1.6 V potential (Figure 8b). A small peak was observed at about 0.7 V after the oxidation process of phenothiazine. Many processes can contribute to forming this peak, such as the adsorption of PH^+^ on the platinum electrode or the electrocrystallization of phenothiazine. In this case, the participation of the carbazole part of the molecule cannot be excluded, and the formation of an unstable intermediate product is possible.^52^ In the following cycles, this peak disappears, and no cathodic response is observed; the product obtained in this process is unstable. This behavior is more indicative of the formation of a dicationic phenothiazine without subsequent polymer/oligomer formation, as it blocks the reactive sites. In subsequent cycles, oxidation processes involving the carbazole moiety were observed. However, the two oxidation peaks were broader and lower after the two cycles than in the first and second cycles. That means the electropolymerization attempts failed because the irreversible degradation process was seen.^52^

The onset potentials of the first oxidation process (*E*_ox_^(onset)1^) allowed to estimate the ionization potentials (closely related to the HOMO level and marked in Table 11 as *E*_HOMO_) in the range from −5.11 to −5.25 eV vs Fc/Fc^+^. In the case of the electron affinities (closely related to the LUMO level and marked in Table 10 as *E*_LUMO_), the calculations were done by subtracting the *E*_g_^opt^ (optical band gap based on the λ_edge_ in DCM solution) from the ionization potentials and gave *E*_LUMO_ in the range of −2.52 to −2.67 V vs Fc/Fc^+^.

### 3.5 Cellular Fluorescence and Bioimaging

To evaluate the potential of phenothiazine-carbazole derivatives (**4a**–**4g**) as fluorescent probes in biological systems, in vitro experiments were conducted using several human cancer cell lines, including breast (MCF-7 cells), colon (HCT116 cells), and pancreatic (PANC-1 cells) cells. Breast cancer and gastrointestinal cancers, particularly colon cancers, are among the most prevalent in humans.^54^ In addition, pancreatic cancers are highly aggressive and are also difficult to diagnose.^55^ First, the cytotoxicity of the probes was assessed using the MTS test for metabolic activity. The results presented in Table S7 show that all compounds are noncytotoxic up to 25 μM against all of the tested cancer cell lines. Moreover, further tests confirmed that the compounds do not inhibit the proliferation of healthy fibroblast cells. The results obtained are satisfactory and reasonable for further imaging experiments.

Cells were incubated with the compounds at nontoxic concentrations for 1 or 2 h, and intracellular fluorescence was monitored using fluorescence microscopy. Under standard conditions, most compounds did not exhibit measurable intracellular fluorescence, suggesting limited passive membrane permeability or potential aggregation in the extracellular environment. Compounds **4a** and **4g**, bearing the shortest and longest alkyl chains, respectively, exhibited low solubility or precipitation in cell culture medium. This is probably due to unfavorable interactions with serum proteins, primarily bovine serum albumin (BSA), which is known to bind phenothiazine derivatives.^56^ In the case of **4a**, the short ethyl chains provide insufficient hydrophobic interaction for stable protein binding, resulting in low solubilization capacity and precipitation. Conversely, the long, highly hydrophobic hexadecyl chains in **4g** may promote aggregation or micelle-like self-association in the aqueous, protein-rich environment, thereby overwhelming the protein-binding capacity and leading to phase separation. Moreover, compound **4f**, despite being soluble and exhibiting good fluorescent properties in an abiotic environment, exhibited complete fluorescence quenching in the medium. This may be attributed to strong nonspecific binding or protein-induced conformational constraints that suppress emissive excited states. Alternatively, energy transfer or dynamic quenching processes involving serum proteins could explain the absence of emission.^57^ Such behavior highlights the complex interplay between molecular structure, solubility, and photophysical response in biologically relevant environments. These results emphasize the critical role of alkyl chain length beyond typical photophysical applications.

Nevertheless, the strong fluorescence of compounds **4b**–**4e** in solution in culture media prompted us to perform more specific cellular behavior tests. Given their limited ability to spontaneously penetrate intact cell membranes, we hypothesized that their intrinsic membrane impermeability could be exploited as a factor for a selective marker. We applied mild membrane-disruptive conditions to test this by supplementing 0.01% of Tween 20 to the media. Tween 20 (polysorbate 20) is a nonionic surfactant commonly used to permeabilize cell membranes gently. It reduces surface tension and can increase membrane fluidity, facilitating the uptake of otherwise membrane-impermeable compounds without causing significant cytotoxicity. The addition of a surfactant enables mild membrane permeabilization without causing detectable structural damage to the cells. Under these conditions, overall cellular morphology is preserved, allowing intracellular access while avoiding extensive membrane disruption. Such treatment may partially mimic apoptotic-like conditions, where increased membrane permeability occurs without immediate loss of cellular integrity.^58^

This approach may provide valuable insight into membrane integrity assays or targeted delivery systems. Fluorescence activation only under permeabilized conditions offers the potential for real-time visualization of membrane damage or selective uptake in diseased cells, such as tumors with compromised membranes, suitable for diagnostic or therapeutic applications. Compounds **4b** and **4c**, which showed the best solubility in aqueous media and stability in the presence of medium proteins, were selected for assays designed to evaluate cellular structure staining under membrane-permeabilization conditions. As anticipated, the selected compounds were able to enter the cells and resulted in persistent intracellular staining, showing bright green intracellular fluorescence (Figure 9). The emission is localized in distinct punctate cytoplasmic structures, suggesting endosomal or lysosomal accumulation. These observations indicate their ability to enter cells upon mild membrane permeabilization and maintain fluorescent properties in biologically relevant environments.

**Figure 9:**
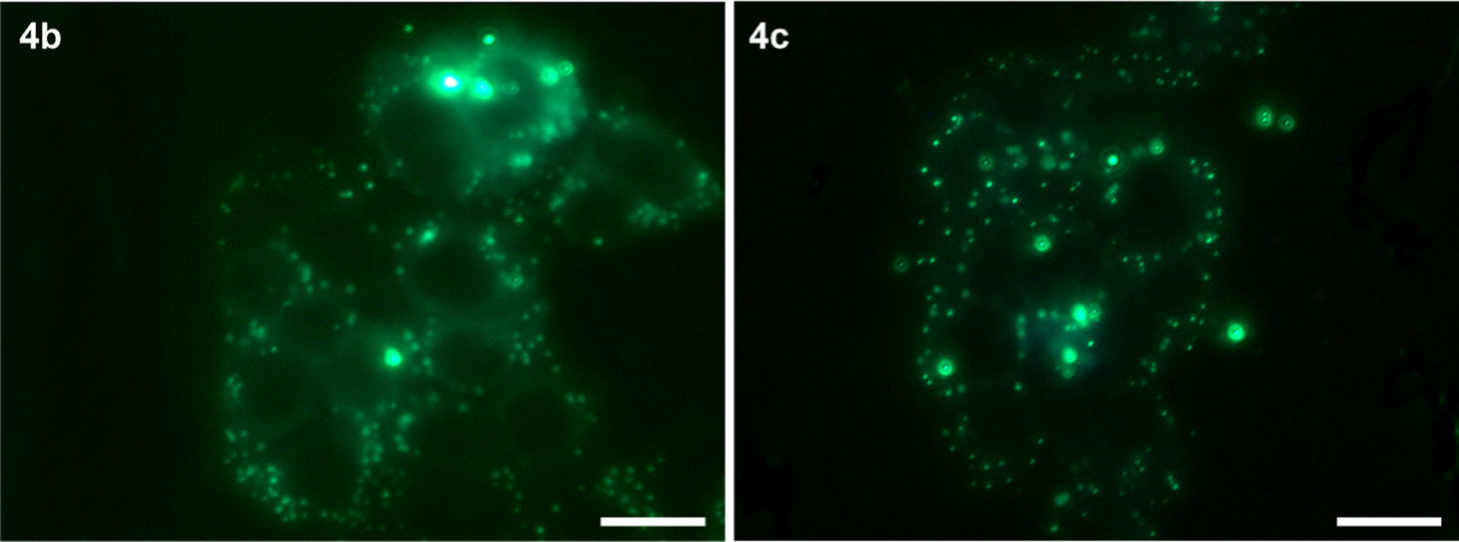
Fluorescence imaging of **4b** and **4c** at 25 μM in the MCF-7 cells under permeabilized conditions. Scale indicates 20 μm.

The observed cellular behavior correlates well with the photophysical and structural features of these compounds. Their high fluorescence quantum yields and relatively long emission lifetimes (∼2.3–2.5 ns at 77 K) indicate stable excited states and reduced susceptibility to nonradiative decay, both of which are crucial for achieving strong, sustained fluorescence signals in complex biological environments. This enhances environmental sensitivity, allowing the fluorophores to perform well in local polarity changes while avoiding strong quenching effects that would limit imaging contrast.

This selective entry under permeabilized conditions suggests potential use as fluorescence-based probes for monitoring membrane integrity or damage. Such compounds could serve as functional reporters in cytotoxicity assays, allowing for the visualization of early membrane disruption, or as tools in drug delivery systems (DDS), where specific membrane-disruptive stimuli, such as tumor microenvironments or external activation of DDS by ultrasound or light, trigger uptake. On the other hand, their stable fluorescence and low toxicity may be helpful in live cell imaging under controlled permeabilization protocols.

## 4 Conclusion

In this study, a novel series of symmetrically substituted *N*-ethylphenothiazine derivatives (**4a**–**4g**) containing carbazole units with systematically varied alkyl chain lengths (C2–C16) was successfully synthesized via the Sonogashira cross-coupling reaction. The influence of alkyl chain length on the photophysical, electrochemical, and structural properties of these compounds was thoroughly investigated, supported by DFT calculations. Photophysical studies revealed that all derivatives exhibit moderate positive solvatochromism with emission maxima red-shifted by up to 20 nm in polar solvents. Despite negligible solvent-dependent changes in absorption maxima, emission spectra were significantly more sensitive to polarity, reflecting stabilization of the excited state. Compounds with medium-length alkyl chains (**4b** and **4c**) exhibited optimal fluorescence performance, characterized by high quantum yields (up to 81%), long fluorescence lifetimes, and enhanced absorption coefficients, which can be attributed to improved molecular planarity and reduced aggregation. Low-temperature measurements at 77 K confirmed strong emissive behavior across the series. They revealed additional phosphorescence bands, suggesting the possibility of triplet state contributions and pointing to potential applications in phosphorescence. Dipole moment analysis and solvatochromic correlations further supported the ICT character and highlighted the effect of alkyl chain length on excited-state polarization, with the Δμ increasing from 7.63 for **4a** to 10.21 D for **4g**. Electrochemical analysis revealed low oxidation potentials and quasi-reversible processes associated with the oxidation of phenothiazine and carbazole. Although electropolymerization attempts demonstrated partial reactivity at elevated potentials, steric effects likely inhibited polymer growth. The cytotoxicity of the compounds was evaluated, confirming good cellular tolerance at the concentrations useful for bioimaging. Fluorescent staining experiments were conducted under both physiological and mildly permeabilized conditions. The results demonstrated that only compounds **4b** and **4c** produced detectable intracellular fluorescence upon membrane permeabilization, indicating their limited ability to cross intact membranes. This selective uptake highlights their potential utility as probes for monitoring membrane integrity or as tools for targeted delivery strategies, in which membrane-disrupting stimuli trigger cellular entry. This study demonstrates that variation of alkyl chain is an effective strategy for fine-tuning the photophysical and electrochemical properties of phenothiazine-based materials. These findings provide valuable insights for the rational design of fluorescent molecules and support the potential of such derivatives in applications such as OLEDs, sensing, and fluorescent probes.

## Supplementary Material



## Notes

Spectroscopic and theoretical analysis of *N*-ethylphenothiazine derivatives: alkyl chain length effects on solvatochromism, fluorescence efficiency, and excited-state polarity. Contribution of the particular molecules’ parts in creating orbitals HOMO–1, HOMO, LUMO, and LUMO+1 for **4a–4g** (Figure S1); calculated (B3LYP/6–311 + G­(d,p), DCM) angles of phenothiazine (Table S1); The optimized structures B3LYP/6–311 + G­(d,p), DCM of excited states (T_1_ and S_1_) of molecules **4a**–**4g** with contours of Frontier orbitals (Table S2); energies (6–311 + G­(d,p)/B3LYP) of HOMO and LUMO of ground state S_0_, singlet excited state S_1_, and triplet T_1_ and calculated energy differences between T_1_ and S_1_ for **4a**–**4g** (Table S3); UV–vis spectra of **4b**–**4d** and **4f**–**4g** in different solvents (*c* = 1.0 × 10^–5^ M) (Figure S2); Fluorescence spectra of **4b**–**4d** and **4f**–**4g** in different solvents (*c* = 1.0 × 10^–5^ M) (Figure S3); Photophysical data for **4a**–**4g** recorded in different solvents (Table S4); The calculated dipole moments of the ground μ_g_ and the excited state μ_e_ of **4a**–**4g** in various solvents B3LYP/6–311 + G­(d,p) (Table S5); fluorescence decays of **4a** recorded in different solvents (Figure S4); fluorescence decays of **4b** recorded in different solvents (Figure S5); Fluorescence decays of **4c** recorded in different solvents (Figure S6); fluorescence decays of **4d** recorded in different solvents (Figure S7); fluorescence decays of **4e** recorded in different solvents (Figure S8); fluorescence decays of **4f** recorded in different solvents (Figure S9); fluorescence decays of **4g** recorded in different solvents (Figure S10); fluorescence decays of solutions **4a**-**4g** in EtOH/MeOH (4:1, v/v) recorded at low-temperature 77K (Figure S11); dependence of υ_em_ (green line) and υ_abs_ (red line) for **4a**–**4d**, and **4f**–**4g**. on solvent polarity ET (30) value. solvents: (1) hexane, (2) toluene, (3) dichloromethane, (4) methanol, (5) acetonitrile (Figure S12); stokes shift as a function of the solvent orientation polarizability (Δ*f*) for **4a**–**4d**, and **4f**–**4g**. Solvents: (1) hexane, (2) toluene, (3) dichloromethane, (4) methanol, (5) acetonitrile (Figure S13); the voltammograms of **4a**–**4g** in DPV method (*v* = 0.01 V/s, Pt, 0.1 mol/dm^3^ Bu_4_NPF_6_ in DCM) (Figure S14); The proposed mechanism of the electropolymerization process of carbazole fragments of the molecule with the phenothiazine dication diradical formation (Figure S15); Electron density difference (Δρ) maps for compounds **4c**–**4g** (Table S6); cytotoxicity of **4a**–**4g** and doxorubicin (DOX) against cancer and normal cells (Table S7); The ^1^H and ^13^C spectra of **4a**-**4g** in CDCl_3_ (Figure S16–S29) (PDF)
