# Supplementary material for: Spectroscopic and Theoretical Analysis of N‑Ethylphenothiazine Derivatives: Alkyl Chain Length Effects on Solvatochromism, Fluorescence Efficiency, and Excited-State Polarity
Source: J Phys Chem B. 2026 Feb 25;130(10):2843–58. doi: 10.1021/acs.jpcb.5c08011 (PMC13362198; doi:10.1021/acs.jpcb.5c08011)
Supplement: Supplementary file 1 [file jp5c08011_si_001.pdf]

Spectroscopic and Theoretical Analysis of N-Ethylphenothiazine Derivatives: Alkyl Chain Length Effects on Solvatochromism, Fluorescence Efficiency, and Excited-State Polarity

Aneta Slodek[a*], Dawid Zych[b], Sylwia Zimosz[a], Grażyna Szafraniec-Gorol[a], Sonia Kotowicz[a], Martyna Kubis[a], Katarzyna Kowalska-Szojda[a], Katarzyna Malarz[c], Robert Musioł[a]

[a] Institute of Chemistry, University of Silesia, Szkolna 9, 40-006 Katowice, Poland
[b] Institute of Chemistry, Faculty of Chemistry and Pharmacy, University of Opole, Oleska 48, 45-052 Opole, Poland
[c] Institute of Physics, Faculty of Science and Technology, University of Silesia, 75 Pułku Piechoty 1A, 41-500 Chorzów, Poland

*Corresponding Author: a.slodek@wp.pl, aneta.slodek@us.edu.pl (A. Slodek)

General Methods

The NMR spectra were recorded on a Bruker Avance 400 MHz instrument using $CDCl_3$ as a solvent. Elemental analysis was performed using a FlashSmart CHNS/O analyzer. The absorption spectra were measured at ambient temperature on a Shimadzu UV-1900i spectrophotometer using a standard 1.0 cm quartz cell. The emission and excitation spectra were measured using the FS 5 spectrofluorometer at both ambient and low temperatures (in liquid nitrogen) with a 150 W Xe lamp as the light source. The absolute method, performed at room temperature, determined fluorescence quantum yields using an integrating sphere with solvent as the blank. Compounds are excited at the wavelength corresponding to the absorption wavelength of the compounds. The time-resolved measurement was obtained from optically diluted solutions at room temperature, using time-correlated single-photon counting (TCSPC) on the FS 5 spectrofluorometer. Excitation wavelengths were obtained using the picosecond-pulsed diode EPL-375 nm with a 100 ns pulse period as the light source. The system was aligned at emission wavelengths. Additionally, an instrument response function needs to be obtained to analyse fluorescence decay. The IRF contains information about the time response of the overall optical and electronic system. The IRF was measured using a LUDOX solution standard at 375 nm.
Redox properties were investigated using two measurement methods: cyclic voltammetry (CV) and differential pulse voltammetry (DPV). The results were recorded on an Eco Chemie Autolab PGSTAT128n potentiostat using a glassy carbon electrode (Ø 2.0 mm) as the working electrode, 0.1 mol/dm3 Bu4NPF_6 (Sigma-Aldrich, 99%) electrolyte, and a dichloromethane solution (Sigma-Aldrich, 99.8%, CH_2Cl_2) at a concentration of 10^{-3} mol/dm^3. The platinum coil and silver wire were used as the auxiliary and reference electrodes, respectively. The moderate scan rate for the cyclic voltammetry method was 0.10 V/s, and the differential pulse voltammetry method was 0.01 V/s. The solutions were purged with argon for approximately

12 minutes before each measurement and were performed at 25 ± 1 °C. The ferrocene couple (Fc/Fc^+) was used as the internal standard, and the E_{HOMO} of Fc/Fc^+ was calculated to equal -5.1 eV, as shown in the publication [1].

Cell culture

The human colon cancer cell line MCF-7, human breast cancer cell line, and human colon carcinoma cell line HCT116 were purchased from ATCC, while the human pancreas ductal adenocarcinoma cell line PANC-1 was obtained from Sigma-Aldrich. The normal human dermal fibroblast cell line NHDF was obtained from PromoCell. The cancer cell lines were cultured in Dulbecco's modified Eagle's medium (DMEM) supplemented with 10% heat-inactivated fetal bovine serum (FBS; all from Merck) in 75 cm2 flasks (Nunc). The NHDF cells were cultured in Fibroblast Growth Medium (FGM) with low serum content. Each medium contained a combination of two antibiotics: penicillin and streptomycin (1% v/v; Gibco). These cell lines were grown under standard conditions at 37 °C with a 5% CO_2 humidified atmosphere.

Cytotoxicity studies

The cells were seeded in 96-well clear plates (Nunc) at a density of 5000 cells/well (cancer cells) and 4000 cells/well (normal cells) and incubated under standard conditions for 24 hours. Stock solutions of the tested compounds were prepared in DMSO. Afterwards, the solutions of the tested phenothiazines, prepared at concentrations ranging from 0 to 25 µM (with DMSO concentration not exceeding 0.3%), were added to the plate in DMEM. The MTS assay was performed after a 72-hour incubation, according to the manufacturer's instructions. Briefly, the tested solutions were removed from each well and replaced with 100 µL DMEM (without phenol red and FBS) with 20 µL of the CellTiter 96®AQueous One Solution-MTS (Promega) and incubated for 1 h or 3 h (for PANC-1 cells) at 37 °C. The optical densities of the samples were measured at 490 nm using a multi-plate reader VarioSkan LUX (Thermofisher). The results were compared to the control and estimated as the inhibitory concentration (IC50) values. Each compound was tested in triplicate in a single experiment, with each experiment being performed three or four times.

Cellular staining

Before cellular staining experiments, MCF-7 cells were seeded onto coverslips at a density of 150.000 cells/slide and incubated at 37 °C for 48 h. Then, the DMEM was removed, and solutions of the tested compounds (4a-g) at a concentration of 25 µM were added, and the mixture was further incubated for 1 h. Then, the cells were washed three times with PBS and mounted with DMEM without FBS or phenol red. The cellular staining results were immediately observed after excitation at 365 nm LED illumination (25% of power) using a Zeiss Axio Observer.Z1 inverted fluorescence microscope equipped with a color AxioCam MRm camera.

Cellular staining with permeabilization

The MCF-7 cells were seeded as described above. After 48 h, the DMEM was removed, and the solution of 0.01% Tween 20 was added to permeabilize the cell membrane. After 1 h, the solutions of the tested compounds (4b and 4c) at 25 μM were added, and the mixture was further incubated for 1 h. Then, the cells were washed three times with PBS and mounted with DMEM without FBS or phenol red. The cellular staining results were immediately observed using a Zeiss Axio Observer.Z1 inverted fluorescence microscope equipped with a color AxioCam MRm camera.

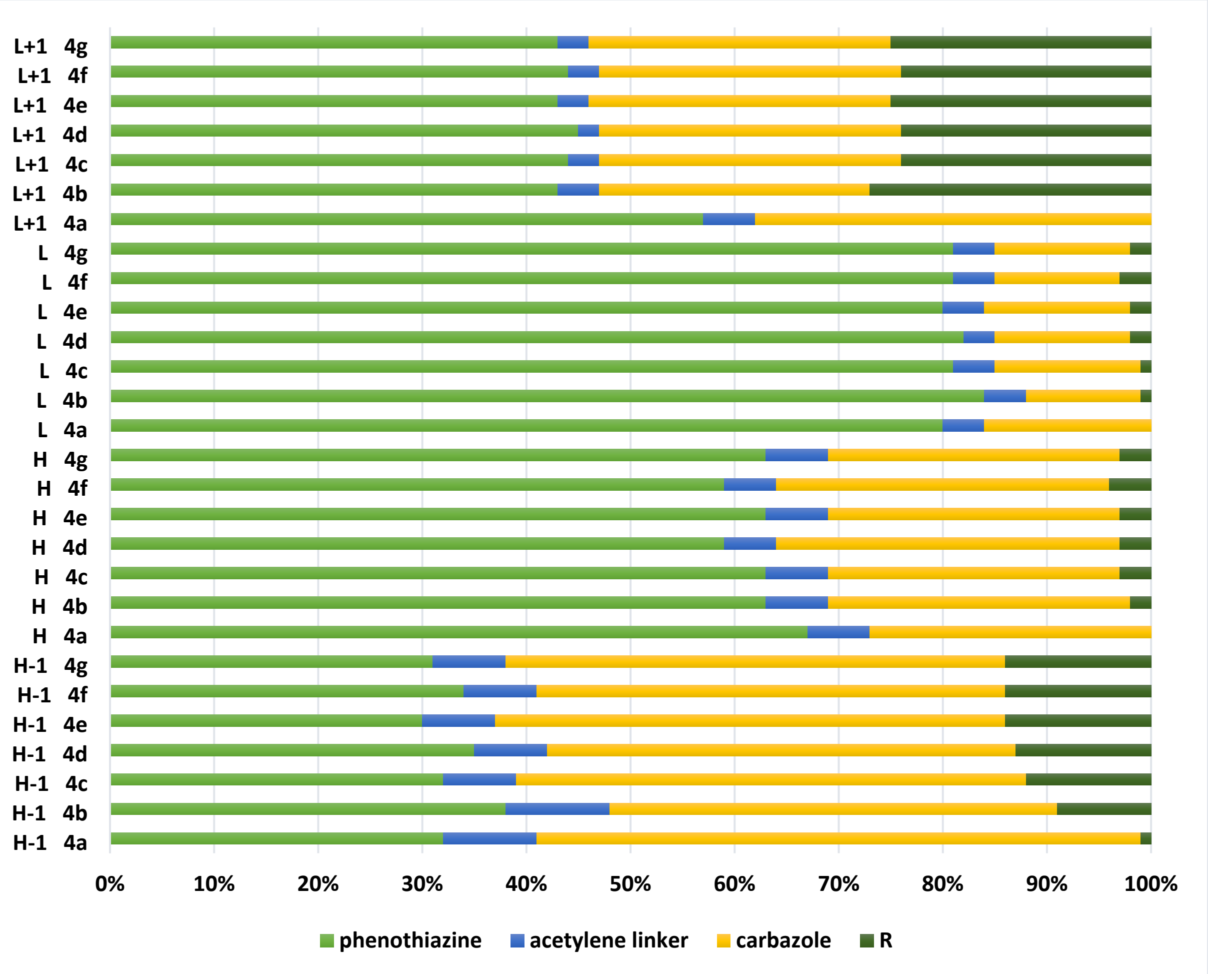

Figure S1. Contribution of the particular molecules' parts in creating orbitals HOMO-1, HOMO, LUMO, and LUMO+1 for **4a-4g**.

Table S1. Calculated (B3LYP/6-311+G(d,p), DCM) angles of phenothiazine.

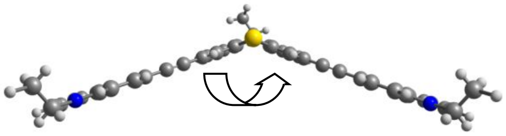

	ground state S_0 [°]	excited state T_1 [°]	excited state S_1 [°]	Δ (T_1- S_0) [°]	Δ (S_1- S_0) [°]
**4a**	138.64	167.65	166.47	29.01	27.83
**4b**	138.74	167.89	166.60	29.15	27.86

**4c**	138.50	168.32	166.88	29.82	28.38
**4d**	138.69	168.13	166.82	29.44	28.13
**4e**	138.44	167.83	166.33	29.39	27.89
**4f**	138.69	168.10	166.78	29.41	28.09
**4g**	138.54	168.21	166.92	29.67	28.38

Table S2. The optimized structures B3LYP/6-311+G(d,p), DCM of excited states (T_1 and S_1) of molecules **4a-4g** with contours of frontier orbitals.

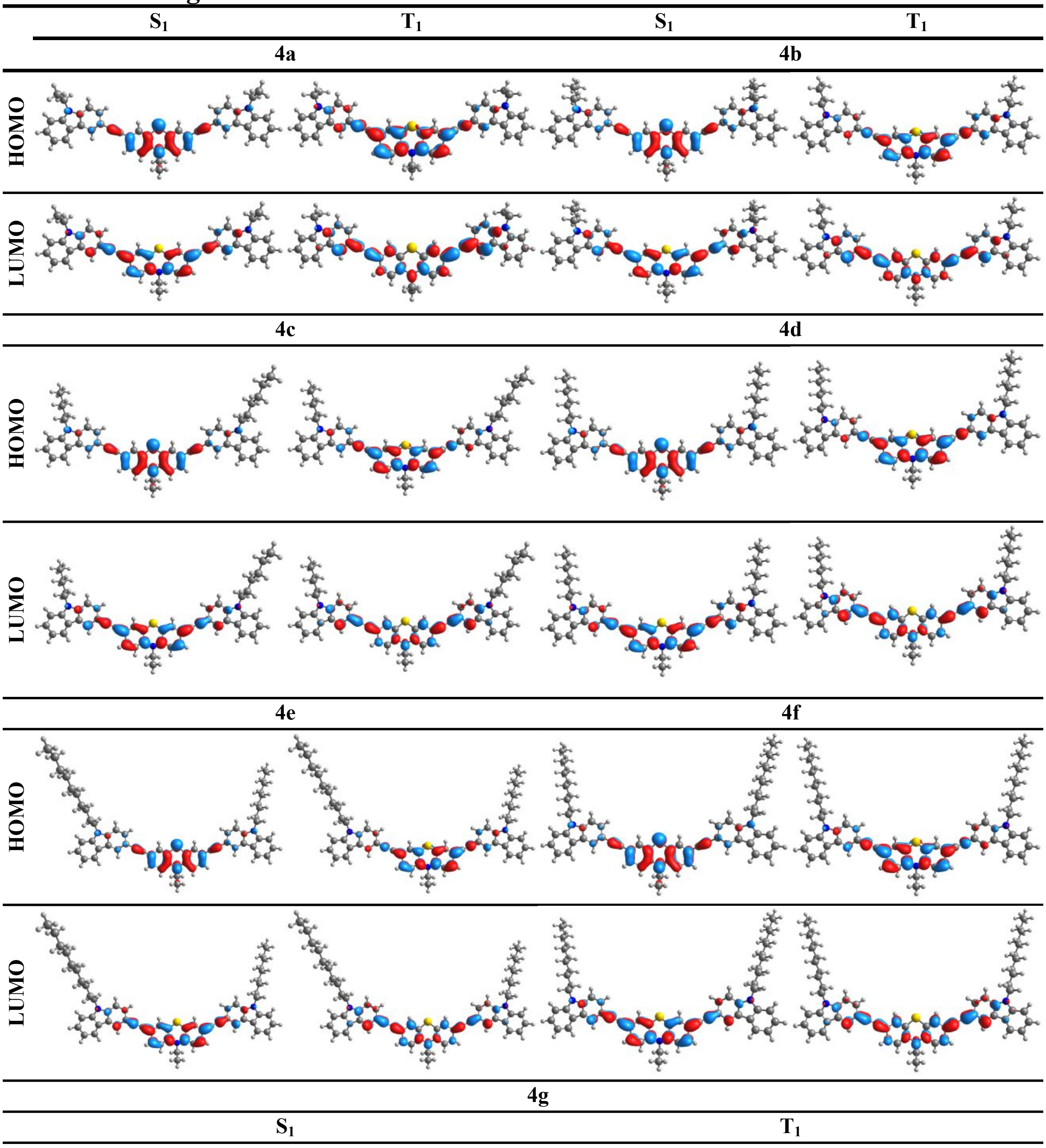

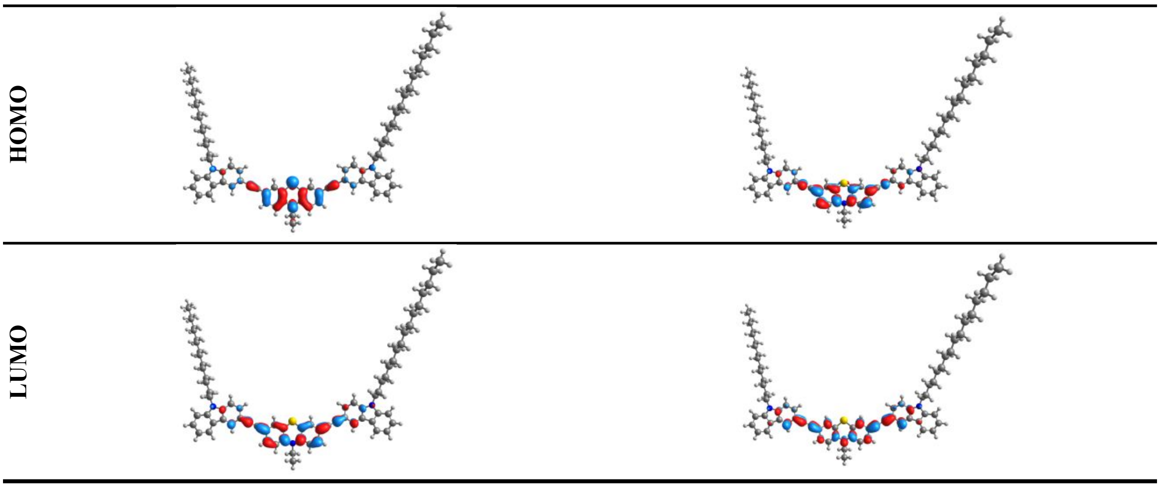

Table S3. Energies (6-311+G(d,p)/B3LYP, DCM) of HOMO and LUMO of ground state S_0, singlet excited state S_1, and triplet T_1 and calculated energy differences between T_1 and S_1 for **4a-4g**.

	E_{HOMO} [eV]			E_{LUMO} [eV]			ΔE_{T1-S1} [cm^{-1}]
	S_0	S_1	T_1	S_0	S_1	T_1	
**4a**	-5.23	-4.83	-2.88	-1.65	-1.93	-1.59	2654
**4b**	-5.22	-4.82	-2.88	-1.64	-1.92	-1.59	2653
**4c**	-5.22	-4.82	-2.88	-1.64	-1.92	-1.59	2654
**4d**	-5.22	-4.82	-2.88	-1.64	-1.93	-1.59	2655
**4e**	-5.22	-4.83	-2.88	-1.64	-1.92	-1.59	2653
**4f**	-5.22	-4.82	-2.88	-1.64	-1.92	-1.59	2654
**4g**	-5.22	-4.82	-2.88	-1.64	-1.92	-1.59	2654

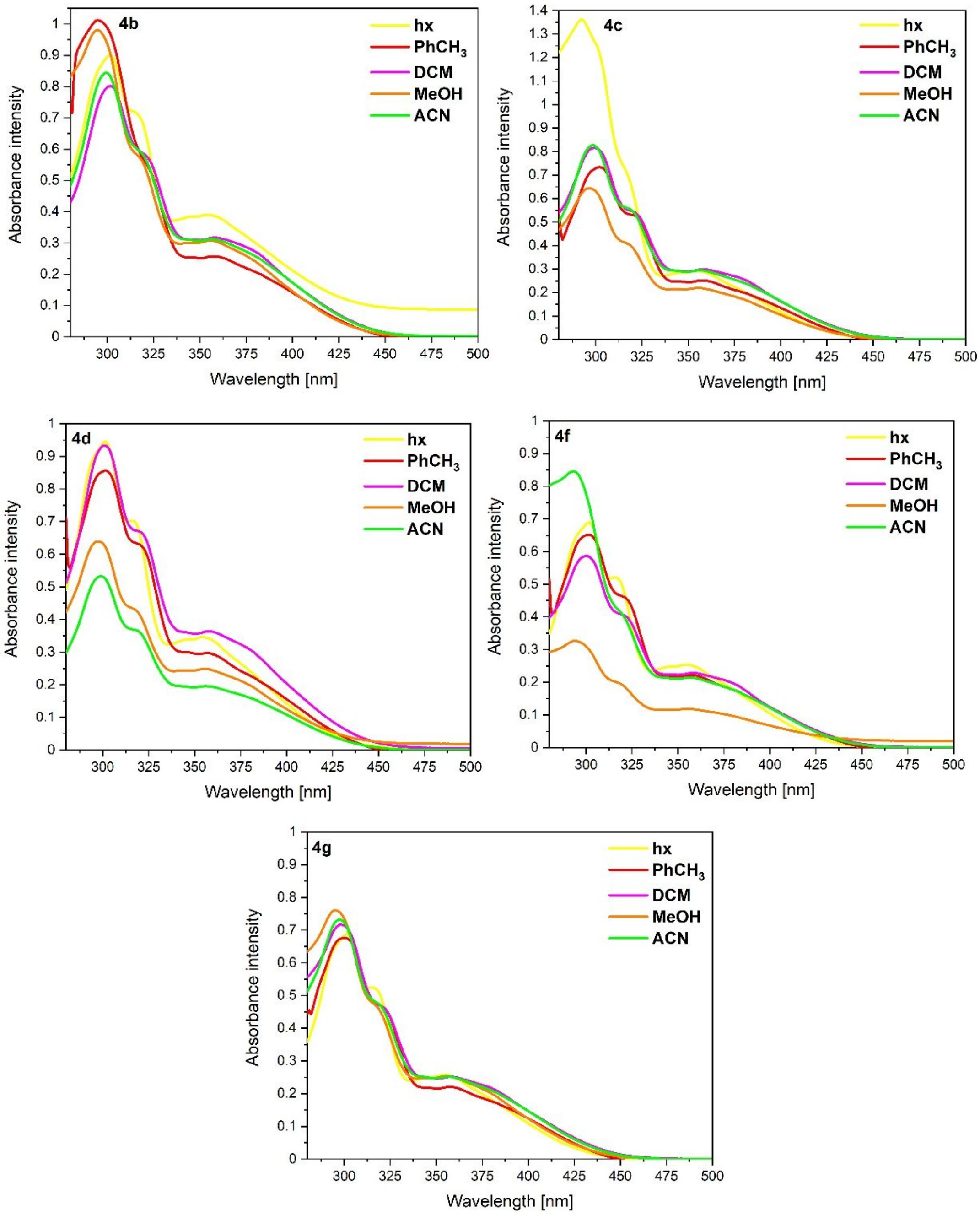

Figure S2. UV-Vis spectra of **4b-4d** and **4f-4g** in different solvents (c = 1.0 × 10 $^{-5}$ M).

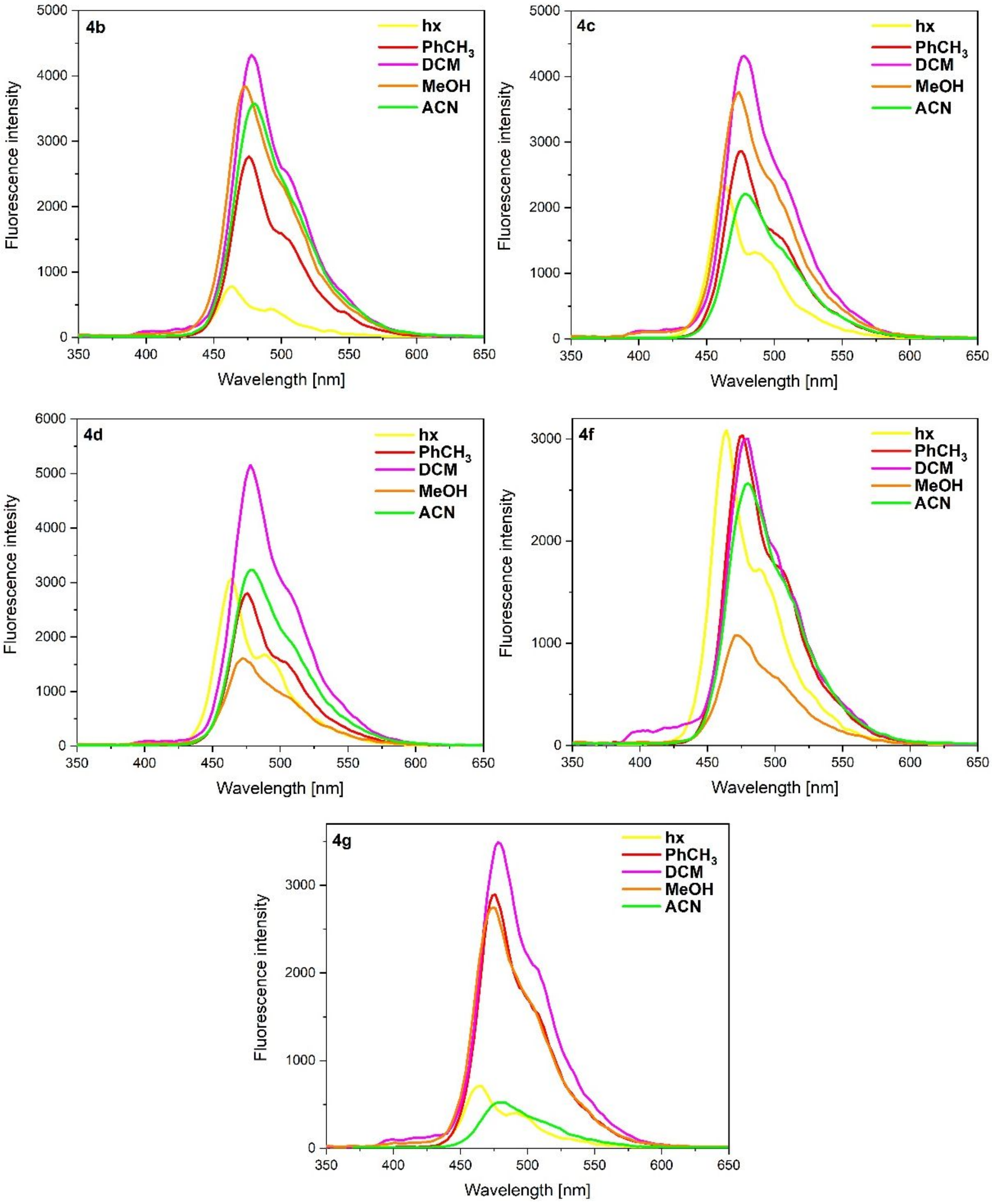

Figure S3. Fluorescence spectra of **4b-4d** and **4f-4g** in different solvents (c = 1.0 × 10 $^{-5}$ M).

Table S4. Photophysical data for **4a-4g** recorded in different solvents.

**Solvent**	τ_{eff} **[ns]**						
	**4a**	**4b**	**4c**	**4d**	**4e**	**4f**	**4g**
**Hx**	2.16	2.20	2.23	2.22	2.19	2.21	2.14
χ^2	1.089	1.113	1.149	1.149	1.194	1.126	1.197
**$PhCH_3$**	3.00	3.04	3.03	2.80 [0.09 (8.46%) 3.05 (91.54%)]	3.08	2.78 [0.07 (9.29%) 3.06 (90.71%)]	3.02
χ^2	1.088	0.999	1.126	1.290	0.967	1.197	1.097

**DCM**	2.69 [3.36 (79.60%) 0.09 (20.40%)]	3.01 [0.03 (12.99%) 3.45 (87.01%)]	3.23 [0.36 (7.46%) 3.46 (92.54%)]	3.35	3.35	2.93 [0.18 (14.00%) 3.38 (86.00%)]	3.05 [0.14 (10.96%) 3.41 (89.04%)]
χ^2	0.987	1.166	1.015	1.106	1.175	1.109	1.164
**MeOH**	3.13	2.92 [0.25 (9.26%) 3.19 (90.74%)]	2.78 [0.27 (14.21%) 3.19 (85.79%)]	2.87 [0.23 (10.44%) 3.18 (89.56%)]	2.95 [0.44 (8.91%) 3.20 (91.09%)]	2.79 [0.12 (12.24%) 3.16 (87.76%)]	2.76 [0.23 (13.52%) 3.16 (86.48%)]
χ^2	1.149	1.136	0.995	1.072	1.009	1.161	1.095
**ACN**	3.34	3.33	3.31	3.36	3.35	3.30	3.32
χ^2	1.050	0..961	1.109	1.014	1.116	1.109	0.991

Table S5. The calculated dipole moments of the ground μ_g and the excited state μ_e of **4a-4g** in various solvents B3LYP/6-311+G(d,p).

**Solvent**	**4a**			**4b**			**4c**			**4d**		
	μ_g	μ_e	$\Delta\mu$	μ_g	μ_e	$\Delta\mu$	μ_g	μ_e	$\Delta\mu$	μ_g	μ_e	$\Delta\mu$
**hx**	2.36	1.71	-0.65	3.11	1.85	-1.26	2.95	1.98	-0.97	3.32	2.00	-1.32
**$PhCH_3$**	2.43	1.73	-0.70	3.16	1.87	-1.29	3.00	2.00	-1.00	3.37	1.98	-1.39
**DCM**	2.67	1.66	-1.01	3.31	1.81	-1.50	3.16	1.66	-1.50	3.45	1.80	-1.65
**MeOH**	2.77	1.62	-1.15	3.34	1.78	-1.56	3.21	1.58	-1.63	3.43	1.71	-1.72
**ACN**	2.77	1.63	-1.14	3.34	1.79	-1.55	3.21	1.79	-1.42	3.43	1.78	-1.65

**Solvent**	**4e**			**4f**			**4g**		
	μ_g	μ_e	$\Delta\mu$	μ_g	μ_e	$\Delta\mu$	μ_g	μ_e	$\Delta\mu$
**hx**	3.04	2.04	-1.00	3.38	2.00	-1.38	3.04	2.00	-1.04
**$PhCH_3$**	3.08	2.05	-1.03	3.42	1.99	-1.43	3.08	1.99	-1.09
**DCM**	3.25	1.83	-1.42	3.48	1.79	-1.69	3.17	1.76	-1.41
**MeOH**	3.32	1.89	-1.43	3.48	1.73	-1.75	3.17	1.86	-1.31
**ACN**	3.32	1.89	-1.43	3.48	1.73	-1.75	3.17	1.86	-1.31

Table S6. Electron density difference ($\Delta\rho$) maps for compounds **4c**-**4g**.

**4c**	**4d**
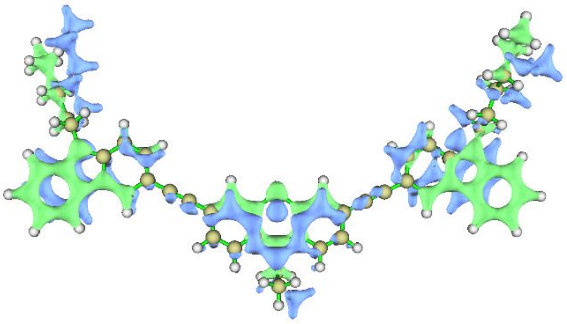	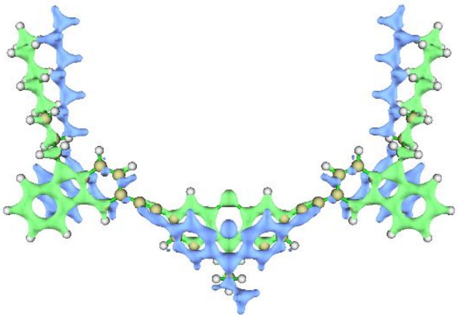
**4e**	**4f**
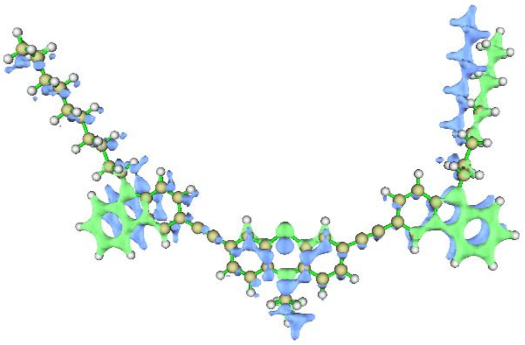	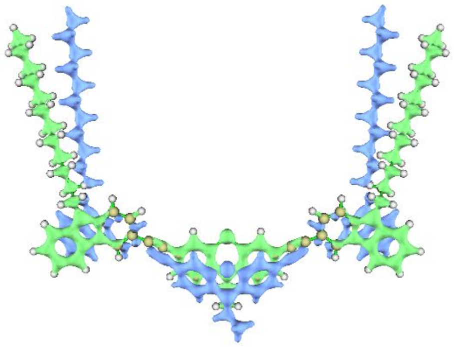
**4g**	
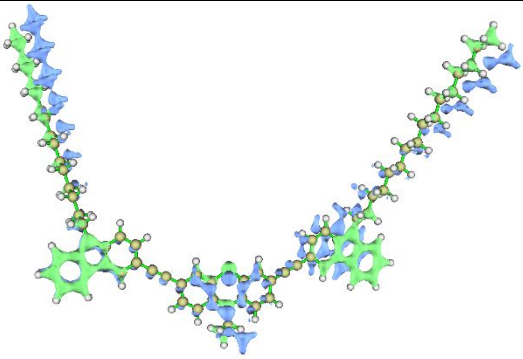	

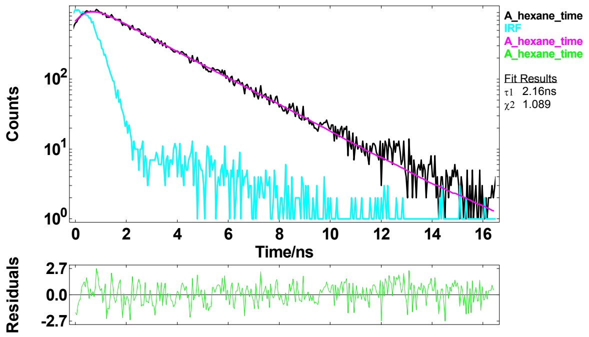

hx

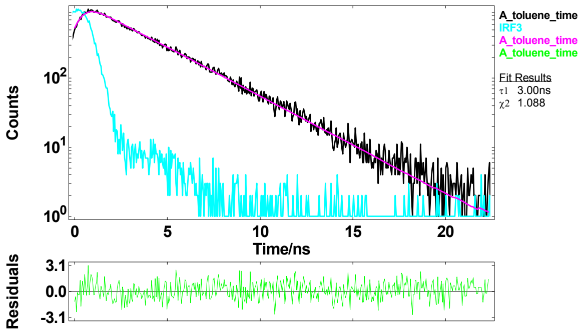

$PhCH_3$

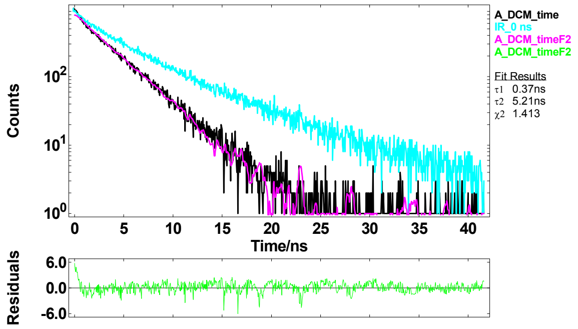

CH_2Cl_2

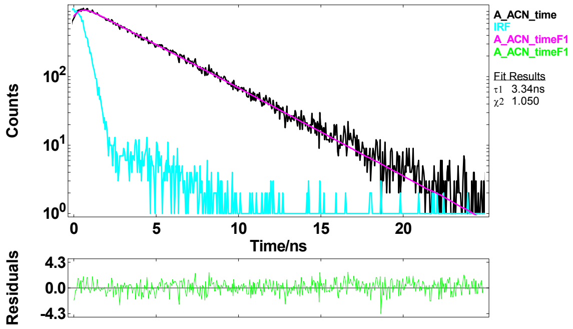

ACN

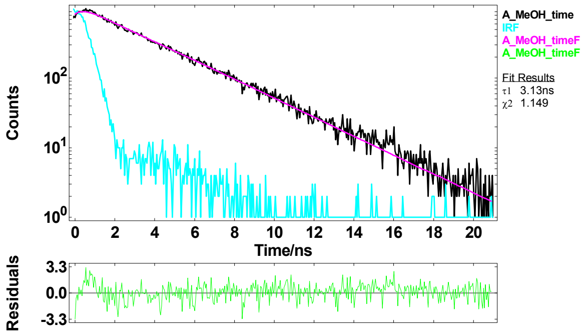

MeOH

Figure S4. Fluorescence decays of **4a** recorded in different solvents.

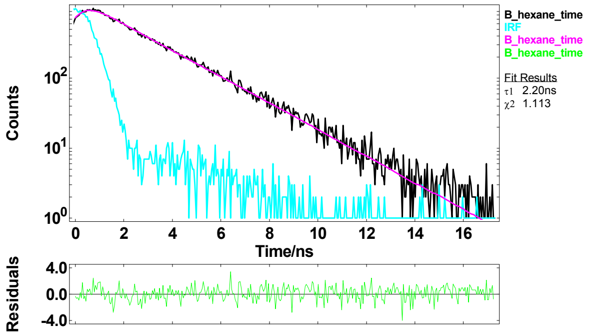

hx

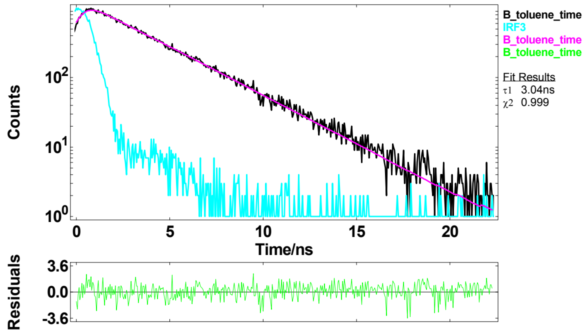

$PhCH_3$

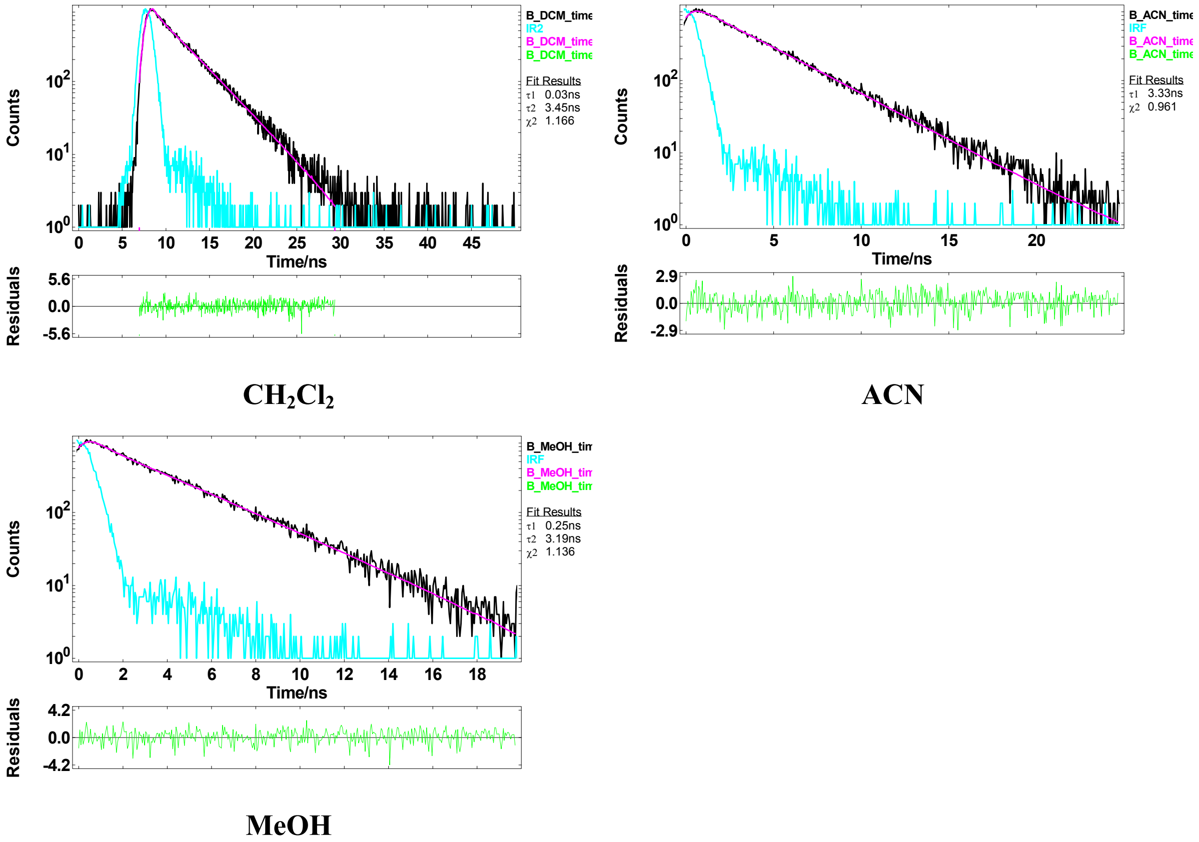

Figure S5. Fluorescence decays of **4b** recorded in different solvents.

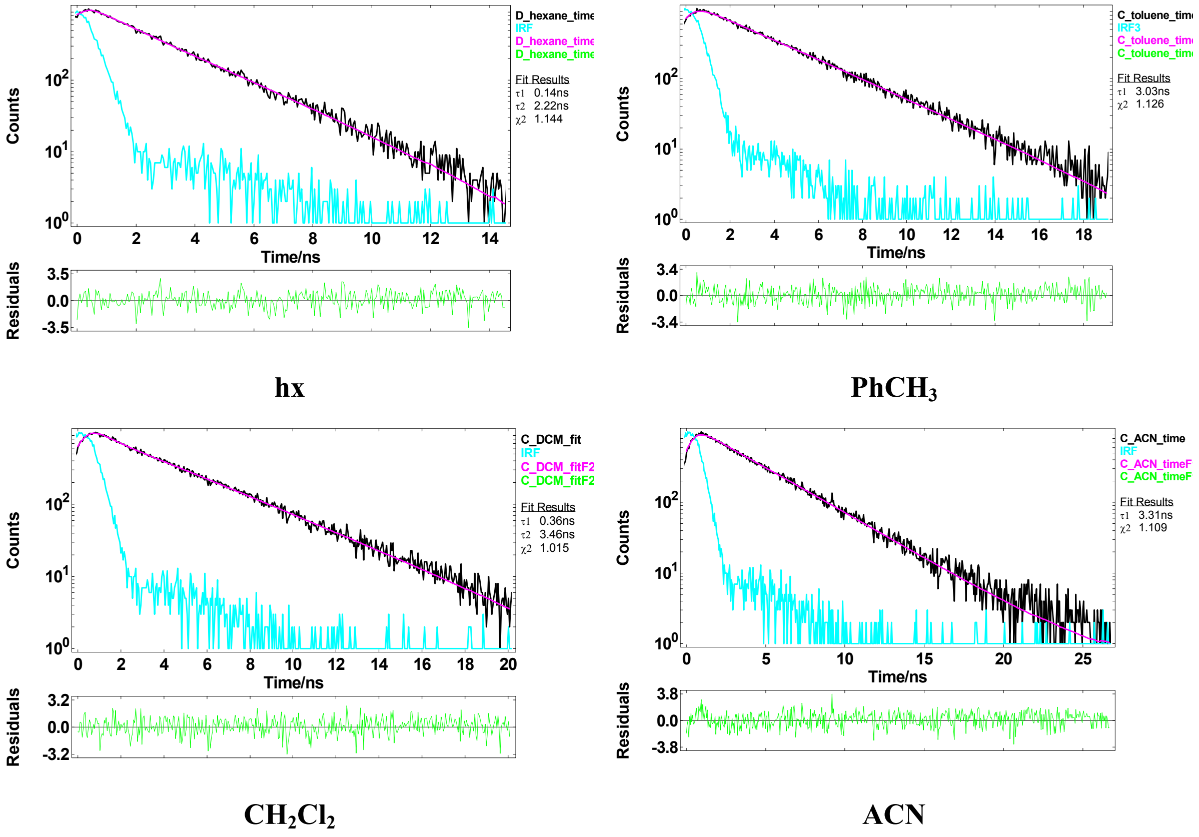

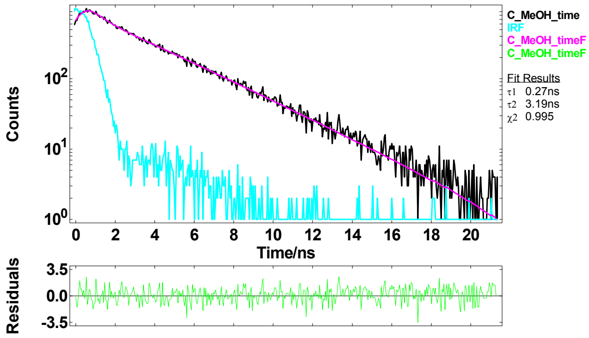

MeOH

Figure S6. Fluorescence decays of **4c** recorded in different solvents.

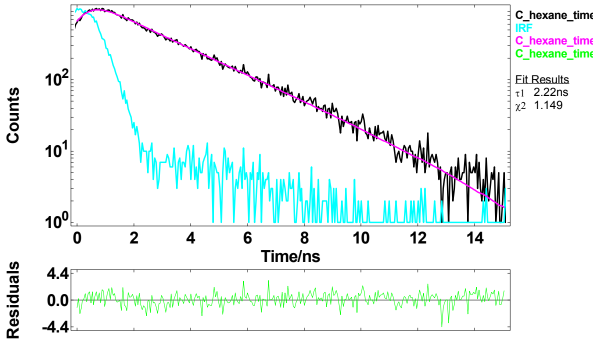

hx

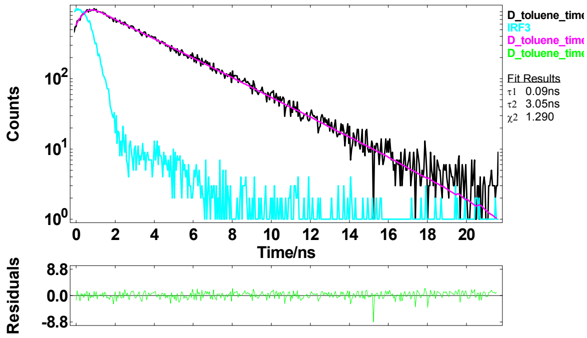

$PhCH_3$

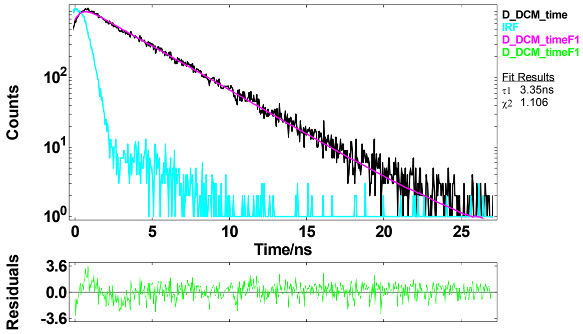

CH_2Cl_2

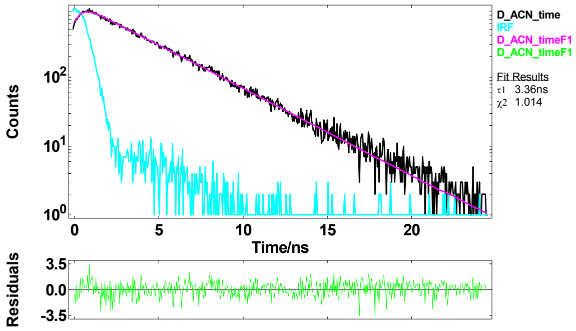

ACN

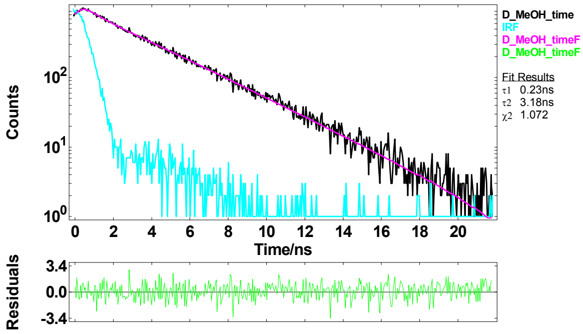

MeOH

Figure S7. Fluorescence decays of **4d** recorded in different solvents.

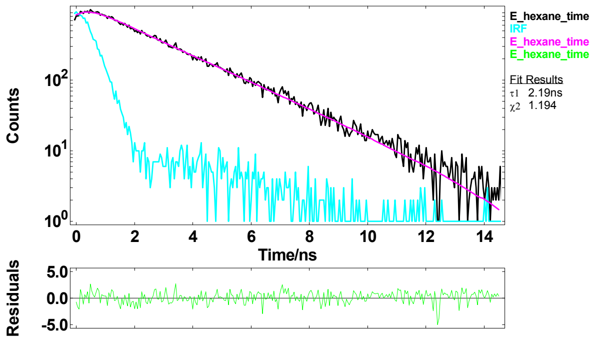

hx

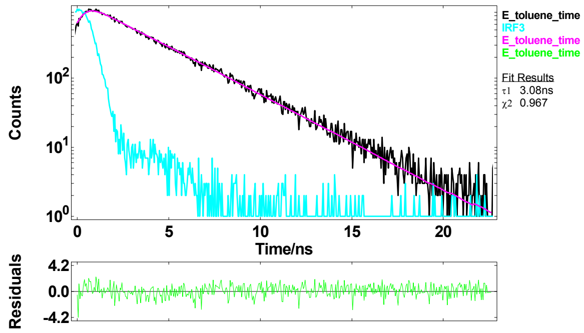

$PhCH_3$

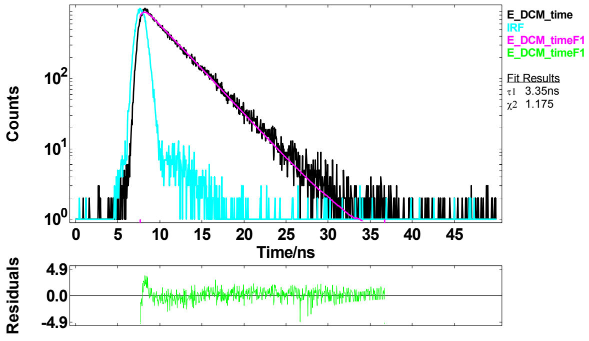

CH_2Cl_2

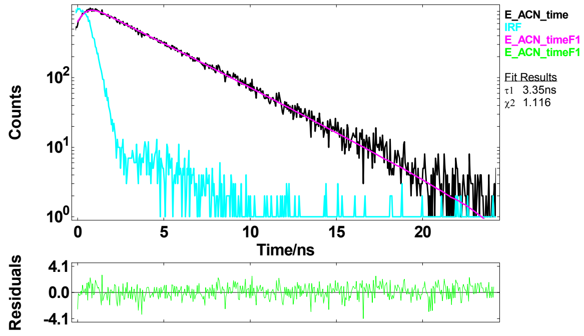

ACN

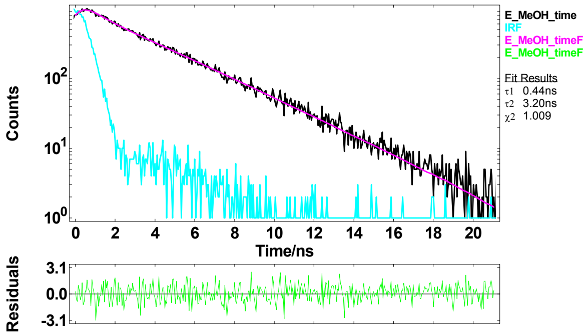

MeOH

Figure S8. Fluorescence decays of **4e** recorded in different solvents.

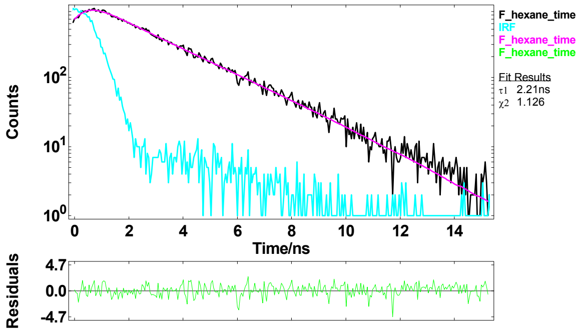

hx

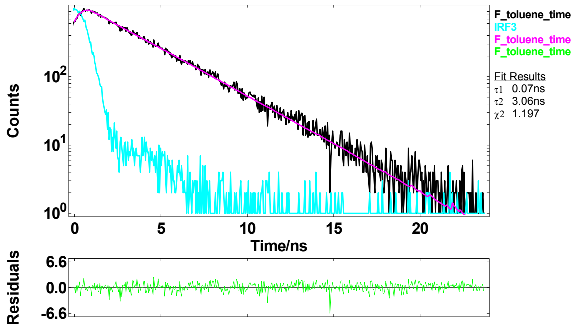

$PhCH_3$

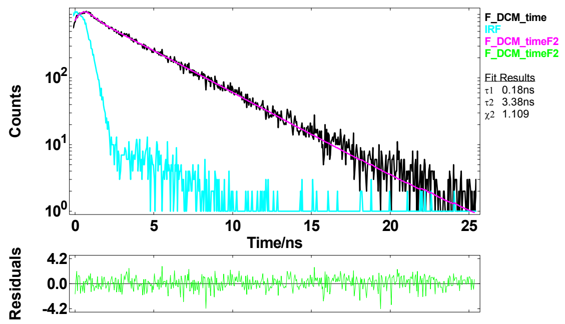

CH_2Cl_2

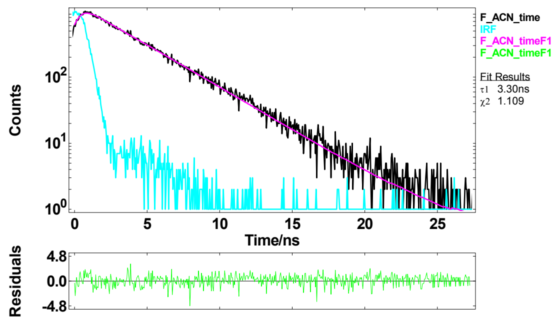

ACN

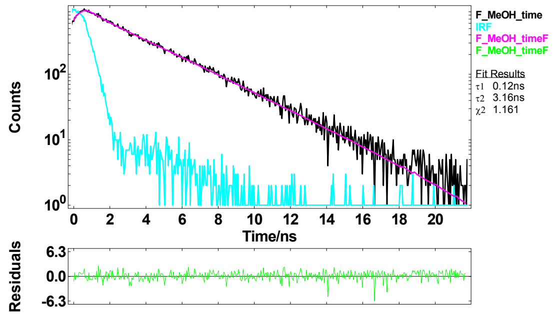

MeOH

Figure S9. Fluorescence decays of **4f** recorded in different solvents.

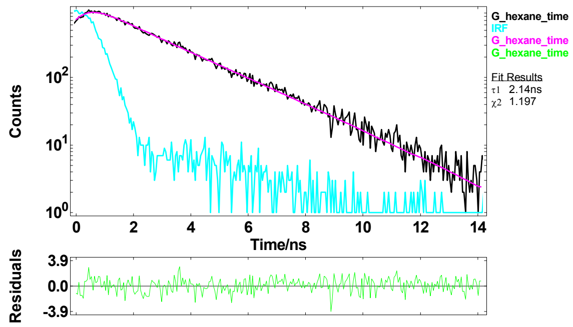

hx

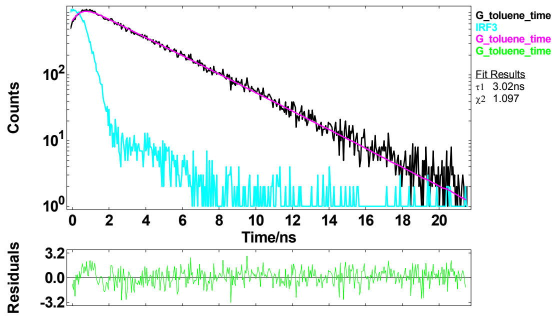

$PhCH_3$

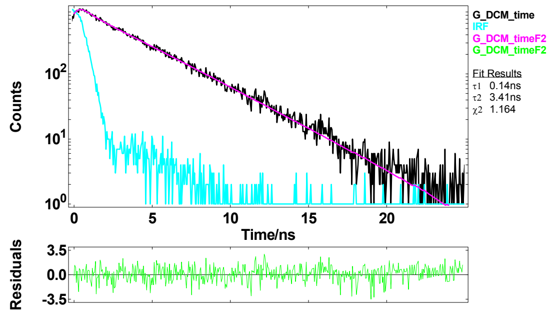

CH_2Cl_2

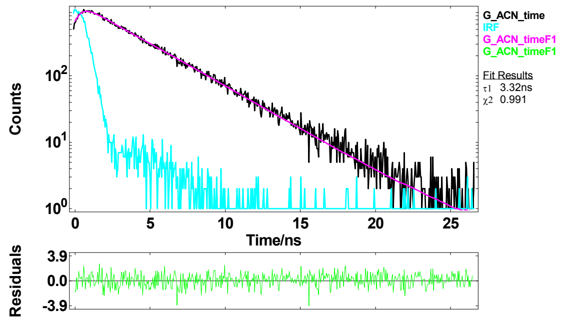

ACN

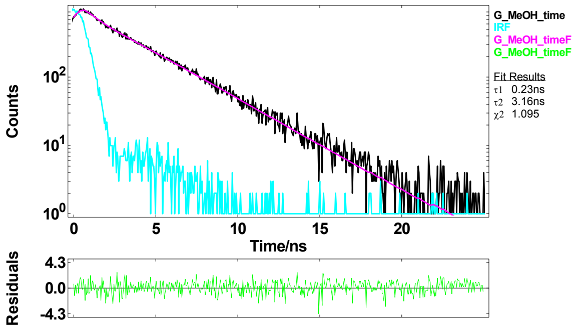

MeOH

Figure S10. Fluorescence decays of **4g** recorded in different solvents.

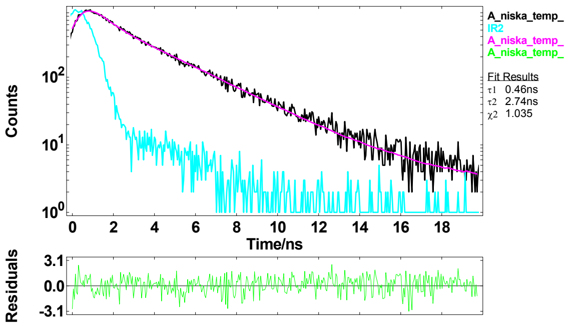

4a

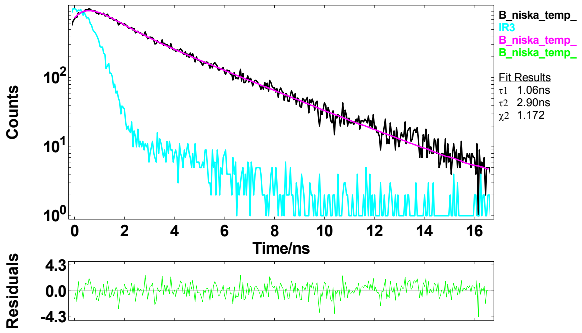

4b

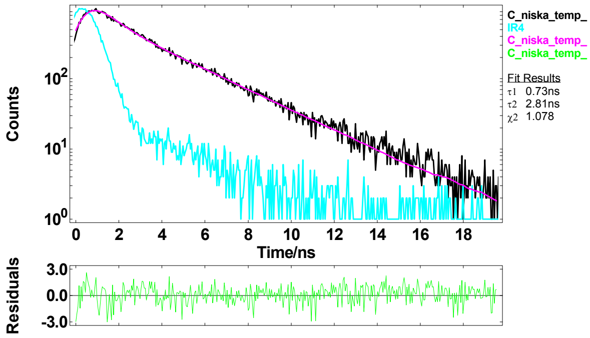

4c

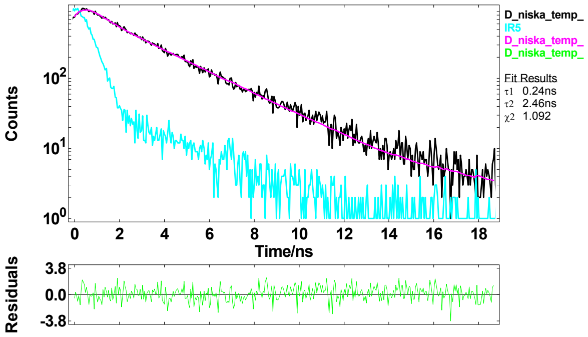

4d

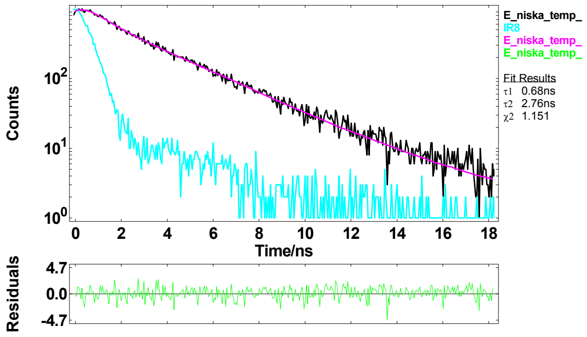

4e

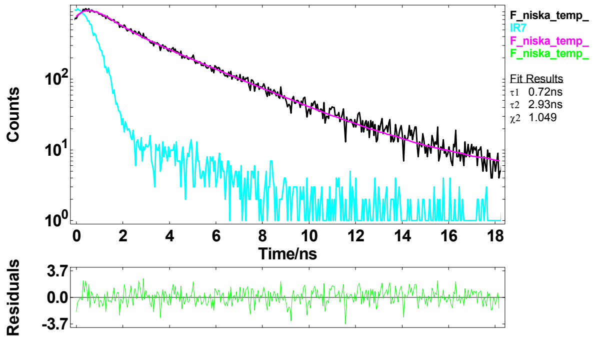

4f

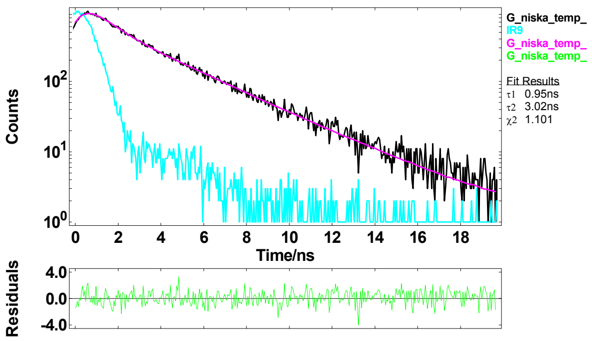

4g

Figure S11. Fluorescence decays of solutions **4a-4g** in EtOH/MeOH (4:1, *v/v*) recorded at low-temperature 77K.

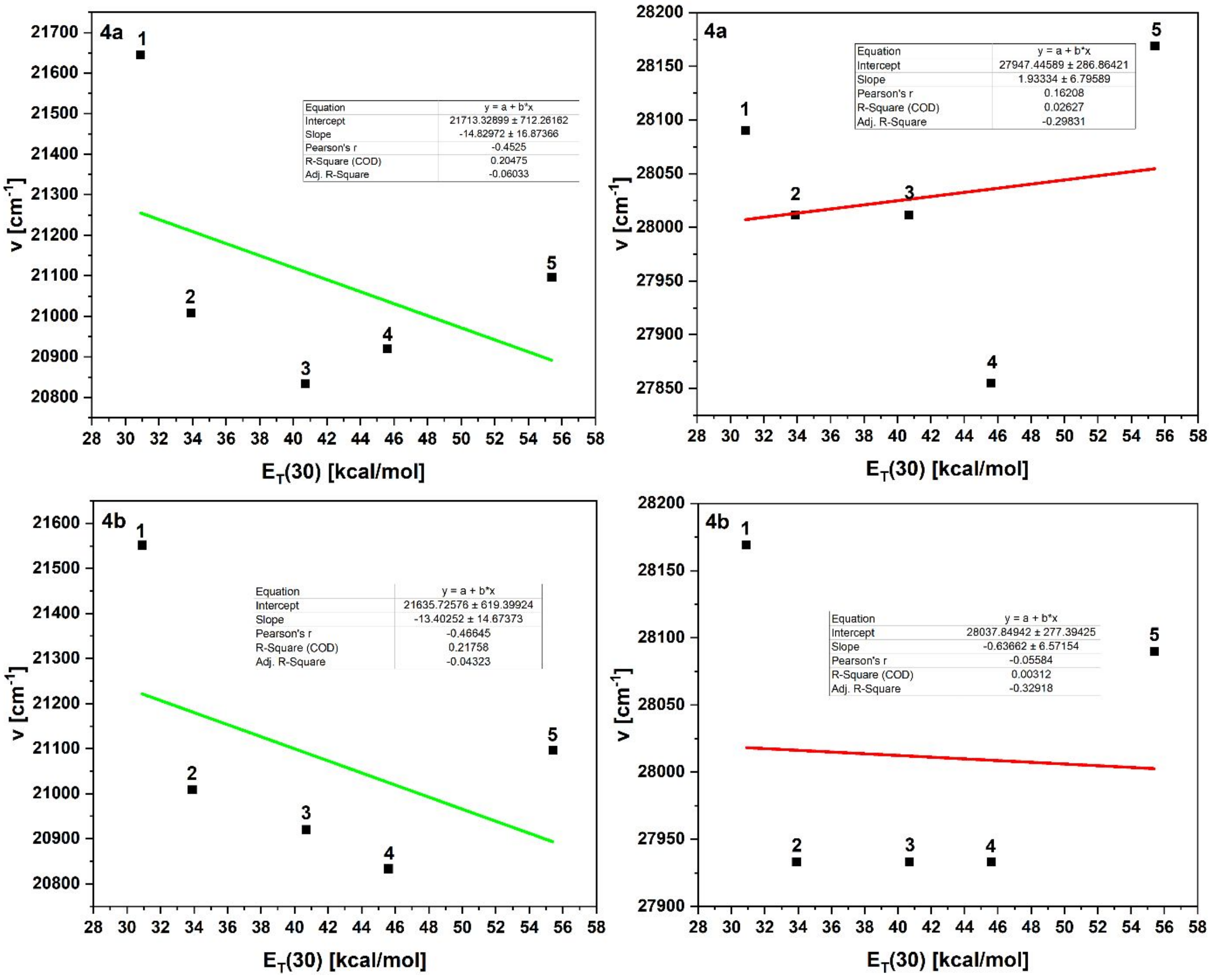

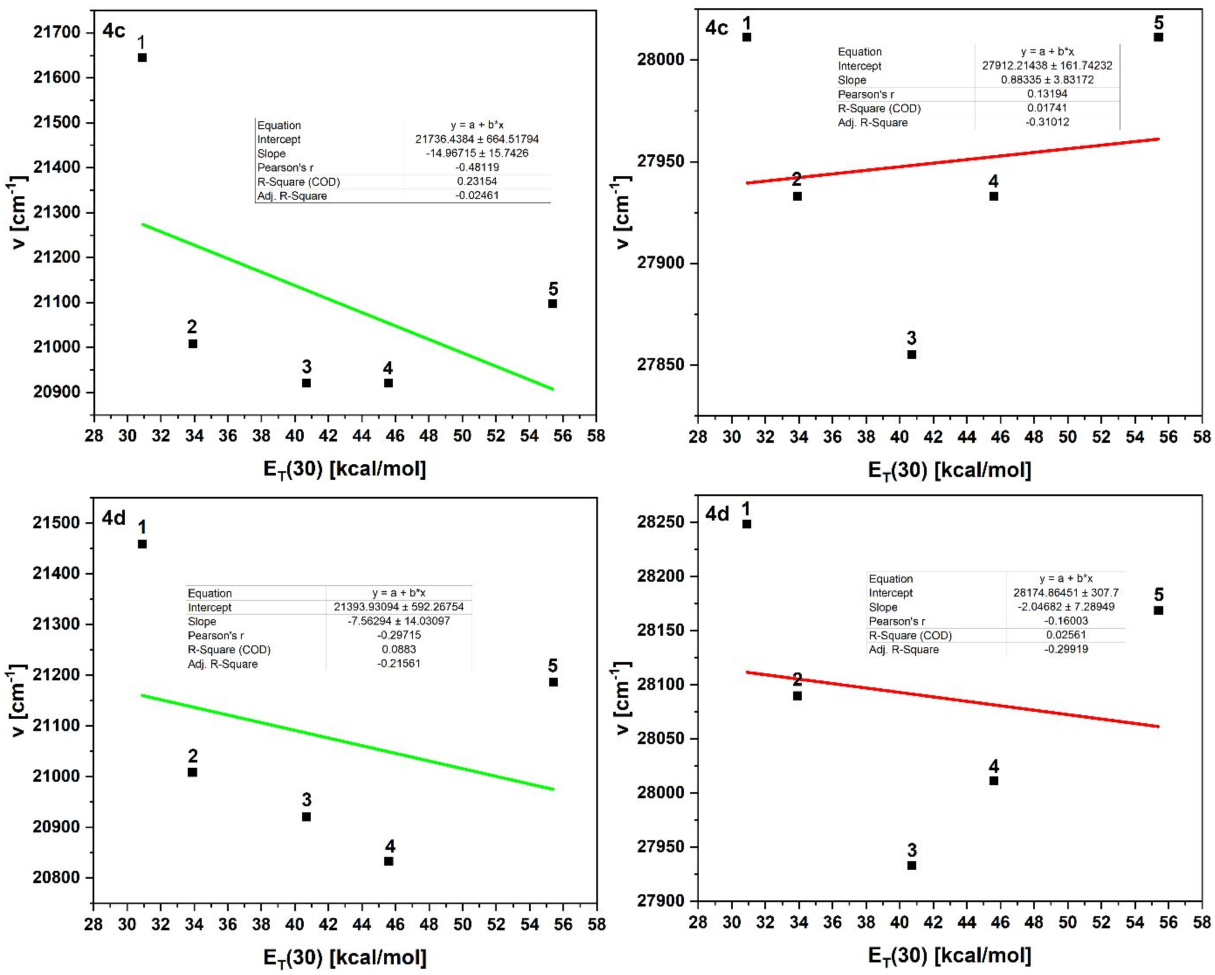
4c
Equation y = a + b*x
Intercept 21736.4384 ± 664.51794
Slope -14.96715 ± 15.7426
Pearson's r -0.48119
R-Square (COD) 0.23154
Adj. R-Square -0.02461
v [cm-1]
ET(30) [kcal/mol]
4c
Equation y = a + b*x
Intercept 27912.21438 ± 161.74232
Slope 0.88335 ± 3.83172
Pearson's r 0.13194
R-Square (COD) 0.01741
Adj. R-Square -0.31012
v [cm-1]
ET(30) [kcal/mol]
4d
Equation y = a + b*x
Intercept 21393.93094 ± 592.26754
Slope -7.56294 ± 14.03097
Pearson's r -0.29715
R-Square (COD) 0.0883
Adj. R-Square -0.21561
v [cm-1]
ET(30) [kcal/mol]
4d
Equation y = a + b*x
Intercept 28174.86451 ± 307.7
Slope -2.04682 ± 7.28949
Pearson's r -0.16003
R-Square (COD) 0.02561
Adj. R-Square -0.29919
v [cm-1]
ET(30) [kcal/mol]

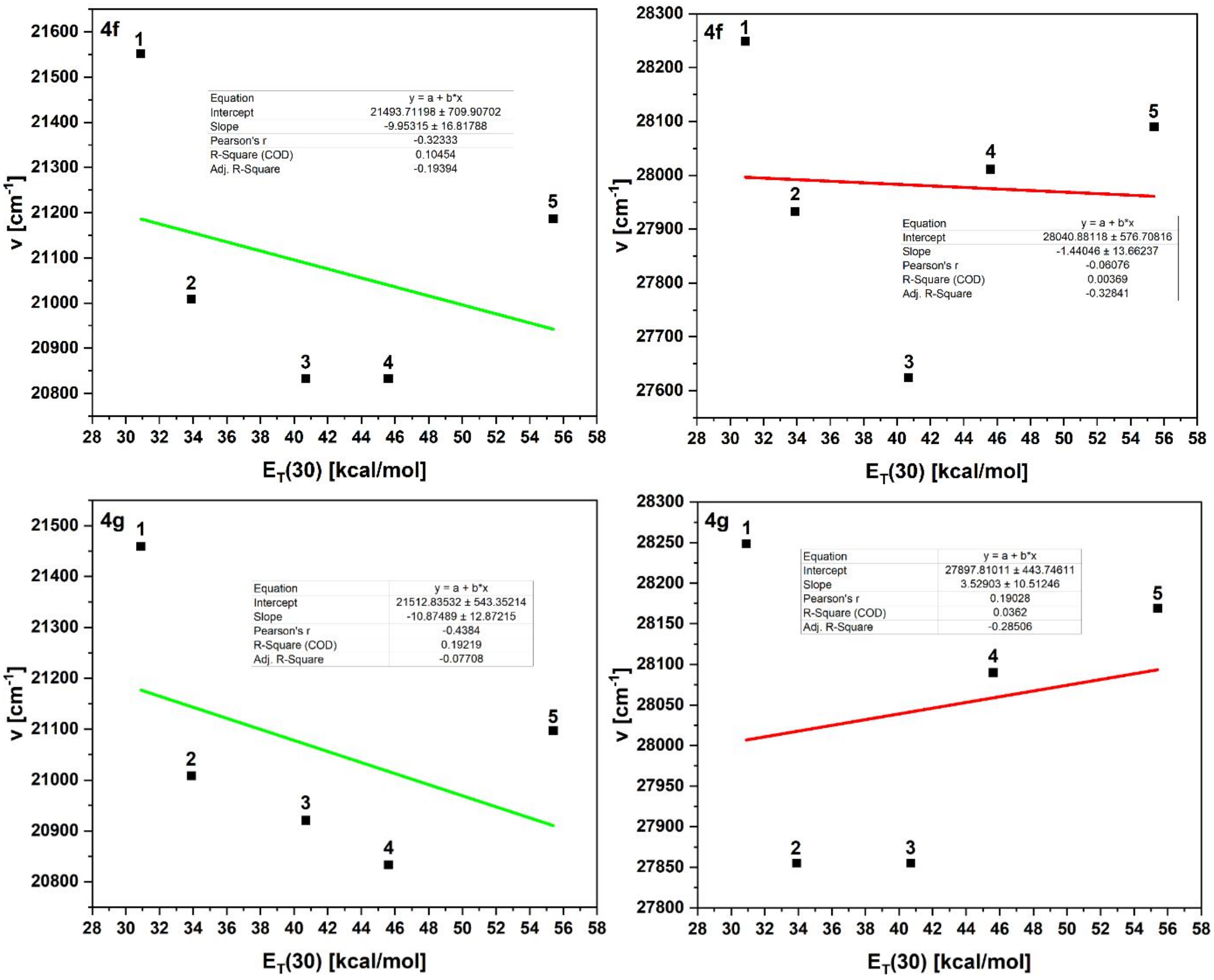

Figure S12. Dependence of υ_{em} (green line) and υ_{abs} (red line) for **4a-4d**, and **4f-4g**. on solvent polarity ET (30) value. Solvents: (1) Hexane, (2) Toluene, (3) Dichloromethane, (4) Methanol, (5) Acetonitrile.

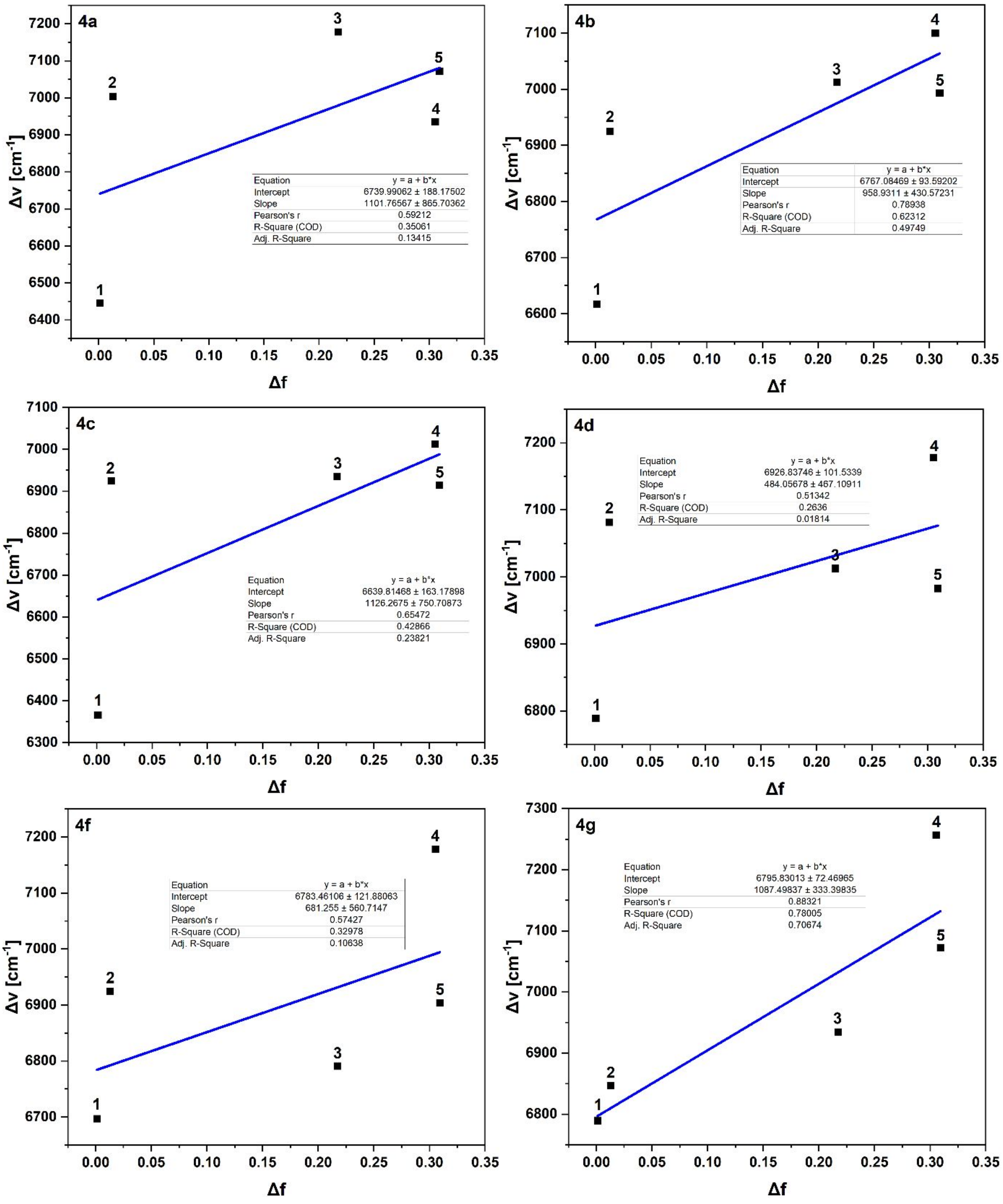

Figure S13. Stokes shift as a function of the solvent orientation polarizability (Δf) for **4a-4d**, and **4f-4g**. Solvents: (1) Hexane, (2) Toluene, (3) Dichloromethane, (4) Methanol, (5) Acetonitrile.

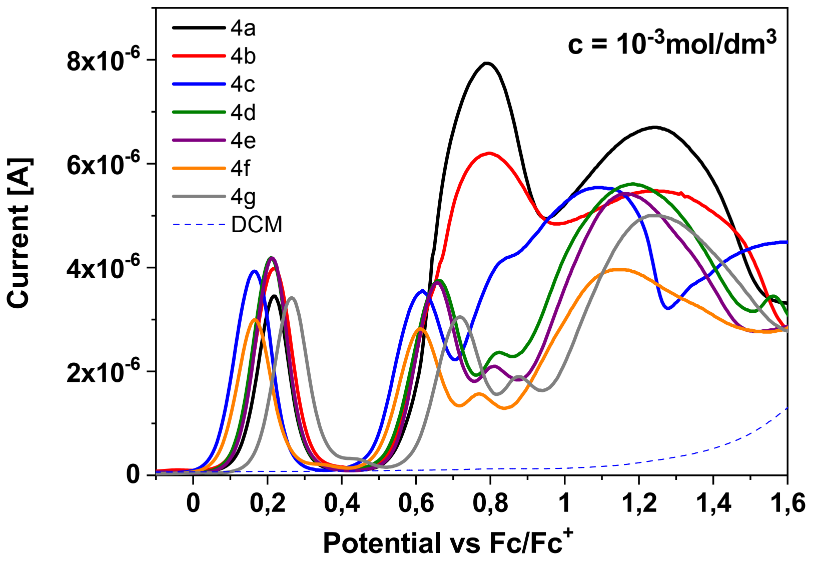

Figure S14. The voltammograms of **4a-4g** in DPV method (v = 0.01 V/s, Pt, 0.1 mol/dm^3 Bu_4NPF_6 in DCM).

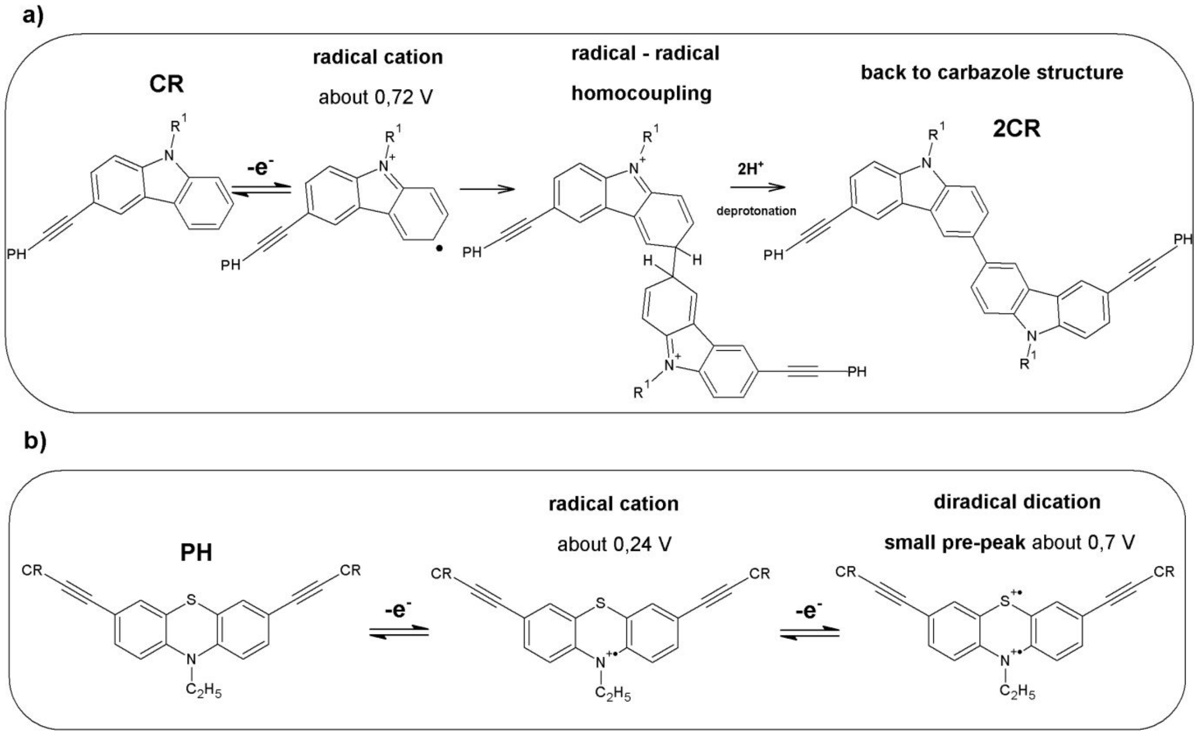

Figure S15. The proposed mechanism for the electropolymerization of carbazole fragments of the molecule involves the formation of a phenothiazine dication diradical.

Table S7. Cytotoxicity of **4a-4g** and doxorubicin (DOX) against cancer and normal cells.

Compound/ Cell lines	IC$_{50}$ [µM]			
	MCF-7	HCT 116	PANC-1	NHDF
**4a**	>25	>25	>25	>25
**4b**	>25	>25	>25	>25
**4c**	>25	>25	>25	>25
**4d**	>25	>25	>25	>25
**4e**	>25	>25	>25	>25
**4f**	>25	>25	>25	>25
**4g**	>25	>25	>25	>25
**DOX**	0.40 ± 0.04	0.34 ± 0.04	0.73 ± 0.09	0.14 ± 0.03

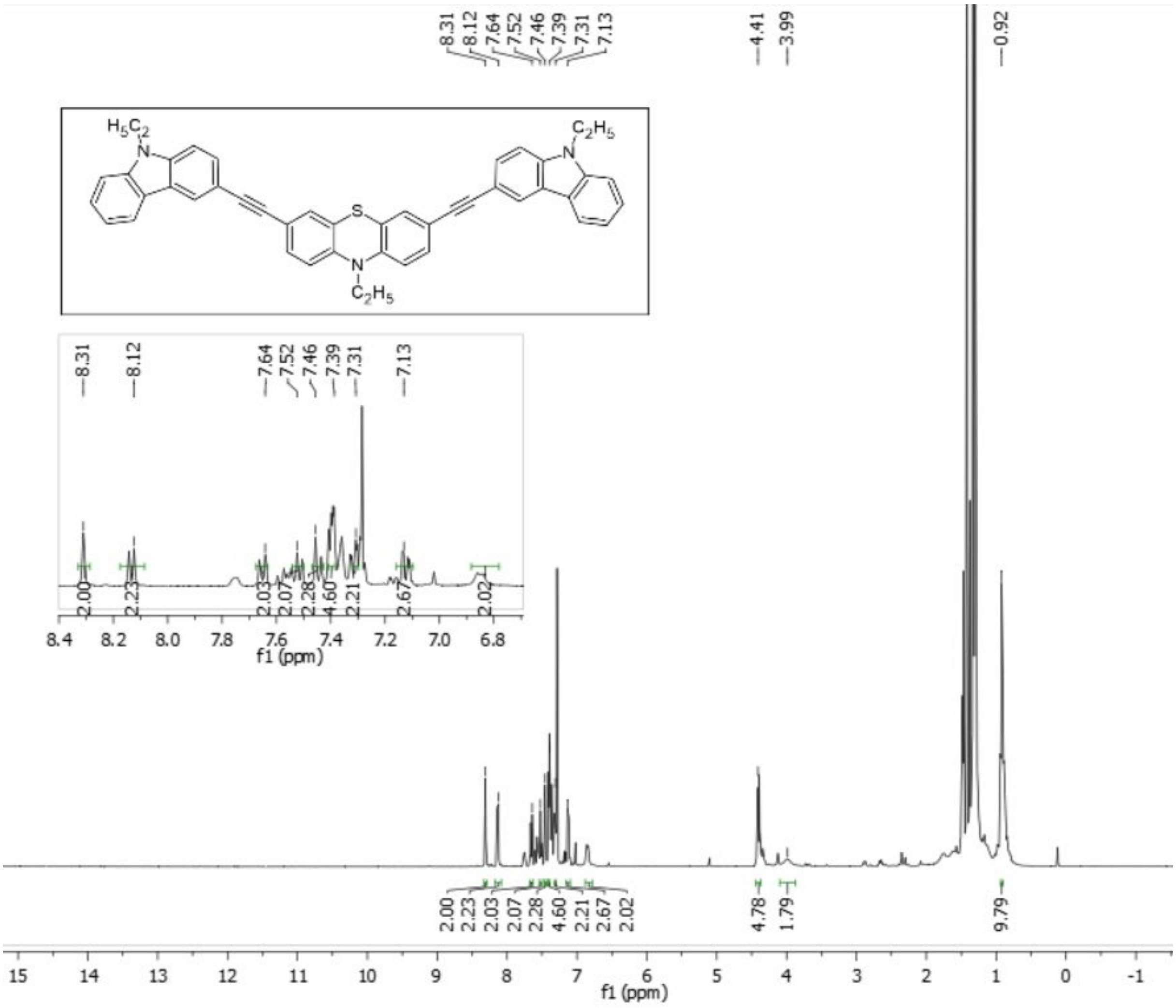

Figure S16. The ^{1}H spectrum of **4a** in $CDCl_3$.

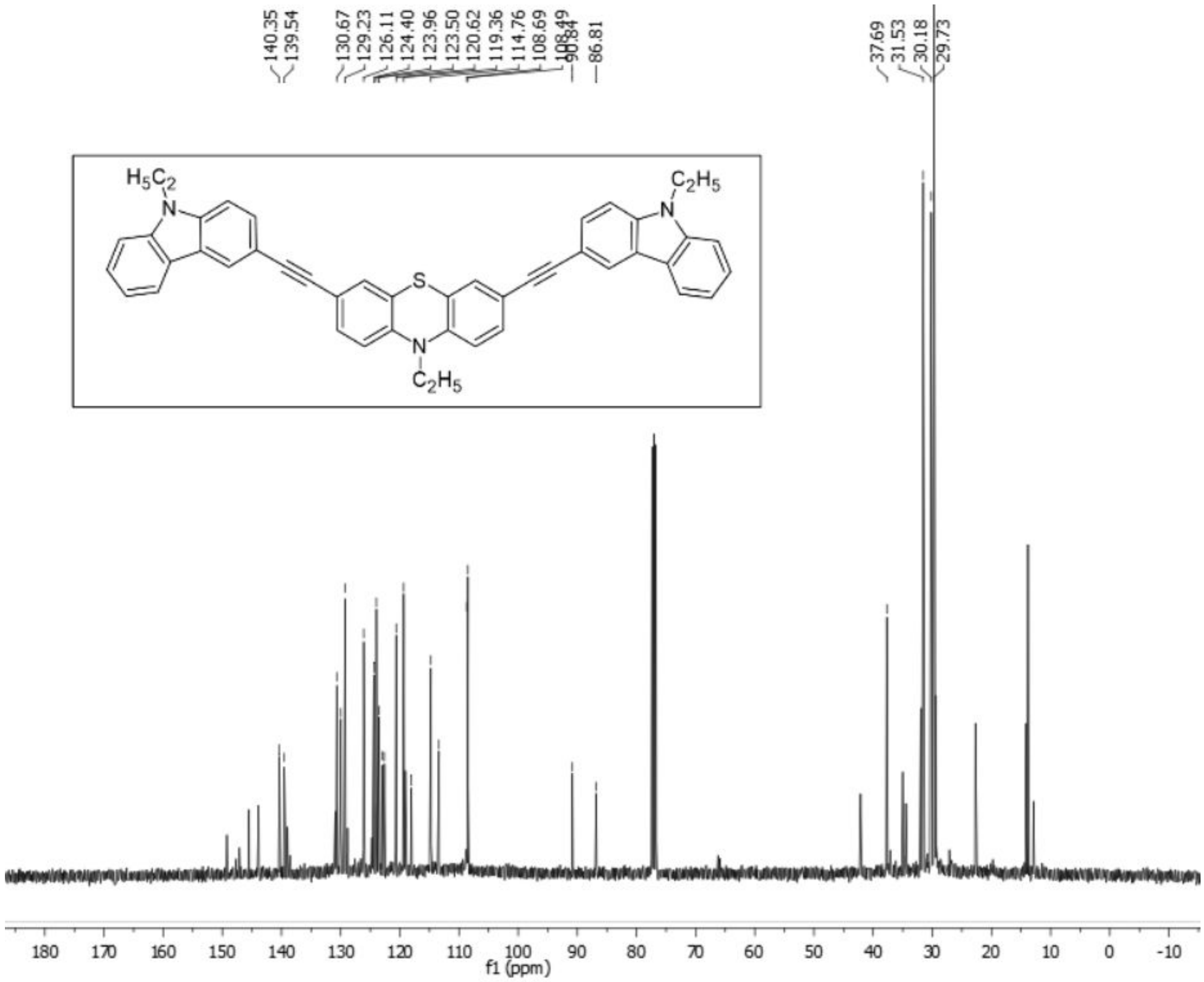

Figure S17. The ^{13}C spectrum of **4a** in $CDCl_3$.

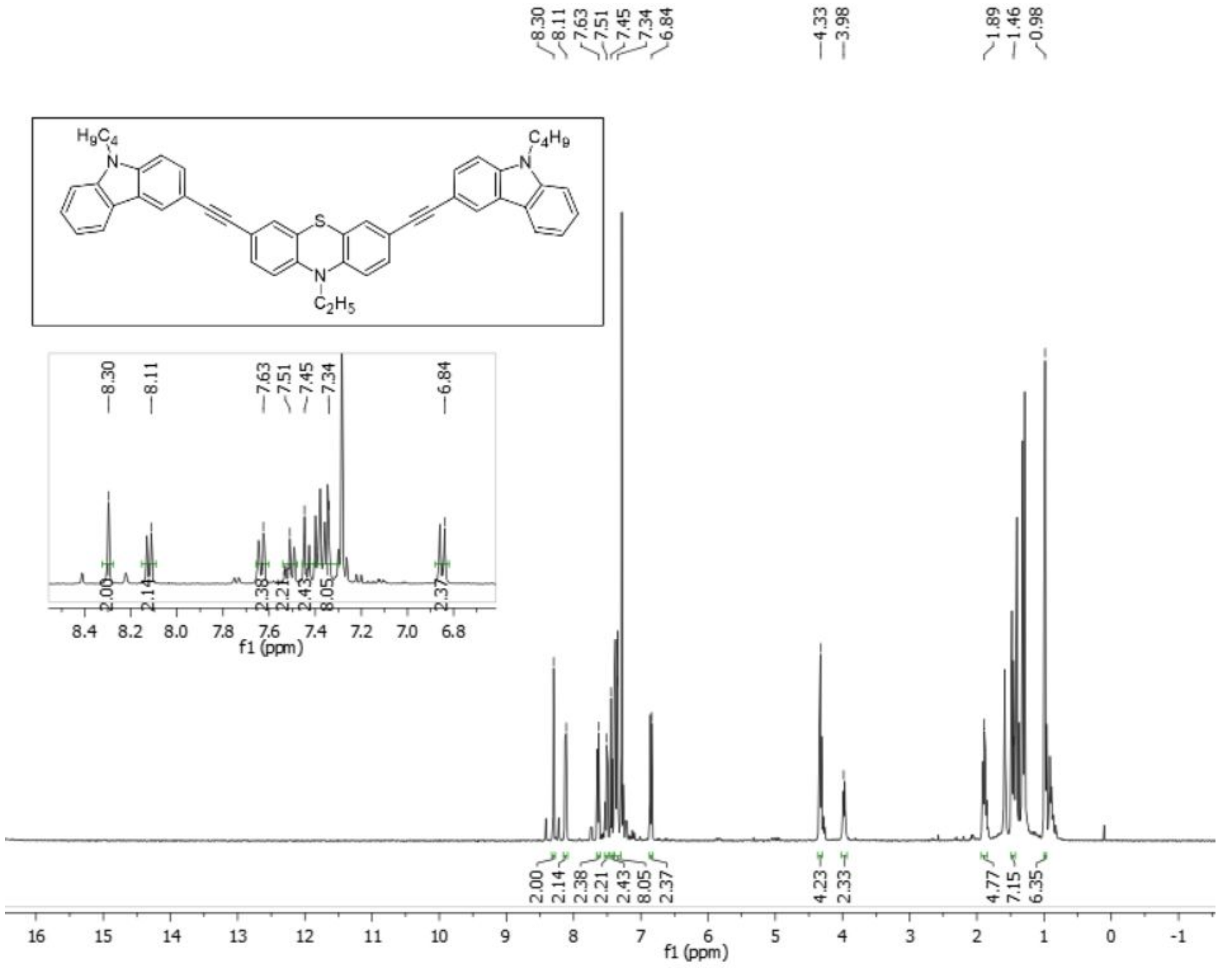

Figure S18. The ^{1}H spectrum of **4b** in $CDCl_3$.

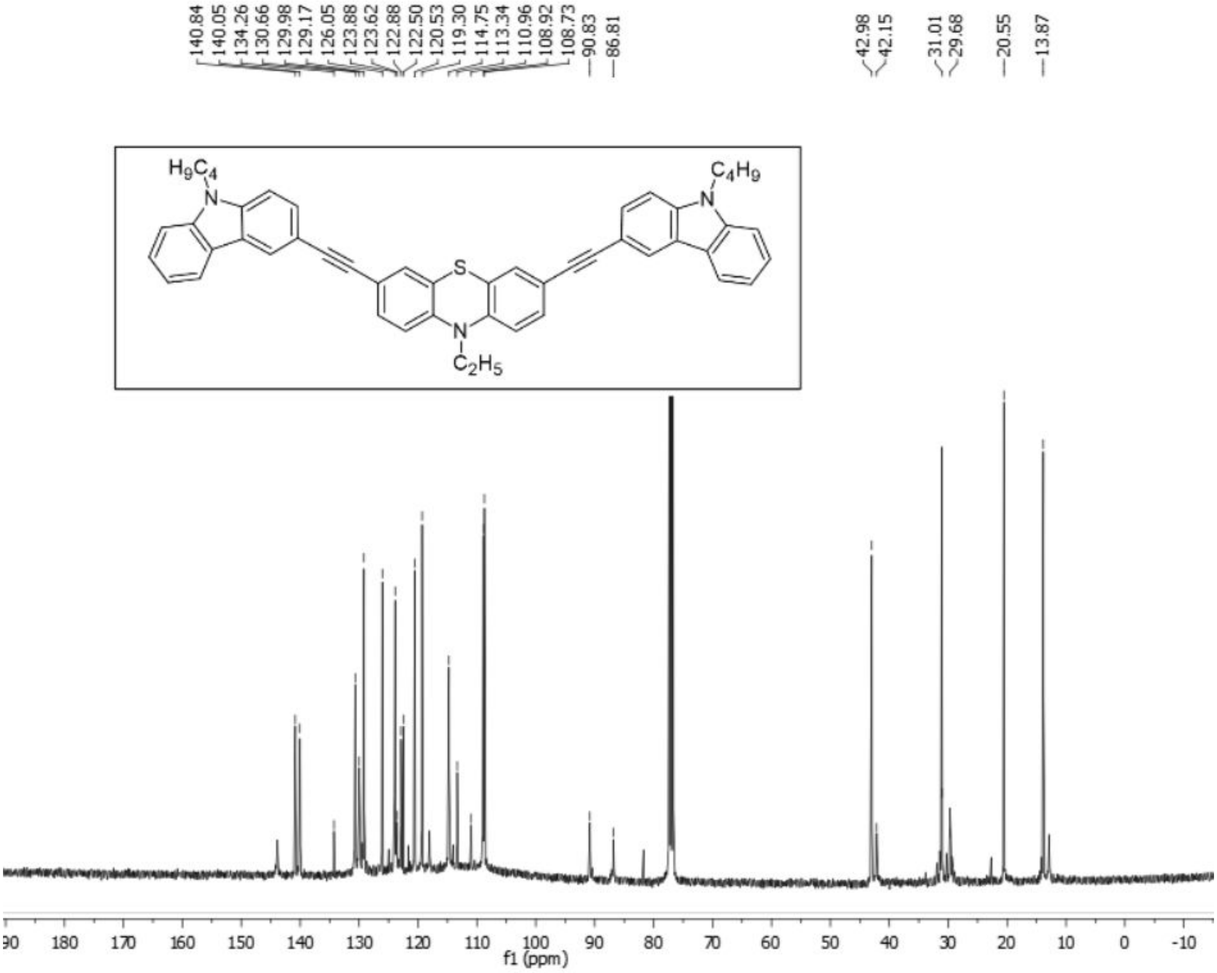

Figure S19. The ^{13}C spectrum of **4b** in $CDCl_3$.

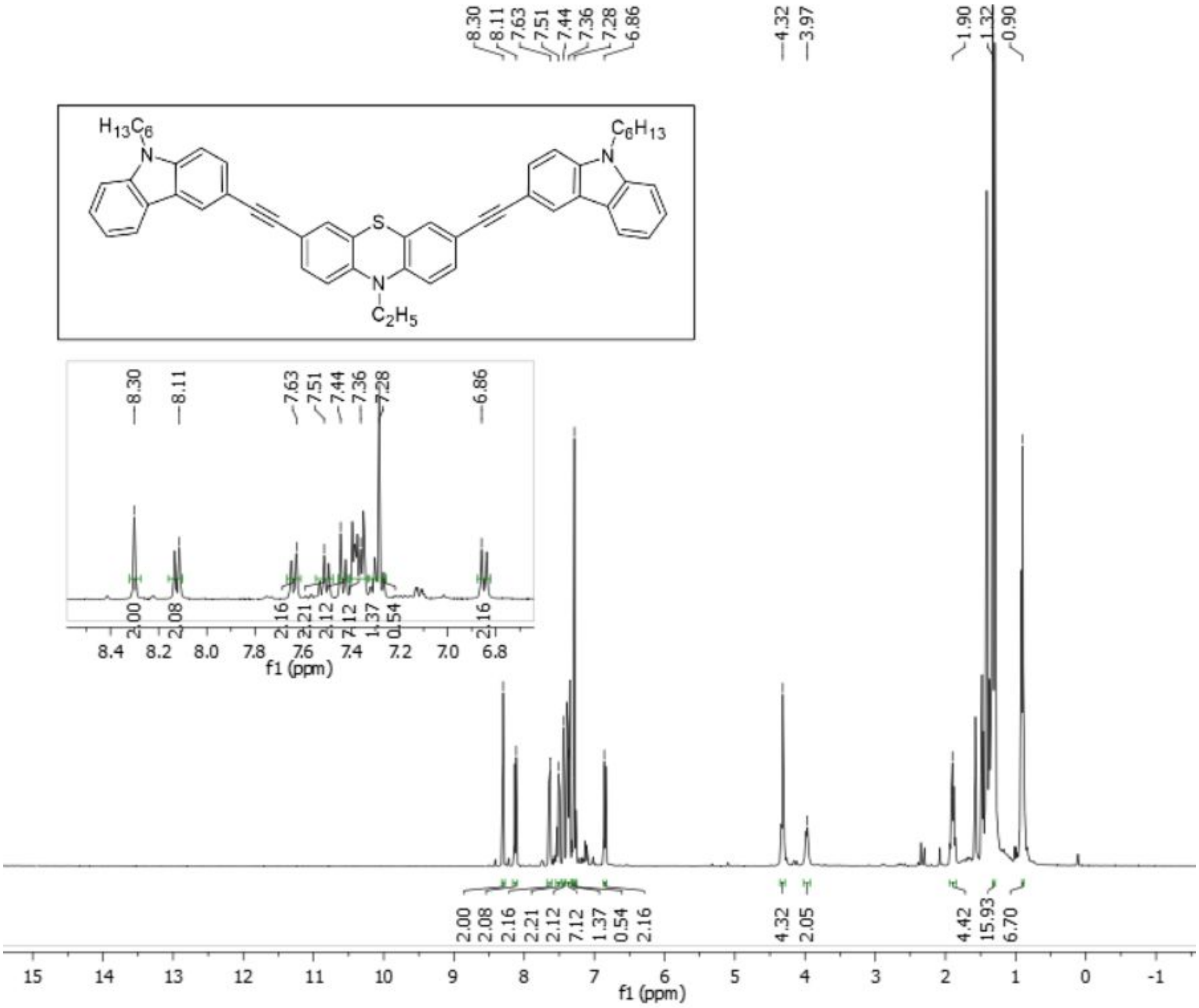

Figure S20. The ^{1}H spectrum of **4c** in $CDCl_3$.

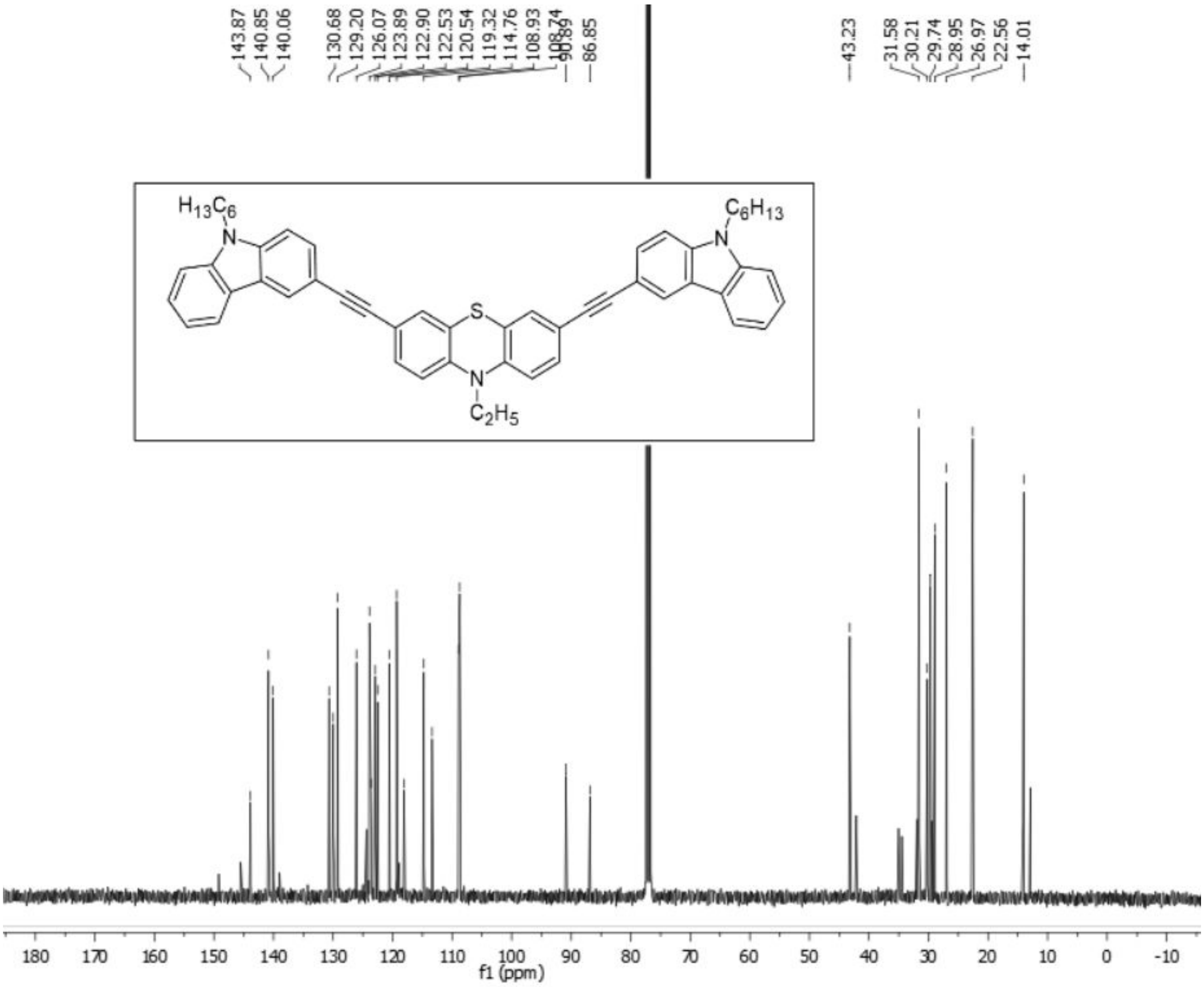

Figure S21. The ^{13}C spectrum of **4c** in $CDCl_3$.

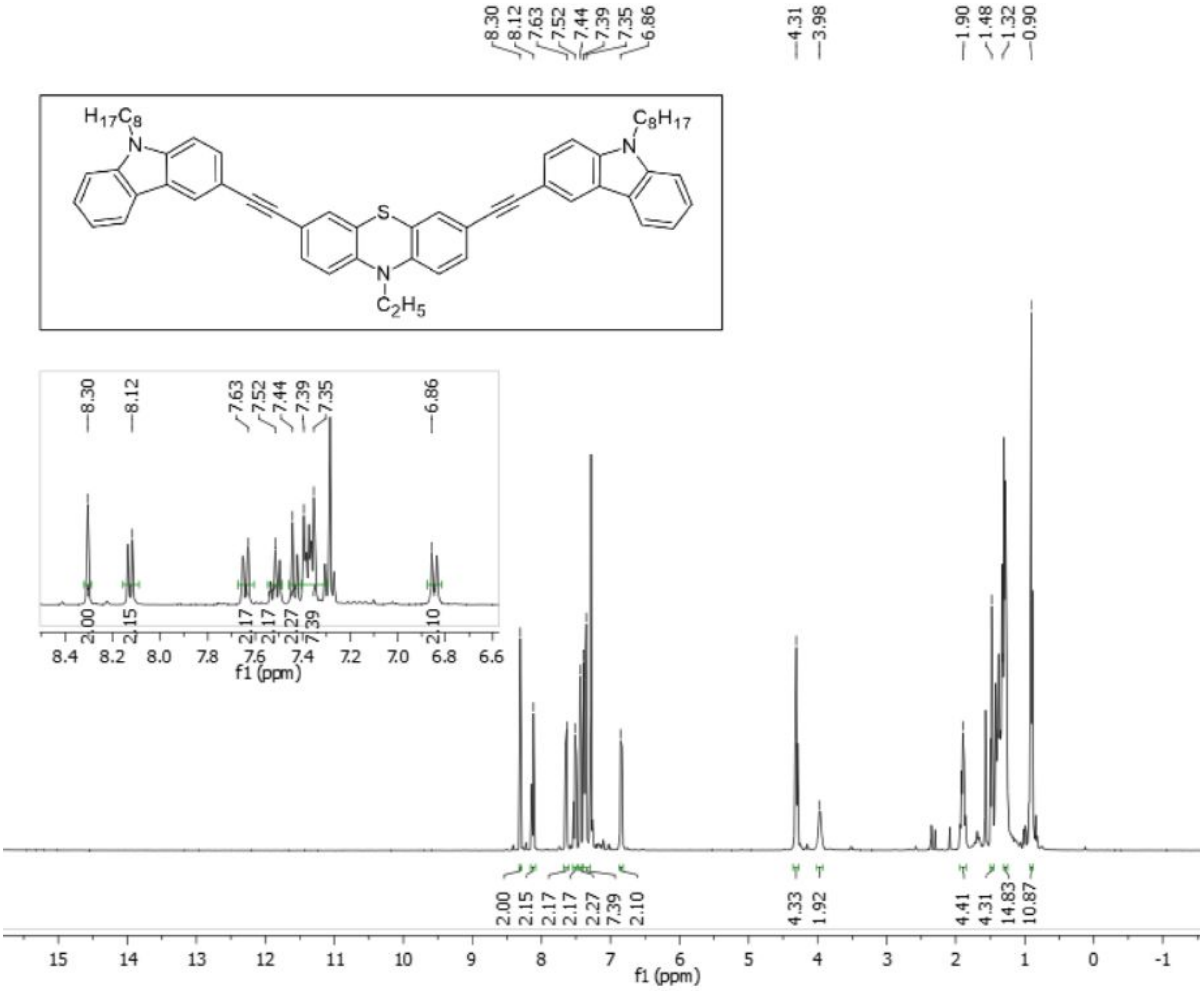

Figure S22. The ^{1}H spectrum of **4d** in $CDCl_3$.

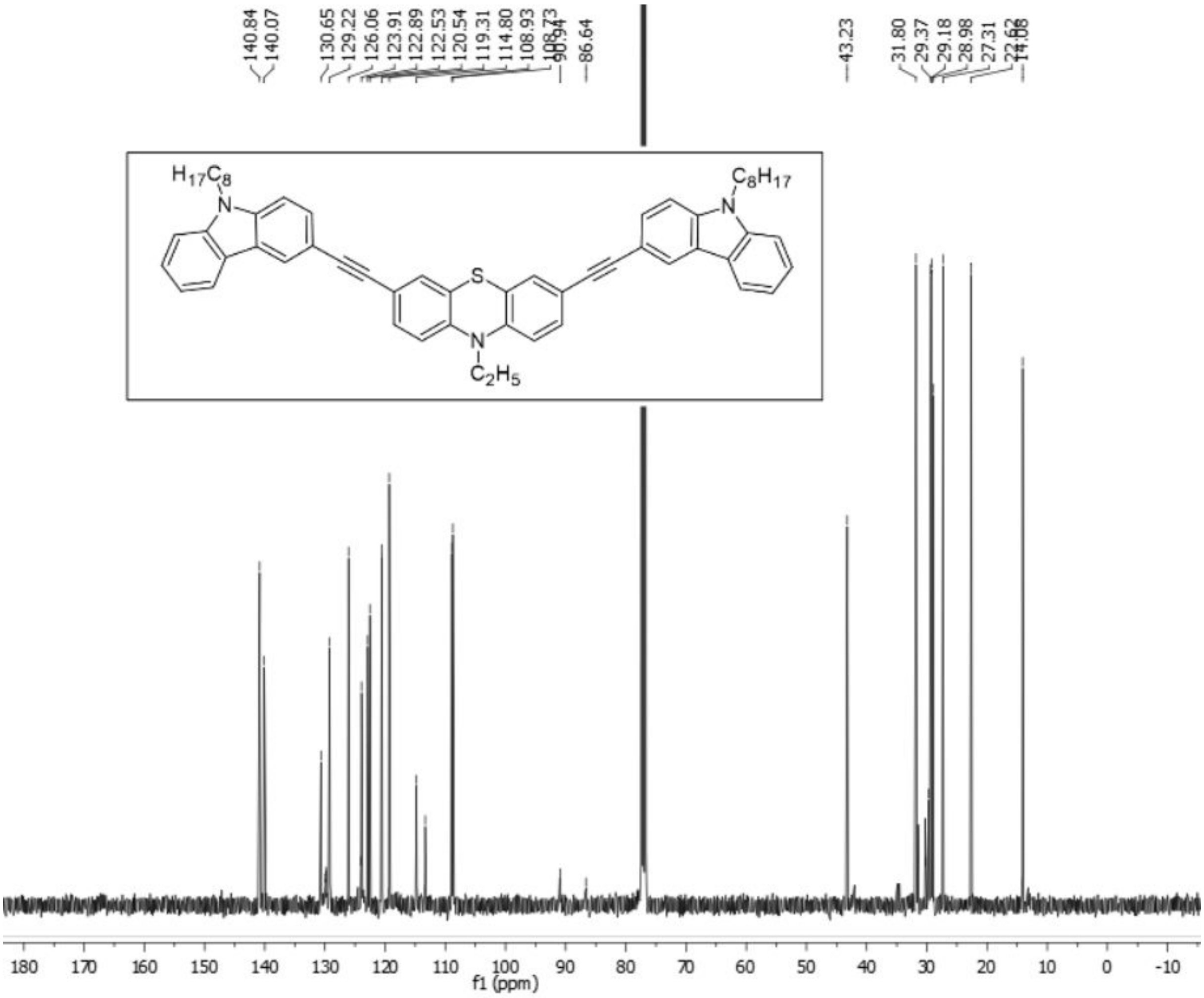

Figure S23. The ^{13}C spectrum of **4d** in $CDCl_3$.

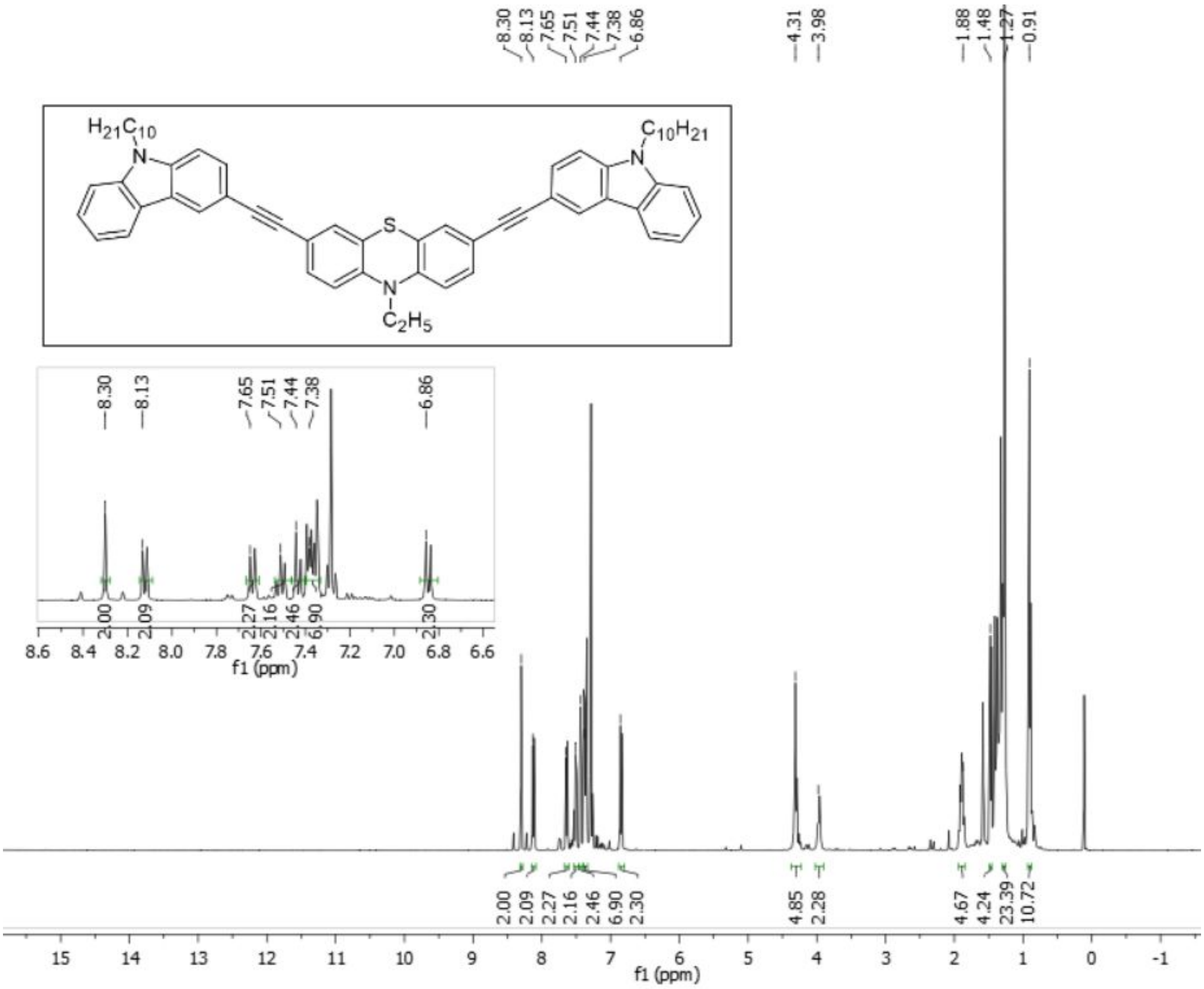

Figure S24. The ^{1}H spectrum of **4e** in $CDCl_3$

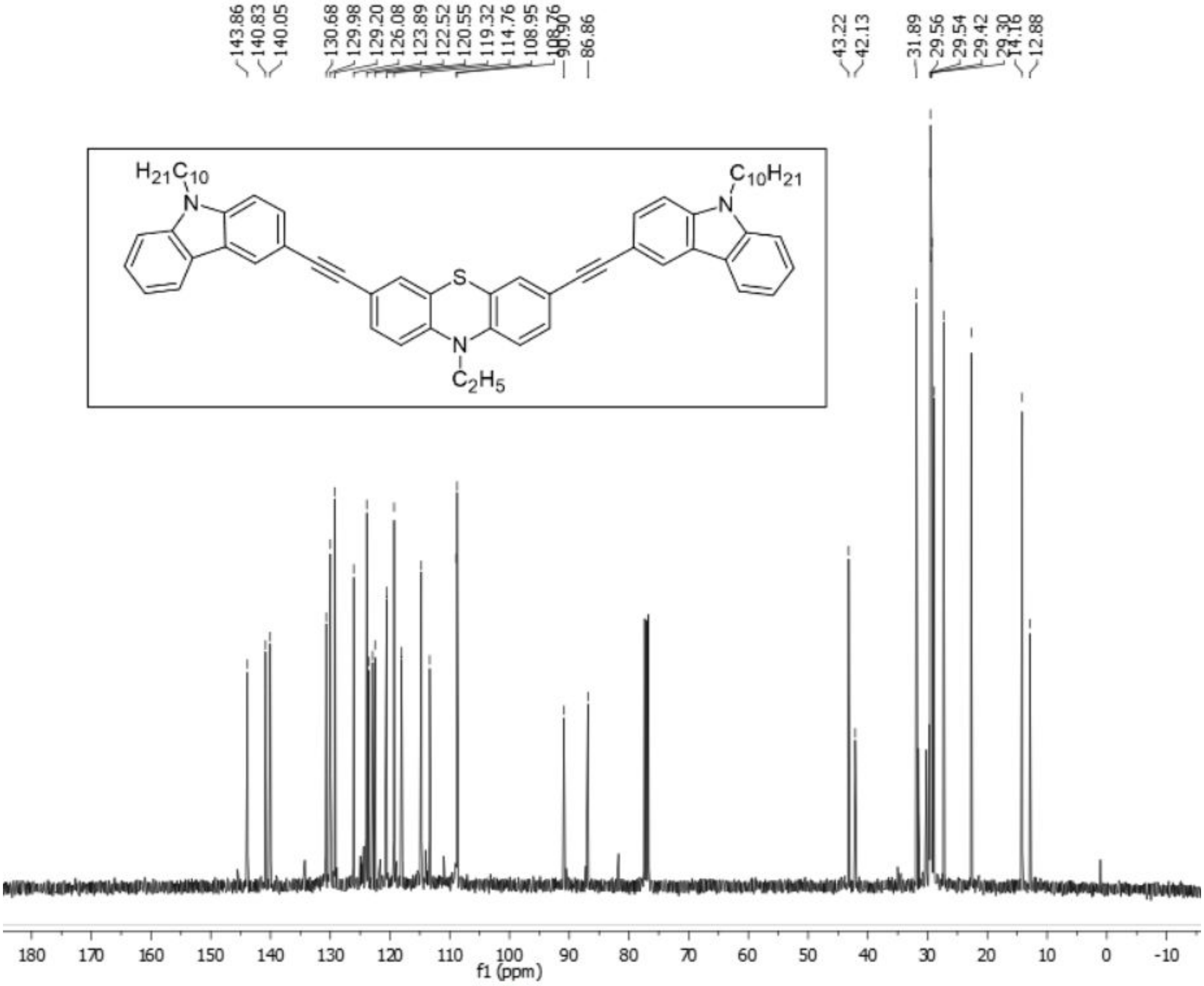

Figure S25. The ^{13}C spectrum of **4e** in $CDCl_3$

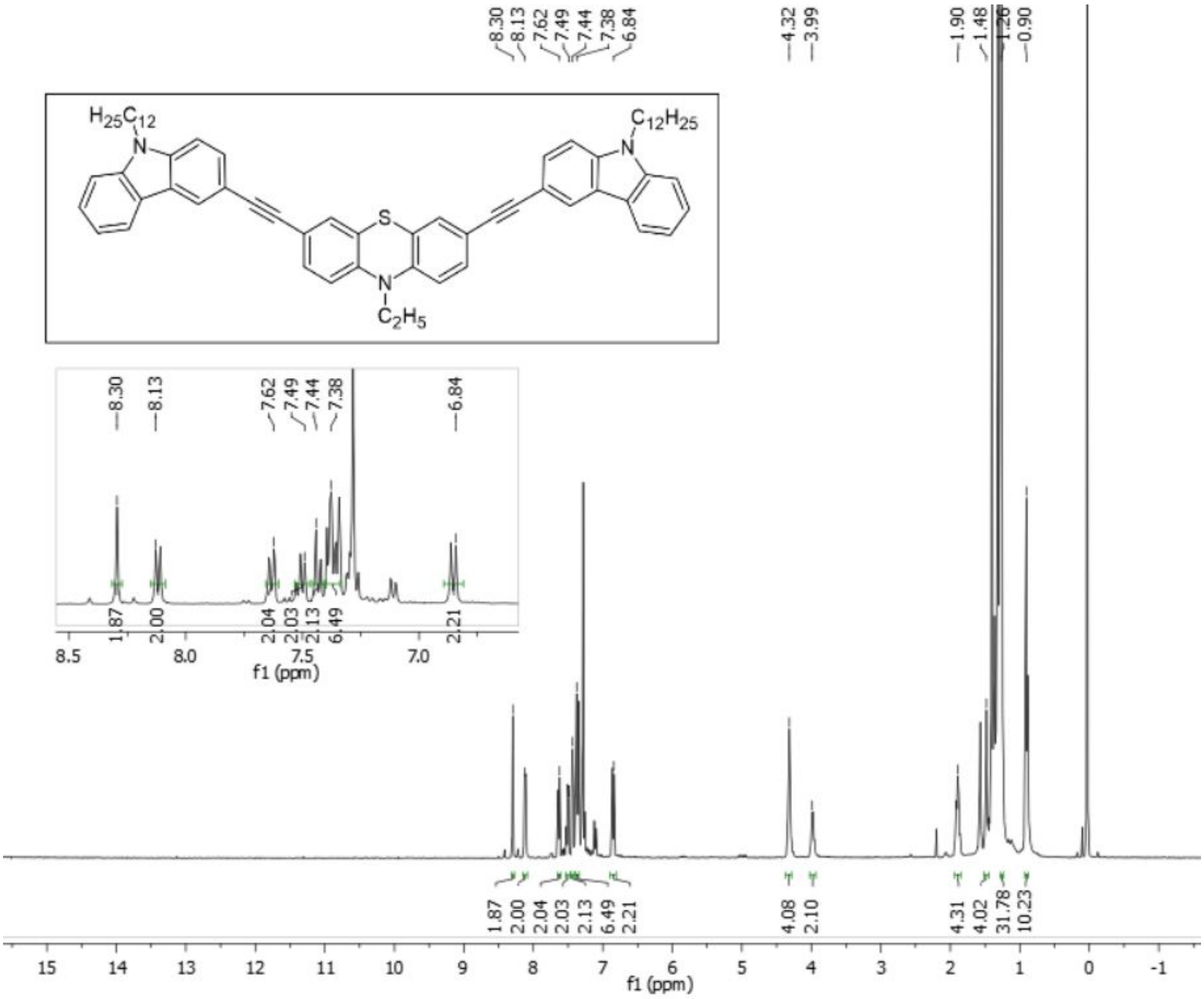

Figure S26. The ^{1}H spectrum of **4f** in $CDCl_3$.

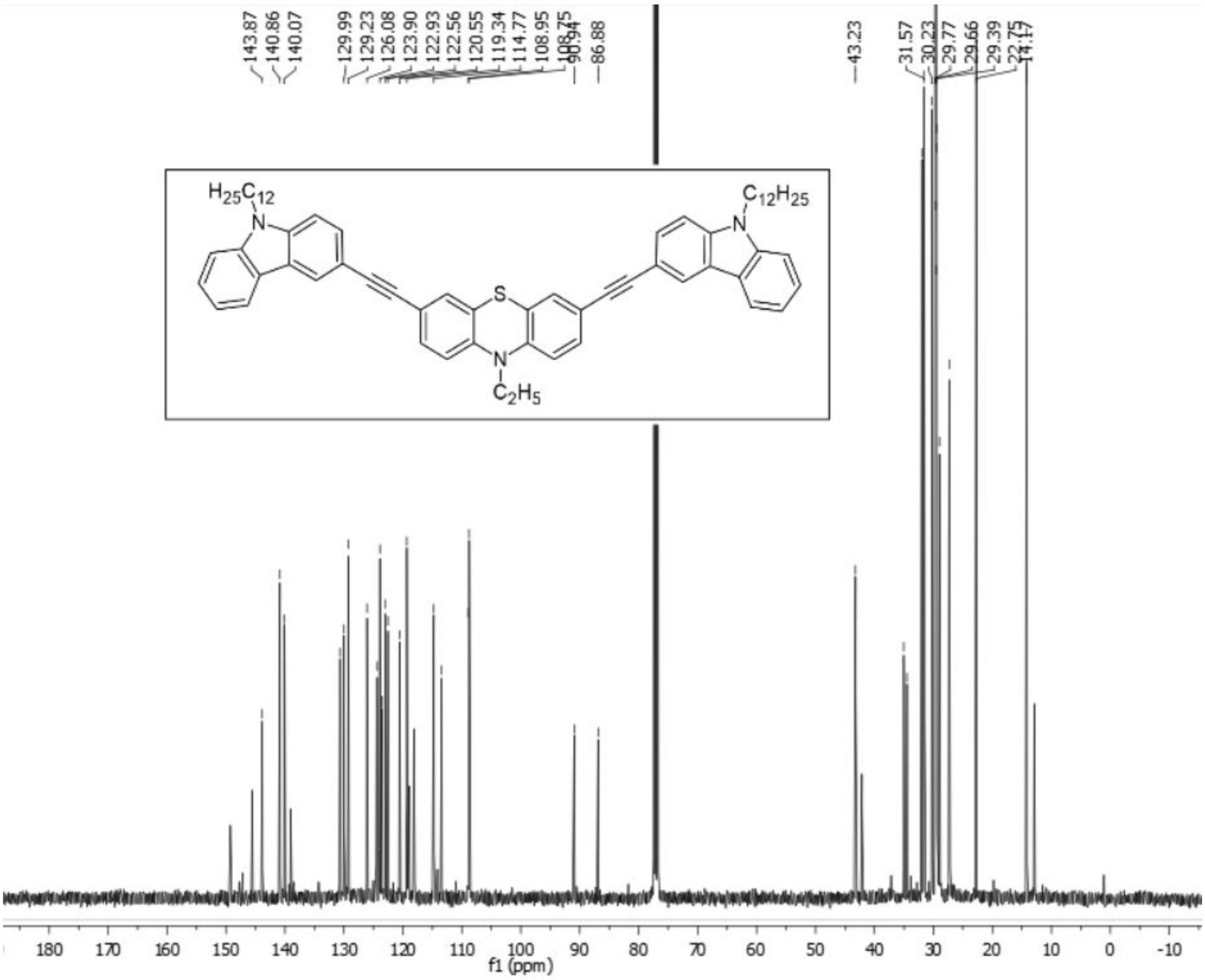

Figure S27. The ^{13}C spectrum of **4f** in $CDCl_3$.

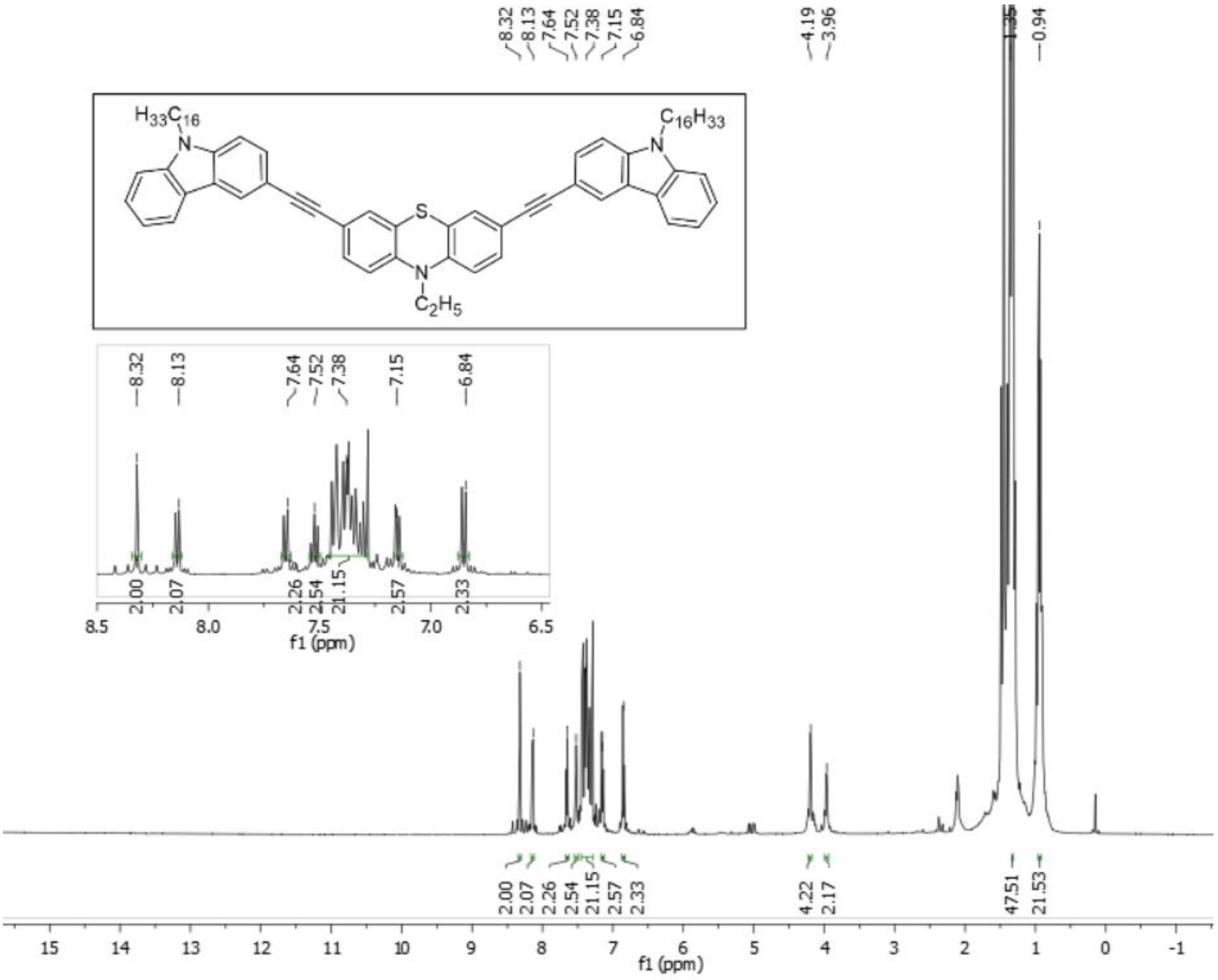

Figure S28. The ^{1}H spectrum of **4g** in $CDCl_3$.

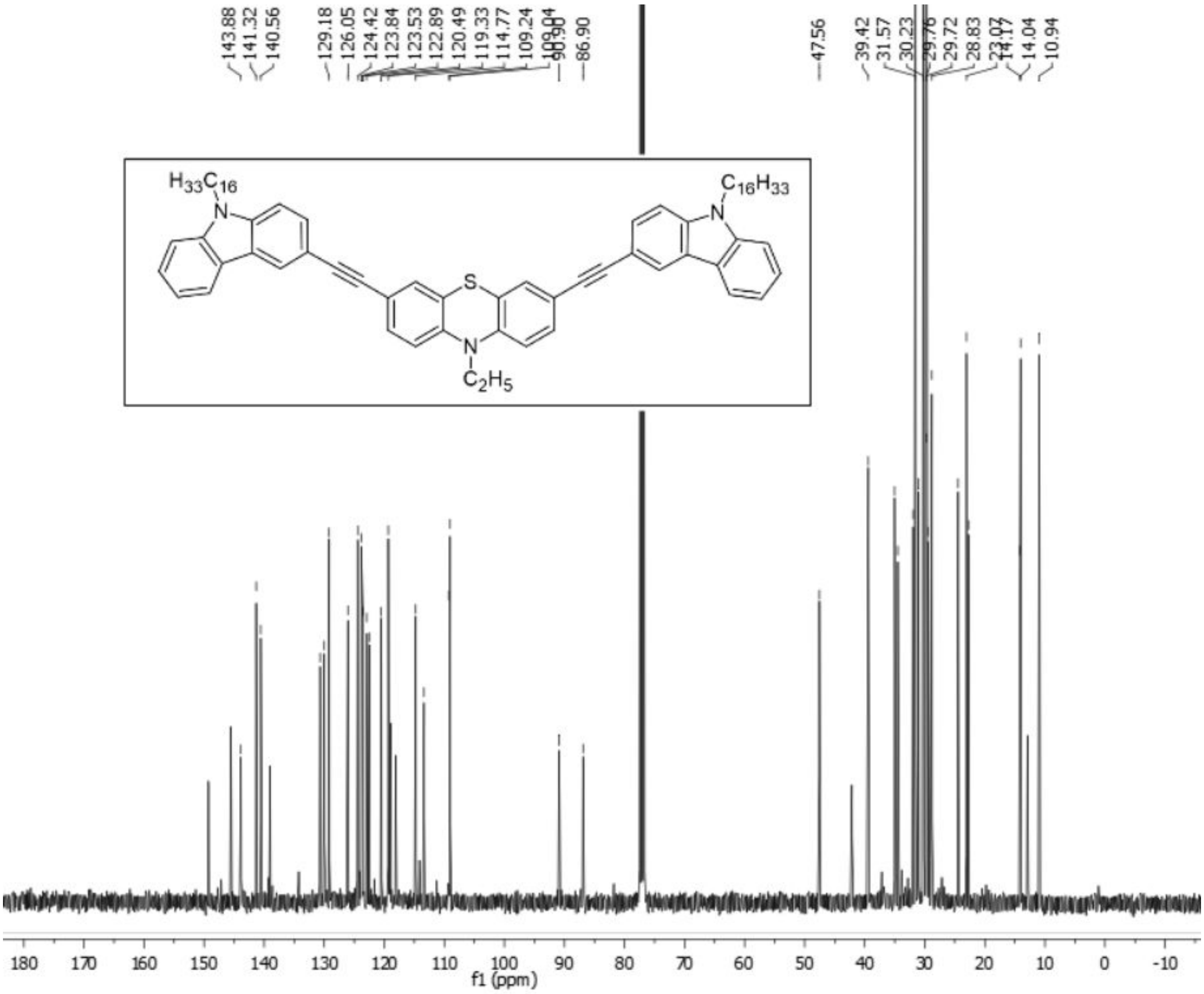

Figure S29. The ^{13}C spectrum of **4g** in $CDCl_3$.

References:

[1] P. Bujak, I. Kulszewicz-Bajer, M. Zagorska, V. Maurel, I. Wielgus and A. Proń, Chem. Soc. Rev. 2013, 42, 8895–8999.
